# Comprehensive analysis of the editing window of C-to-T TALE base editors

**DOI:** 10.1038/s41598-024-63203-8

**Published:** 2024-06-04

**Authors:** Maria Feola, Sylvain Pulicani, Diane Tkach, Alex Boyne, Robert Hong, Louisa Mayer, Aymeric Duclert, Philippe Duchateau, Alexandre Juillerat

**Affiliations:** 1 Cellectis Inc, New York, NY USA; 2 Cellectis, Paris, France

**Keywords:** Gene editing, Base editors, TALE, T-cells, Genetic engineering, Molecular engineering

## Abstract

One of the most recent advances in the genome editing field has been the addition of “TALE Base Editors”, an innovative platform for cell therapy that relies on the deamination of cytidines within double strand DNA, leading to the formation of an uracil (U) intermediate. These molecular tools are fusions of transcription activator-like effector domains (TALE) for specific DNA sequence binding, split-DddA deaminase halves that will, upon catalytic domain reconstitution, initiate the conversion of a cytosine (C) to a thymine (T), and an uracil glycosylase inhibitor (UGI). We developed a high throughput screening strategy capable to probe key editing parameters in a precisely defined genomic context *in cellulo*, excluding or minimizing biases arising from different microenvironmental and/or epigenetic contexts. Here we aimed to further explore how target composition and TALEB architecture will impact the editing outcomes. We demonstrated how the nature of the linker between TALE array and split DddAtox head allows us to fine tune the editing window, also controlling possible bystander activity. Furthermore, we showed that both the TALEB architecture and spacer length separating the two TALE DNA binding regions impact the target TC editing dependence by the surrounding bases, leading to more restrictive or permissive editing profiles.

## Introduction

The Transcription Activator-Like Effector Base Editor are chimeric proteins that catalyze the deamination of either a cytosine to uracil, or an adenine inosine, leading to either a C-to-T or A-to-G conversion respectively^1–3^. These designer base editors rely on the DNA targeting domain from TALE that have been extensively studied during the past decade^4–8^. C-to-T TALE_Base Editors take advantage of the peculiar DNA double strand deaminase activity of a split interbacterial toxin DddAtox from Burkholderia cenocepacia^2,9^. Deamination of the targeted cytosine happens upon reconstitution of the split deaminase through binding of the two TALE fusions. In addition, an uracil glycosylase inhibitor (UGI) domain was fused to this construct to further increase the downstream C-to-T conversion^2^.

Since their first description in 2020, C-to-T TALE_Base Editors and more recently Zinc Finger based editors represented a breakthrough in the gene editing field by their capacity to edit both, the nuclear genome^10^ and the mitochondrial^11^ or chloroplast genome^12^, the latter two being today out of reach for CRISPR/Cas base editors. These C-to-T base editors have been extensively used to introduce genomic modifications/mutations in cellular and animal models^11,13,14^ but might present limitations in the setting for treatment of genetic diseases^3^. One constraint, the requirement of the DddAtox enzyme to have the targeted cytosine in a 5ʹ-TC context, has been partially overcome recently through either protein engineering of the original DddAtox^11^ or the discovery of DddA homolog^15^, overall relaxing the TC requirement to HC and DC respectively. Another potential limitation that is inherent to all base editors, independently of their DNA targeting platform, resides in the editing of one or more bases in addition to the targeted cytosine within the activity window, defined as bystander bases^3,11,16–18^. In view of these challenges, understanding the key aspect of editor designs and drivers of unintended editing byproducts is of foremost importance for gene editing applications in clinical therapies.

To assess the potential of these molecular tools, we took advantage of a recently reported system allowing medium to high throughput screening of TALE_Base Editors *in cellulo*^19^, in a defined genetic environment. Such a cell-based assay enables the exploration of the impact on editing efficiency from/by the interplay between three parameters: the architecture, the spacer length (sequence separating the two TALE binding site) and the sequence composition surrounding the targeted TC. Here, we demonstrate that the nature of the domain linking the TALE binding domain and the split deaminase allows to tune C-to-T conversion within the editing window. We further highlight that the bases composition surrounding the TC to be edited can strongly impact editing efficiencies. The educated choice of an improved architecture referred as “TALEB”, and positioning (spacer length) can either help to prevent such sequence limitation (increase targetable sequence space, relaxed design) or conversely, be used to decrease, if not eliminate (constraint design), bystander editing within the editing window, allowing for more precise genome editing outcomes. Overall, we believe that the knowledge obtained in this study will allow to better design efficient TALEB while improving the specificity profiles of this innovative editing platform.

## Results

### Design of new TALEB architectures and experimental screening setup

**Figure 1:**
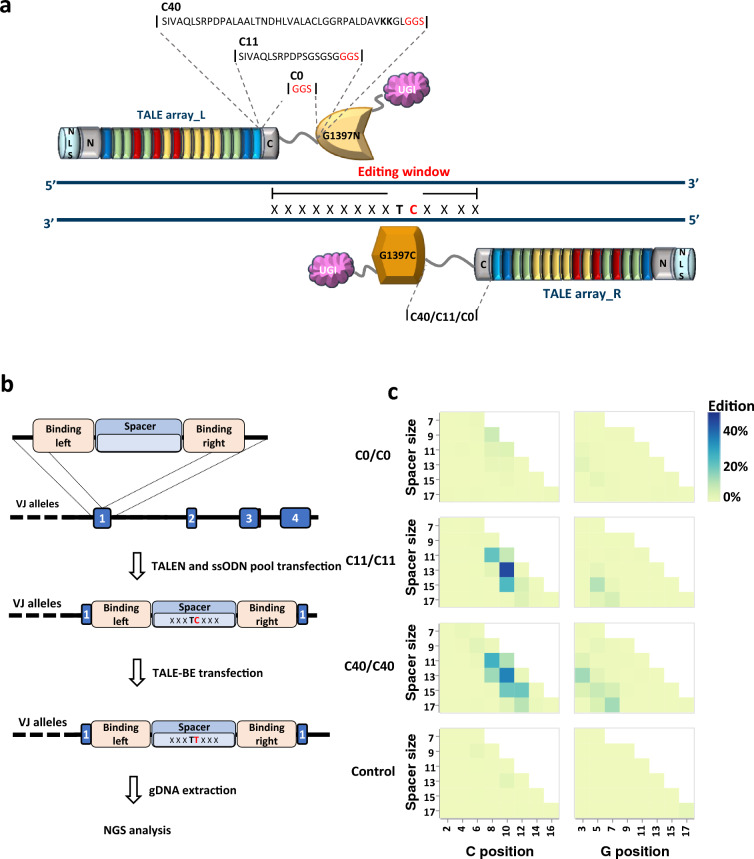
(**a**) Schematic representation of a TALEB. NLS: nuclear localization signal, N: TALE N-terminal domain, G1397N: Split DddAtox N-terminal domain, G1397C: Split DddAtox C-terminal domain, UGI: uracil glycosylase inhibitor. (**b**) Scheme of the strategy to generate artificial base editor target sites. In a first step a pool of ssODN encoding various base editor spacer sequences is inserted into *TRAC* locus. In a second step the TALEB is transfected. Two days post transfection the genomic DNA is collected, and the inserted sequence is analyzed by NGS. (**c**) Heatmap of C-to-T conversion measured on the target collections with spacer spanning from 5 to 17 bp. Right: targeted C on the top strand, Left: targeted C on the bottom strand (N = 1 T-cells donor).

Previous works have pointed towards the positioning of targeted cytosine to be a key determinant for efficient editing. Indeed, analysis of the best editing activity as a function of the TC position within an optimal 13–17 bp spacer length window, highlighted a defined 4–5 bp editing window on both DNA strands^2,19^. To extend our understanding of key determining factors allowing efficient TALEB editing (C-to-T conversion), we investigate whether the nature (length and composition) of the linker that connect the TALE array with the split deaminase catalytic heads, the so-called 1397 split used in this study^11^, could impact C-to-T conversion within the editing window. We envision that shortening this linker region could modify the “reachable space” by the reconstituted DddAtox, and so tune the activity and specificity profiles of TALEB. The linker sequence originally reported for TALEB derives from mitoTALEN^20^, a TALE based nuclease targeting the mitochondria. This linker was composed of the native first 40 amino acids from the C-terminal domain of a TALE from Xanthomonas (AvrBs3, accession nbr P14727) used in TALEN^®^^6,7,21^ to which a short GGS sequence was appended (this scaffold will further be called C40) (Fig. 1a). In addition to the C40 scaffold several other truncations of the C-terminal domain have been reported for TALEN^®^, including one containing only the first 11 amino acids of the TALE C-terminal domain followed by a SGSGSGGGS flexible linker (C11 scaffold, Fig. 1a). This truncation was shown to maintain high nuclease activities while favoring a narrower spacer length reachable sequence space^7^. To better evaluate the importance of the linker length role in a TALEB context, we designed and tested this shorter linker (C11 scaffold), as well as a so called C0 scaffold, where the C-terminal domain was completely removed (maintaining only the GGS linker, Fig. 1a). Noteworthy, two lysine residues that were shown to create non-specific interactions with the DNA (KK, in positions 37 and 38 of the TALE native C-terminal domain^21^, were therefore eliminated in these two shorter scaffolds (Fig. 1a).

To allow for comprehensive studies of key factors impacting C-to-T TALEB, we decided to use a medium to high throughput system that we previously reported (Fig. 1b)^19^. In this setting, a pool of ssODN containing the TALEB 5ʹ-TC target (target of the DddAtox deaminase), either on the top or bottom strand (Supplementary Table 1, Supplementary Table 2 and Supplementary Table 3) is precisely integrated into a predefined genomic locus. To achieve this, the ssODNs contain, at both extremities, sequences (50 bp) homologous to the targeted locus, within the first exon of the *TRAC* gene. Primary T-cells are transfected simultaneously with mRNAs encoding a TALEN targeting the first exon of the *TRAC* gene^19,22^ and along with the ssODN collection, leading to the targeted insertion of the collection of sequences into the nuclear genome. In a second step, mRNAs encoding the TALEB (Supplementary Table 4) are then transfected 2 days post transfection of the TRAC TALEN and ssODN pool. This setup allows for the unbiased binding of the TALEB arm to the artificial target sites, excluding editing variability caused by different DNA binding affinities of different TALE array proteins as well as the impact of epigenomic factors^19^. Additionally, to facilitate the sequence analysis, a unique barcode was added to each ssODN of the pool, at the 3ʹend of the TALEB target site (Supplementary Fig. 1a).

### The nature and length of the targeted sequence spacer influence the C-to-T conversion by TALEB

At first, we decided to compare editing efficiencies (C-to-T conversion) on targets containing a unique 5ʹ-TC within a spacer spanning from 5 to 17 bp by a TALEB containing the C0, C11 or C40. As varying three parameters in parallel (spacer length, position of the TC within the spacer and TC within the top or bottom strand) increased the experimental complexity, we decided to limit our test to odd spacer length, sliding a TCGA quadruplex along the spacer, allowing us to look at both strands at the same time (Supplementary Fig. 1a).

Following the QC analysis (Supplementary Fig. 2a), the analysis of the molecular event (C-to-T conversion) promoted by TALEB within the spacer region further showed absent or very low editing (C counted starting from the left side of the spacer; max editing values: C40: C_4_ 1.3%; C11: C_2_ 3.3%; C0: C_6_ 1%), on both top and bottom strand, for spacer length below 9 bp for any of the three linker pairs (Fig. 1c). The C40 and C11 TALEB architecture combinations demonstrated editing to some extent on the 9 and 17 bp spacer (9 bp spacer, top strand max editing value: C40: C_6_ 3.1%; C11: C_8_ 0.6%; C0: C_8_ 7.3%; bottom strand max editing value: C40: C_5_ 1.9%; C11: C_7_ 0.3%; C0: C_7_ 0.4%; 17 bp spacer, top strand max editing value: C40: C_12_ 10.2%; C11: C_12_ 4.1%; C0: C_16_ 0.6%; bottom strand max editing value: C40: C_7_ 13.1%; C11: C_7_ 5.9%; C0: C_13_ 0.7%). On the spacer of 11bp, these two TALEB architecture combinations showed similar editing levels almost exclusively on the top strand (max editing: C40: C_8_ 25%; C11: C_8_ 21%), while the highest editing rates were obtained on 13 bp and 15bp spacers (max editing: C40: C_10_ 35%; C11: C_10_ 44% on 13bp; C40: C_12_ 20%; C11: C_10_ 24% on 15bp, Fig. 1c). We further noticed that shortening the spacer from 15 to 13bp increased editing (Fig. 1c). Interestingly, shortening the spacer did not allow to rescue activity for the C0, maybe due to a reduced flexibility of this architecture, preventing reassociation of the split deaminase.

Overall, the first datasets confirmed the importance of the spacer length and TC position on editing efficiency and demonstrated the possibility to modulate editing within the spacer and even between the two DNA strands, by using different combinations TALEB architectures.

### Nucleotides surrounding the 5ʹ-TC impact editing differently depending on the architecture

**Figure 2:**
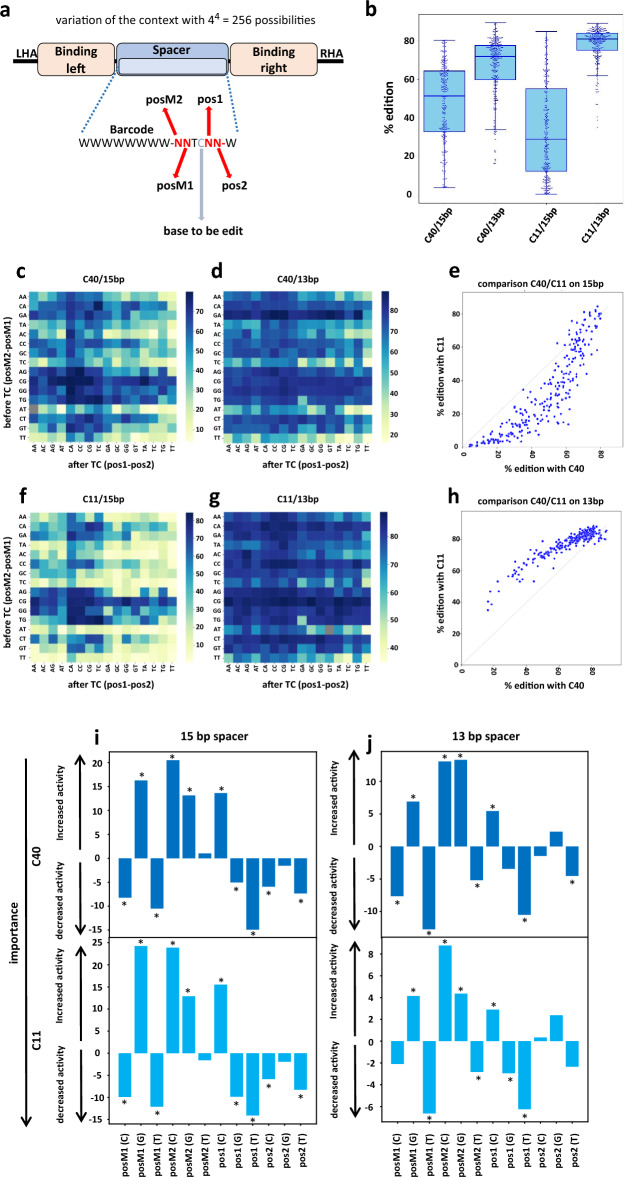
(**a**) Schematic representation of the ssODN pool collection with spacer length of 15 or 13 bp. ssODNs are characterized by TRAC homologies arms (LHA and RHA) for the insertion within the TRAC locus; TALEB binding sites recognized by the TALE DNA targeting domain of the base editor; WWWWWWWW—W represent a 9 bp barcode to improve the identification the integrated oligonucleotide (W = A/T). All barcodes have at least a difference of 2 nucleotides with each other. All combination of the 4 bases immediately surrounding the targeted TC (NNTCNN, 4^4^ = 256 possibilities) were assessed for a spacer of 13 and 15 bp. (**b**) Analysis of C-to-T conversions on the 256 possible targets for the 13 and 15 bp spacer using the C11 and C40 TALEB. Median (middle line), interquartile (box) and 1.5 times the interquartile (whiskers) are represented, as well as the points corresponding to individual targets. (**c**) Heatmap of C-to-T conversion measured on the 15 bp spacer collection with C40 TALEB. (**d**). Heatmap of C-to-T conversion measured on the 13 bp spacer collection with C40 TALEB. (**e**) Comparison of C-to-T conversion between the C11 and C40 TALEB on the 15 bp spacer collection. (**f**) Heatmap of C-to-T conversion measured on the 15 bp spacer collection with C11 TALEB. (**g**) Heatmap of C-to-T conversion measured on the 13 bp spacer collections with C11 TALEB. (**h**) Comparison of C-to-T conversion between the C11 and C40 TALEB on the 13 bp spacer collection. For all panels: N = 2, independent T-cells donors. (**i**) Graphical representation of the importance of each base in the different positions for C40 and C11 scaffold in the 15 bp spacer. A linear model was computed (see “Material and methods”). Each bar represents the coefficient of the linear model showing the effect of the given nucleotide at the given position compared to A at this position chosen as the reference. A positive value means higher activity compared to A, a negative value means lower activity compared to A. Stars indicate values that are statistically significant (p < 0.01). posM1: position -1 compared to TC, posM2: position − 2 compared to TC, pos1: position + 1 compared to TC, position2 : position + 2 compared to TC. (**j**) similar graph as graph (**i**) for C40 and C11 scaffold in the 13 bp spacer.

We next hypothesized that the TALEB architecture and/or spacer length could create constraints to the reassembly of the of DddAtox and its access to the target sequence, leading to greater sequence context dependence. To provide a more detailed analysis of possible short range context dependence of bases surrounding the 5ʹTC, we designed new collections of targets containing a single TC at position 4 (position of the C), which represent a good compromise to obtain high editing efficiencies on spacers of 13 and 15 bp. The four bases surrounding the TC (two bases upstream and two bases downstream) were fully randomized (256 members in each collection, Supplementary Table 2, Supplementary Table 3; Fig. 2a) and the C-to-T conversion of these target collections was monitored using the C11 and C40 architectures. As for the previous collections, NGS analysis showed sufficient target integration at the TRAC locus for all or nearly all 256 combinations to reliably quantify their editing (Supplementary Fig. 3a–d) with background editing in the no TALEB control whereas the samples treated with C40 and C11 TALEB showed detectable and reproducible levels of C-to-T conversion over two independent dataset (two T-cell donors) (Supplementary Fig. 3e–h).

Overall editing on the 13 bp collection (Median: 72% for C40, 81% for C11) was found higher when compared to the 15 bp collection (median: 51% for C40, 29% for C11) which was expected from the slightly more favorable positioning of the TC (position C_4_) within the former spacer length (Fig. 2b,c,d,f,g).

For the 15bp spacer target collections, the comparison of editing frequencies between the TALEB architectures (C11 and C40) showed a non-linear correlation (Fig. 2e). For contexts considered as less favorable to editing (as defined by targets that belong neither to the top 50 for C40 nor to the top 50 for C11), editing with the C11 architecture was found to be lower than for the C40 scaffold (median ratio of C40 to C11 = 2.08, n = 192 targets). For contexts that were most favorable to editing (targets that belong to both top 50 for C40 and top 50 for C11), the activity was similar for both architectures (median ratio of C40 to C11 = 1.0, n = 37 targets) and reached up to 80% C-to-T conversion (Fig. 2b and e). Surprisingly, opposite to what was observed on the 15 bp spacer, the C11 showed less context dependence editing compared to the C40 combination (Fig. 2f,g,h). Overall, when considering the 15 bp spacer, analysis showed similar nucleotides preferences for both architectures (posM2 C11 and 40: A = T <  < G < C; posM1 C11 and 40: T = C < A <  < G; pos1 C11:40: T < G < A <  < C; pos2 C11 and 40: T < C < G < A, Fig. 2i, Supplementary Fig. 3i–l); a positive value means higher activity compared to A, a negative value means lower activity compared to A). Similar context preferences between both architectures was also observed on the 13 bp spacer (posM2 C11: T < A < G < C; posM2 C40: T < A <  < G = C, posM1 C11 and 40: T < C < A < G; pos1 C11 and 40: T < G < A < C; pos2: T < A = C < G, Fig. 2j, Supplementary Fig. 3m–p), but the context tended to be less stringent for editing relative to the 15 bp spacers (Fig. 2i and j).

Overall, this second datasets confirmed the importance of composition of the surrounding bases, and how it can impact editing outcomes. Results showed what it appears to be an addictive effect per-base, which can help predict influence of the sequence context (Supplementary Fig. 4a–d; Supplementary Table 6).

### Adequate TALEB architecture choice could limit unwanted multiple editing on cytosine stretches

As we previously observed that the C11 architecture presented a more discriminant editing pattern, we hypothesized that using this architecture could prevent or limit unwanted bystander editing, especially within stretches of cytosines directly following the TC.

**Figure 3:**
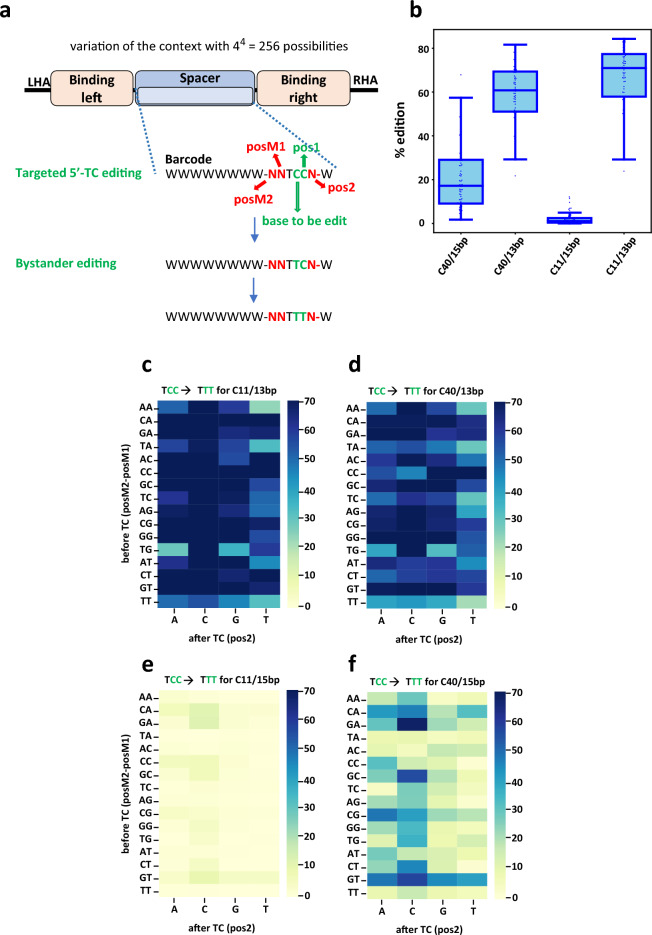
(**a**) Schematic representation of editing of a C in pos1 in the context of a NNTCCN target. (**b**) Analysis of C-to-T conversions on the C following the targeted TC for the 15 and 13 bp spacer using the 285 C40 and C11 TALEB. Median (middle line), interquartile (box) and 1.5 times the interquartile (whiskers) are represented, as well as the points corresponding to individual targets. (**c**) Heatmap of C-to-T conversion on C following the central TC, measured on the 15 bp spacer collection with C40 TALEB. (**d**) Heatmap of C-to-T conversion on C following the central TC, measured on the 15 bp spacer collection with C11 TALEB. (**e**) Heatmap of C-to-T conversion on C following the central TC, measured on the 13 bp spacer collection with C40 TALEB. (**f**) Heatmap of C-to-T conversion on C following the central TC, measured on the 13 bp spacer collections with C11 TALEB. For all panels: N = 2, independent T-cells donors.

In order to analyze possible bystander editing in position − 3, − 2, + 1 and + 2 we first look at classical C40 architecture NNTCNN combinations presenting more than 30% editing rate on both 13 bp and 15 bp collection. Among these, we filtered those for which at least 20% of the reads had editing other than on the central TC. We noticed that the most frequent edited cytosine was not always the sole mutation of the targeted 5ʹ-TC but rather multiple mutations. In the latest case, the vast majority were detected when the 5ʹ-TC was immediately followed by another Cs (NNTCCN and NNTCCC). Editing of the central C into a T most probably favored further editing of the following C (Fig. 3a). We next look at the editing frequencies from NNTCCN to NNTTTN, for both C40 and C11 architecture on both 13 and 15 bp spacer length. Comparison of the editing results showed clear differences in the C-to-T conversion rates on the pos2 between the four conditions (Fig. 3b). On a spacer of 13 bp, both the C11 and C40 architecture showed a permissive profile with high rates of editing on this position (C11: 70.96 ± 14.53, C40: 60.78 ± 13.74, median ± stddev, Fig. 3c and d), while on the 15 bp spacer both architectures showed more restrictive editing (C11: 1.09 ± 2.68, C40: 17.21 ± 15.41, median ± stddev, Fig. 3e and f). The use of the C11 architecture on this later spacer almost abolished the editing in most contexts, revealing the possibility to prevent (bystander) edits on stretches of multiple cytosines. For all the tested conditions, the two architectures show different nucleotides preferences within the different spacers (Supplementary Fig. 5a–e).

Overall, the datasets presented in this study highlighted how three key factors, spacer length, TALEB architecture and composition of the surrounding bases can impact editing outcomes but also demonstrated the possibility to tune and control editing using educated designs. Taken together, these results revealed, in particular, the primordial importance of the positions preceding the targeted TC, in which the presence of a G or a A as a base immediately preceding (posM1) does markedly increases editing efficiency. We thus proposed the following guidance to prioritize the definition of target sites for TALEB: 5ʹ–**T**_0_-N_left_-**N**_**y**_**-RTC-N**_**X**_-N_right_-**A**_0_–3ʹ. With T_0_ (and A_0_) representing the first nucleotide of the target sequence (targeted by the N-terminal domain of the TALE), N_left_ and N_right_ being the sequence targeted by the repeat DNA binding core (RVDs), R being an A or a G, preferably a G. N_y_ and N_x_ could vary, with x = 2 to 6 nucleotides (optimally 2 or 3) and y = 6 to 10 nucleotides, with x + y = 12 for a 15 bp spacer (or x + y = 10 for a 13 bp spacer with increased tolerance to the nature of surrounding bases).

However, it needs to be noted that the dataset reported here represents a snapshot of editing 48 h post TALEB transfection and that differences in editing kinetics between the architectures and spacer length could have contributed to the differential context dependence that was observed. As a consequence, one limitation of our study resides in the fact that we cannot exclude the possibility of attenuated differences between the two architectures at later time points, once the overall editing is completely saturated.

## Discussion

Base editors as molecular tools have been widely used to target and edit several type of genetic elements (e.g. enhancers^23^, start codons^24^, splice sites and branch points^25,26^ or pathogenic mutations^27–29^. However, the success of their use for therapeutic application will largely rely upon our capacity to perform the extremely precise introduction of an intended mutation while minimizing or abrogating possible, bystander (editing at other positions than the intended one) and byproduct (editing different from the expected C-to-T) edits. Although, it should be noted, this may be less of a concern for applications where gene disruption is required.

In this study we characterized the base editing profiles of TALE-linked C-to-T base editors. Cytosine deamination, the first step of the cytosine to thymine conversion, is carried out by a domain (DddAtox) of an interbacterial toxin. Unlike other previously described engineered designer deaminases that are primarily/only acting on ssDNA, the DddAtox domain allows deamination directly within dsDNA. In order to avoid toxicity linked to the expression of an intact DddAtox domain, Liu and colleagues^2^, split the DddAtox into non-toxic halves. This first generation of TALE base editors relied on the use of a TALE nuclease (TALEN) scaffold fused with the split DddAtox deaminase, overall forming a heterodimeric designer base editor.

One key aspect in engineering such chimeric protein resides in the linker connecting the engineered DNA binding domain to the catalytic domain. It has been shown that, in multiple FokI based nuclease contexts (e.g. TALEN or, ZFN, Guilinger et al.^21^; Juillerat et al.^7^) that this linker domain is a key parameter for the conception (e.g.: distance between the two-half nuclease) of such designer nuclease. Recently, Liu and colleagues^30^ demonstrated that the editing outcomes by ZF-DdCBE was impacted by the linker connecting the ZF array and a split DddA deaminase, supposedly by affecting the capacity of the split DddAtox to reassemble or by constraining the access of the target sequence to the reassembled DddAtox. In this study we further demonstrated that in the context of a TALEB, the nature of the domain linking the TALE DNA binding domain and the split DddAtox catalytic domain not only impacted the editing efficiency but also other critical editing outcomes. Indeed, at similar editing levels, the C11/C11 architecture provided a more restricted/narrower editing profile within the spacer window.

In their original work, Liu and colleagues^2^, reported the probability sequence logo of the region flanking mutated cytosines in *E. coli* strains following exposure to the monomeric DddAtox domain, highlighting a strong preference for the 5ʹTC context but relatively low influence of the surrounding bases. Here, however, we showed that, in the context of a split DddAtox (1397 split, Mok et al.^2^) fused to TALE DNA binding domains, the context surrounding the 5ʹTC may severely impact the editing rates. We identified the distance between the two binding domains (spacer) as a key driver to the sensitivity to surrounding bases, a shorter 13 bp and longer 15 bp spacer being more permissive or restrictive respectively. The nature of the linker domain between the TALE DNA binding domain and the catalytic heads further modulated the sensitivity to the sequence context. These findings were in accordance with a study from Kim and colleagues^31^ who demonstrated different C-to-T conversion selectivity, within the spacer, between a monomeric TALEB (DddAtox containing attenuating mutations) and the dimeric (split) TALEB. By design the split DddAtox architecture, requiring the dimerization of the two half catalytic domains, would have parameters impacting dimerization or constraining access to the target sequence (eg spacer length or linker composition), that would also affect target sequence preferences. Nor can it be excluded that DddAtox mutants (or homologues) recently described to (i) modify the dimerization interface and reconstitution of the split DddAtox^32,33^, (ii) to attenuate direct interaction of the DddAtox with DNA^11^ or (iii) to relax the 5ʹTC limitation (Mok et al.^11^ MiNatComm2023) would also present different or novel editing constraints and profiles.

The experimental strategy used in this study to characterize editing profiles in depth and in a high throughput format can easily be applied to any new editors to continue expanding this platform for potential therapeutic applications. Nevertheless, while in this study TALEB have been delivered as mRNA, we acknowledge that longer exposure to editors using, for example plasmids, might diminish, or erase, some of the observed differences. However, we do believe that the data presented here are still of great interest as it is a general trend in the field of Gene Editing to prefer transient rather than long-term expression of gene editors to minimize potential adverse effects such as off-site editing. mRNA reagents, as used in our study, represent the vector of choice for such a goal, thus our data will help with designing gene editors in this specific context. While additional studies will need to be carried out to further define the possibilities of new DddAtox (or homolog-derived) base editors, we believe that the knowledge obtained here will allow to better design more efficient TALEB while also improving their specificity profiles.

## Material and methods

### T cell culture

Cryopreserved human PBMCs were acquired from ALLCELLS. PBMCs were cultured in X-vivo-15 media (Lonza Group), containing 20 ng/ml human IL-2 (Miltenyi Biotec), and 5% human serum AB (Seralab). Human T cell activator TransAct (Miltenyi Biotec) was used to activate T cells at 25 µl TransAct (Miltenyi Biotec) per million CD3+ cells the day after thawing the PBMCs. TransAct (Miltenyi Biotec) was kept in the culture media for 72 h.

### TALE-Nuclease and TALEB mRNA production

Plasmids encoding the TRAC TALE-Nuclease contained a T7 promoter and a ~ 120 polyA sequence. The TALE-Nuclease mRNA from the TRAC TALE-Nuclease plasmid was produced by Trilink. The sequence targeted by the TRAC TALE-Nuclease (17-bp recognition sites, upper case letters, separated by a 15-bp spacer) is provided in Supplementary Table 5.

Plasmids encoding TALEB contained a T7 promoter and a ~ 120 polyA sequence. Sequence verified plasmids were linearized with SapI (NEB) before in vitro mRNA synthesis. mRNA was produced with NEB HiScribe™ T7 Quick High Yield RNA Synthesis Kit (NEB). The 5ʹcapping reaction was performed with ScriptCap™ m7G Capping System (Cellscript). Antarctic Phosphatase (NEB) was used to treat the capped mRNA and the final cleanups was performed with Mag-Bind TotalPure NGS beads (Omega bio-tek) and Invitrogen DynaMag-2 Magnet (ThermoFisher).

### ssODN repair template transfection

The ssODN pool targeting the TRAC locus (Supplementary_Table 1, Supplementary Table 2, and Supplementary Table 3) were obtained from Integrated DNA Technologies (IDT) and resuspended in ddH_2_O at 50 pmol/µl.

T cells activated with TransAct (Miltenyi Biotec) for 3 days were transferred into fresh complete media containing 20ng/ml human IL-2 (Miltenyi Biotec), and 5% human serum AB (Seralab) 10-12h before transfection.

The harvested cells were washed once with warm PBS. 1E6 PBS washed cells were pelleted and resuspended in 20 µl Lonza P3 primary cell buffer (Lonza). 200 pmol ssODN pool and 1 mg/arm of TRAC TALE-Nuclease were mixed with the cell and then the cell mixture was electroporated using the Lonza 4D-Nucleofector under the EO115 program for stimulated human T cells. After electroporation, 80 µl warm complete media was added to the cuvette to dilute the electroporation buffer, the mixture was then carefully transferred to 400 ml pre-warmed complete media in 48-well plates. Cells transfected with ssODN and TALE-Nuclease were then incubated at 30 °C until 24 h post TALE-Nuclease transfection before transfer back to 37 °C.

Cells with ssODN KI were cultured for 2 days before harvesting for TALEB treatment. The harvested cells were washed once with warm PBS. 1E6 PBS washed cells were pelleted and resuspended in 20 µl Lonza P3 primary cell buffer (Lonza). 1 mg/arm of TALEB (C0, C11 or C40) were mixed with the cell and then the cell mixture was electroporated using the Lonza 4D-Nucleofector under the EO115 program for stimulated human T cells. After electroporation, 80 µl warm complete media was added to the cuvette to dilute the electroporation buffer, the mixture was then carefully transferred to 400 ml pre-warmed complete media in 48-well plates. Cells transfected with TALEB incubated at 37 °C for 2 more days before harvesting for gDNA extraction and NGS analysis.

### Genomic DNA extraction

Cells were harvested and washed once with PBS. Genomic DNA extraction was performed using Mag-Bind Blood & Tissue DNA HDQ kits (Omega Bio-Tek) following the manufacturer’s instructions.

### Targeted PCR and NGS

100 ng genomic DNA was used per reaction in a 50 ml reaction with Phusion High-Fidelity PCR Master Mix (NEB). The PCR condition was set to 1 cycle of 30 s at 98 °C; 30 cycles of 10 s at 98 °C, 30 s at 60 °C, 30 s at 72 °C; 1 cycle of 5 min at 72 °C; hold at 4 °C. The PCR product was then purified with Omega NGS beads (1:1.2 ratio) and eluted into 30 ml of 10 mM Tris buffer pH7.4. The second PCR which incorporates NGS indices was then performed on the purified product from the first PCR. 15 µl of the first PCR product were set in a 50 ml reaction with Phusion High-Fidelity PCR Master Mix (NEB). The PCR condition was set to 1 cycle of 30 s at 98 °C; 8 cycles of 10 s at 98 °C, 30 s at 62 °C, 30 s at 72 °C; 1 cycle of 5 min at 72 °C; hold at 4 °C. Purified PCR products were sequenced on MiSeq (Illumina) on a 2 × 250 V2 cartridge.

### Amplicon-sequencing analysis

The sequences from the amplicon-seq were aligned on the Human genome (release GRCh38). The TALE binding sequences were used as anchors to extract the spacer sequences. These spacer sequences were compared to the WT spacers, to get the CG position. Then, we looked at the mutations in the spacer to classify the sequences. Indeed, for a C>T and/or G>A, the spacer was kept as *edited* if it had zero or one mutation other than the CG. If the CG wasn't mutated, we kept the spacer as *not edited* if it had zero or one mutation other than the CG. If the sequence had the C mutated in something else than a T and/or the G mutated in something else than an A, we kept the spacer as *mutated* if it had zero or one mutation other than the CG. Finally, we didn't find *indels* in the spacers. After doing that, we grouped the sequences by spacer size and CG position and computed the frequency of *edited* ones.

### Statistical analysis

To analyze the role of each of the 4 positions surrounding the TC in the editing activity, a linear model was computed, taking at each position A as a reference, using the stats model’s library from python. Most of the terms of this model had a coefficient that was statistically different from 0 (p-value below 0.01). Coefficients of the model are displayed in Fig. 2i and j.

### Supplementary Information

Supplementary Tables.

Supplementary Figures.

## Data Availability

The datasets used and/or analyzed during the current study available from the corresponding author on reasonable request.
