# Supplementary material for: Comprehensive analysis of the editing window of C-to-T TALE base editors
Source: Sci Rep. 2024 Jun 4;14:12870. doi: 10.1038/s41598-024-63203-8 (PMC11150444; doi:10.1038/s41598-024-63203-8)
Supplement: Supplementary file 1 — Supplementary Tables. [file 41598_2024_63203_MOESM1_ESM.docx]

**Supplementary Table 1. Sequences of the pool of ssODN with spacer lengths of 17, 15, 13, 11, 9, 7 or 5 bp with a TCGA moving along the spacer every two positions (from left to right: purple=TRAC left homology arm; pink= STAT3 left binding site; red= targeted sequence; orange= STAT3 right binding site; black= barcode; green TRAC right homology arm).**

| Well Position | Name | Sequence |
| --- | --- | --- |
| A1 | TCGA_1 | GAGATCATGTCCTAACCCTGATCCTCTTGTCCCACAGATATCCAGAACCCTCTAAGAAGTTCCTGCTTCGATGAATGTGGTTAGAGACAAAACAGTGACCCTGCCGTGTACCAGCTGAGAGACTCTAAATCCAGTGACAAGTCTG |
| B1 | TCGA_2 | GAGATCATGTCCTAACCCTGATCCTCTTGTCCCACAGATATCCAGAACCCTCTAAGAAGTTCCTGCTATCGAGAATGTGGTTAGAGACAAAACCTTGACCCTGCCGTGTACCAGCTGAGAGACTCTAAATCCAGTGACAAGTCTG |
| C1 | TCGA_3 | GAGATCATGTCCTAACCCTGATCCTCTTGTCCCACAGATATCCAGAACCCTCTAAGAAGTTCCTGCTAATCGGAATGTGGTTAGAGACAAAACGATGACCCTGCCGTGTACCAGCTGAGAGACTCTAAATCCAGTGACAAGTCTG |
| D1 | TCGA_4 | GAGATCATGTCCTAACCCTGATCCTCTTGTCCCACAGATATCCAGAACCCTCTAAGAAGTTCCTGCTGAATCGAATGTGGTTAGAGACAAAACGGTGACCCTGCCGTGTACCAGCTGAGAGACTCTAAATCCAGTGACAAGTCTG |
| E1 | TCGA_5 | GAGATCATGTCCTAACCCTGATCCTCTTGTCCCACAGATATCCAGAACCCTCTAAGAAGTTCCTGCTTCGATATGAATGTGGTTAGAGACAAAACTCTGACCCTGCCGTGTACCAGCTGAGAGACTCTAAATCCAGTGACAAGTCTG |
| F1 | TCGA_6 | GAGATCATGTCCTAACCCTGATCCTCTTGTCCCACAGATATCCAGAACCCTCTAAGAAGTTCCTGCTAATCGATGAATGTGGTTAGAGACAAAAGACTGACCCTGCCGTGTACCAGCTGAGAGACTCTAAATCCAGTGACAAGTCTG |
| G1 | TCGA_7 | GAGATCATGTCCTAACCCTGATCCTCTTGTCCCACAGATATCCAGAACCCTCTAAGAAGTTCCTGCTGATATCAGAATGTGGTTAGAGACAAAAGAGTGACCCTGCCGTGTACCAGCTGAGAGACTCTAAATCCAGTGACAAGTCTG |
| H1 | TCGA_8 | GAGATCATGTCCTAACCCTGATCCTCTTGTCCCACAGATATCCAGAACCCTCTAAGAAGTTCCTGCTTCGAtattaGAATGTGGTTAGAGACAAAAGCTTGACCCTGCCGTGTACCAGCTGAGAGACTCTAAATCCAGTGACAAGTCTG |
| A2 | TCGA_9 | GAGATCATGTCCTAACCCTGATCCTCTTGTCCCACAGATATCCAGAACCCTCTAAGAAGTTCCTGCTtaTCGAtatGAATGTGGTTAGAGACAAAAGGATGACCCTGCCGTGTACCAGCTGAGAGACTCTAAATCCAGTGACAAGTCTG |
| B2 | TCGA_10 | GAGATCATGTCCTAACCCTGATCCTCTTGTCCCACAGATATCCAGAACCCTCTAAGAAGTTCCTGCTattaTCGAtGAATGTGGTTAGAGACAAAAGGGTGACCCTGCCGTGTACCAGCTGAGAGACTCTAAATCCAGTGACAAGTCTG |
| C2 | TCGA_11 | GAGATCATGTCCTAACCCTGATCCTCTTGTCCCACAGATATCCAGAACCCTCTAAGAAGTTCCTGCTAtattaTCGGAATGTGGTTAGAGACAAAAGTCTGACCCTGCCGTGTACCAGCTGAGAGACTCTAAATCCAGTGACAAGTCTG |
| D2 | TCGA_12 | GAGATCATGTCCTAACCCTGATCCTCTTGTCCCACAGATATCCAGAACCCTCTAAGAAGTTCCTGCTTCGAtatttaaGAATGTGGTTAGAGACAAAATCCTGACCCTGCCGTGTACCAGCTGAGAGACTCTAAATCCAGTGACAAGTCTG |
| E2 | TCGA_13 | GAGATCATGTCCTAACCCTGATCCTCTTGTCCCACAGATATCCAGAACCCTCTAAGAAGTTCCTGCTaaTCGAtatttGAATGTGGTTAGAGACAAAATGCTGACCCTGCCGTGTACCAGCTGAGAGACTCTAAATCCAGTGACAAGTCTG |
| F2 | TCGA_14 | GAGATCATGTCCTAACCCTGATCCTCTTGTCCCACAGATATCCAGAACCCTCTAAGAAGTTCCTGCTttaaTCGAtatGAATGTGGTTAGAGACAAACAAGTGACCCTGCCGTGTACCAGCTGAGAGACTCTAAATCCAGTGACAAGTCTG |
| G2 | TCGA_15 | GAGATCATGTCCTAACCCTGATCCTCTTGTCCCACAGATATCCAGAACCCTCTAAGAAGTTCCTGCTatttaaTCGAtGAATGTGGTTAGAGACAAACACCTGACCCTGCCGTGTACCAGCTGAGAGACTCTAAATCCAGTGACAAGTCTG |
| H2 | TCGA_16 | GAGATCATGTCCTAACCCTGATCCTCTTGTCCCACAGATATCCAGAACCCTCTAAGAAGTTCCTGCTAtatttaaTCGGAATGTGGTTAGAGACAAACAGATGACCCTGCCGTGTACCAGCTGAGAGACTCTAAATCCAGTGACAAGTCTG |
| A3 | TCGA_17 | GAGATCATGTCCTAACCCTGATCCTCTTGTCCCACAGATATCCAGAACCCTCTAAGAAGTTCCTGCTTCGAtaattataaGAATGTGGTTAGAGACAAACAGGTGACCCTGCCGTGTACCAGCTGAGAGACTCTAAATCCAGTGACAAGTCTG |
| B3 | TCGA_18 | GAGATCATGTCCTAACCCTGATCCTCTTGTCCCACAGATATCCAGAACCCTCTAAGAAGTTCCTGCTaaTCGAtaattatGAATGTGGTTAGAGACAAACCATTGACCCTGCCGTGTACCAGCTGAGAGACTCTAAATCCAGTGACAAGTCTG |
| C3 | TCGA_19 | GAGATCATGTCCTAACCCTGATCCTCTTGTCCCACAGATATCCAGAACCCTCTAAGAAGTTCCTGCTataaTCGAtaattGAATGTGGTTAGAGACAAACCGTTGACCCTGCCGTGTACCAGCTGAGAGACTCTAAATCCAGTGACAAGTCTG |
| D3 | TCGA_20 | GAGATCATGTCCTAACCCTGATCCTCTTGTCCCACAGATATCCAGAACCCTCTAAGAAGTTCCTGCTttataaTCGAtaaGAATGTGGTTAGAGACAAACGCATGACCCTGCCGTGTACCAGCTGAGAGACTCTAAATCCAGTGACAAGTCTG |
| E3 | TCGA_21 | GAGATCATGTCCTAACCCTGATCCTCTTGTCCCACAGATATCCAGAACCCTCTAAGAAGTTCCTGCTaattataaTCGAtGAATGTGGTTAGAGACAAACTCGTGACCCTGCCGTGTACCAGCTGAGAGACTCTAAATCCAGTGACAAGTCTG |
| F3 | TCGA_22 | GAGATCATGTCCTAACCCTGATCCTCTTGTCCCACAGATATCCAGAACCCTCTAAGAAGTTCCTGCTAtaattataaTCGGAATGTGGTTAGAGACAAACTGGTGACCCTGCCGTGTACCAGCTGAGAGACTCTAAATCCAGTGACAAGTCTG |
| G3 | TCGA_23 | GAGATCATGTCCTAACCCTGATCCTCTTGTCCCACAGATATCCAGAACCCTCTAAGAAGTTCCTGCTTCGAttataattaaaGAATGTGGTTAGAGACAAACTTCTGACCCTGCCGTGTACCAGCTGAGAGACTCTAAATCCAGTGACAAGTCTG |
| H3 | TCGA_24 | GAGATCATGTCCTAACCCTGATCCTCTTGTCCCACAGATATCCAGAACCCTCTAAGAAGTTCCTGCTaaTCGAttataattaGAATGTGGTTAGAGACAAAGAACTGACCCTGCCGTGTACCAGCTGAGAGACTCTAAATCCAGTGACAAGTCTG |
| A4 | TCGA_25 | GAGATCATGTCCTAACCCTGATCCTCTTGTCCCACAGATATCCAGAACCCTCTAAGAAGTTCCTGCTtaaaTCGAttataatGAATGTGGTTAGAGACAAAGAAGTGACCCTGCCGTGTACCAGCTGAGAGACTCTAAATCCAGTGACAAGTCTG |
| B4 | TCGA_26 | GAGATCATGTCCTAACCCTGATCCTCTTGTCCCACAGATATCCAGAACCCTCTAAGAAGTTCCTGCTattaaaTCGAttataGAATGTGGTTAGAGACAAAGACATGACCCTGCCGTGTACCAGCTGAGAGACTCTAAATCCAGTGACAAGTCTG |
| C4 | TCGA_27 | GAGATCATGTCCTAACCCTGATCCTCTTGTCCCACAGATATCCAGAACCCTCTAAGAAGTTCCTGCTtaattaaaTCGAttaGAATGTGGTTAGAGACAAAGACTTGACCCTGCCGTGTACCAGCTGAGAGACTCTAAATCCAGTGACAAGTCTG |
| D4 | TCGA_28 | GAGATCATGTCCTAACCCTGATCCTCTTGTCCCACAGATATCCAGAACCCTCTAAGAAGTTCCTGCTtataattaaaTCGAtGAATGTGGTTAGAGACAAAGAGATGACCCTGCCGTGTACCAGCTGAGAGACTCTAAATCCAGTGACAAGTCTG |
| E4 | TCGA_29 | GAGATCATGTCCTAACCCTGATCCTCTTGTCCCACAGATATCCAGAACCCTCTAAGAAGTTCCTGCTAttataattaaaTCGGAATGTGGTTAGAGACAAAGAGTTGACCCTGCCGTGTACCAGCTGAGAGACTCTAAATCCAGTGACAAGTCTG |
| F4 | TCGA_30 | GAGATCATGTCCTAACCCTGATCCTCTTGTCCCACAGATATCCAGAACCCTCTAAGAAGTTCCTGCTTCGAtattattaaattaGAATGTGGTTAGAGACAAAGATCTGACCCTGCCGTGTACCAGCTGAGAGACTCTAAATCCAGTGACAAGTCTG |
| G4 | TCGA_31 | GAGATCATGTCCTAACCCTGATCCTCTTGTCCCACAGATATCCAGAACCCTCTAAGAAGTTCCTGCTtaTCGAtattattaaatGAATGTGGTTAGAGACAAAGCTGTGACCCTGCCGTGTACCAGCTGAGAGACTCTAAATCCAGTGACAAGTCTG |
| H4 | TCGA_32 | GAGATCATGTCCTAACCCTGATCCTCTTGTCCCACAGATATCCAGAACCCTCTAAGAAGTTCCTGCTattaTCGAtattattaaGAATGTGGTTAGAGACAAAGGAATGACCCTGCCGTGTACCAGCTGAGAGACTCTAAATCCAGTGACAAGTCTG |
| A5 | TCGA_33 | GAGATCATGTCCTAACCCTGATCCTCTTGTCCCACAGATATCCAGAACCCTCTAAGAAGTTCCTGCTaaattaTCGAtattattGAATGTGGTTAGAGACAAAGGAGTGACCCTGCCGTGTACCAGCTGAGAGACTCTAAATCCAGTGACAAGTCTG |
| B5 | TCGA_34 | GAGATCATGTCCTAACCCTGATCCTCTTGTCCCACAGATATCCAGAACCCTCTAAGAAGTTCCTGCTttaaattaTCGAtattaGAATGTGGTTAGAGACAAAGGGATGACCCTGCCGTGTACCAGCTGAGAGACTCTAAATCCAGTGACAAGTCTG |
| C5 | TCGA_35 | GAGATCATGTCCTAACCCTGATCCTCTTGTCCCACAGATATCCAGAACCCTCTAAGAAGTTCCTGCTtattaaattaTCGAtatGAATGTGGTTAGAGACAAAGGGTTGACCCTGCCGTGTACCAGCTGAGAGACTCTAAATCCAGTGACAAGTCTG |
| D5 | TCGA_36 | GAGATCATGTCCTAACCCTGATCCTCTTGTCCCACAGATATCCAGAACCCTCTAAGAAGTTCCTGCTattattaaattaTCGAtGAATGTGGTTAGAGACAAAGGTCTGACCCTGCCGTGTACCAGCTGAGAGACTCTAAATCCAGTGACAAGTCTG |
| E5 | TCGA_37 | GAGATCATGTCCTAACCCTGATCCTCTTGTCCCACAGATATCCAGAACCCTCTAAGAAGTTCCTGCTAtattattaaattaTCGGAATGTGGTTAGAGACAAAGGTGTGACCCTGCCGTGTACCAGCTGAGAGACTCTAAATCCAGTGACAAGTCTG |

**Supplementary Table 2. Sequences of the pool of ssODN with spacer lengths of 15 bp (from left to right: purple=TRAC left homology arm; pink= STAT3 left binding site; black= barcode; red= targeted sequence; orange= STAT3 right binding site; green TRAC right homology arm).**

| Well Position | Name | Sequence |
| --- | --- | --- |
| A1 | TC15P1_pool1_1 | GAGATCATGTCCTAACCCTGATCCTCTTGTCCCACAGATATCCAGAACCCTCTAAGAAGTTCCTGCTTTTAAAATAATCAAAGAATGTGGTTAGAGACATGACCCTGCCGTGTACCAGCTGAGAGACTCTAAATCCAGTGACAAGTCTG |
| B1 | TC15P1_pool1_2 | GAGATCATGTCCTAACCCTGATCCTCTTGTCCCACAGATATCCAGAACCCTCTAAGAAGTTCCTGCTATTTTAATAATCACTGAATGTGGTTAGAGACATGACCCTGCCGTGTACCAGCTGAGAGACTCTAAATCCAGTGACAAGTCTG |
| C1 | TC15P1_pool1_3 | GAGATCATGTCCTAACCCTGATCCTCTTGTCCCACAGATATCCAGAACCCTCTAAGAAGTTCCTGCTATAAATAAAATCAGAGAATGTGGTTAGAGACATGACCCTGCCGTGTACCAGCTGAGAGACTCTAAATCCAGTGACAAGTCTG |
| D1 | TC15P1_pool1_4 | GAGATCATGTCCTAACCCTGATCCTCTTGTCCCACAGATATCCAGAACCCTCTAAGAAGTTCCTGCTTTATATTTAATCATAGAATGTGGTTAGAGACATGACCCTGCCGTGTACCAGCTGAGAGACTCTAAATCCAGTGACAAGTCTG |
| E1 | TC15P1_pool1_5 | GAGATCATGTCCTAACCCTGATCCTCTTGTCCCACAGATATCCAGAACCCTCTAAGAAGTTCCTGCTAATATATTAATCCAAGAATGTGGTTAGAGACATGACCCTGCCGTGTACCAGCTGAGAGACTCTAAATCCAGTGACAAGTCTG |
| F1 | TC15P1_pool1_6 | GAGATCATGTCCTAACCCTGATCCTCTTGTCCCACAGATATCCAGAACCCTCTAAGAAGTTCCTGCTTTTAAATAAATCCCAGAATGTGGTTAGAGACATGACCCTGCCGTGTACCAGCTGAGAGACTCTAAATCCAGTGACAAGTCTG |
| G1 | TC15P1_pool1_7 | GAGATCATGTCCTAACCCTGATCCTCTTGTCCCACAGATATCCAGAACCCTCTAAGAAGTTCCTGCTTTAAAAAAAATCCGAGAATGTGGTTAGAGACATGACCCTGCCGTGTACCAGCTGAGAGACTCTAAATCCAGTGACAAGTCTG |
| H1 | TC15P1_pool1_8 | GAGATCATGTCCTAACCCTGATCCTCTTGTCCCACAGATATCCAGAACCCTCTAAGAAGTTCCTGCTTATTATTTAATCCTTGAATGTGGTTAGAGACATGACCCTGCCGTGTACCAGCTGAGAGACTCTAAATCCAGTGACAAGTCTG |
| A2 | TC15P1_pool1_9 | GAGATCATGTCCTAACCCTGATCCTCTTGTCCCACAGATATCCAGAACCCTCTAAGAAGTTCCTGCTTTATTTAAAATCGATGAATGTGGTTAGAGACATGACCCTGCCGTGTACCAGCTGAGAGACTCTAAATCCAGTGACAAGTCTG |
| B2 | TC15P1_pool1_10 | GAGATCATGTCCTAACCCTGATCCTCTTGTCCCACAGATATCCAGAACCCTCTAAGAAGTTCCTGCTAAAATAATAATCGCAGAATGTGGTTAGAGACATGACCCTGCCGTGTACCAGCTGAGAGACTCTAAATCCAGTGACAAGTCTG |
| C2 | TC15P1_pool1_11 | GAGATCATGTCCTAACCCTGATCCTCTTGTCCCACAGATATCCAGAACCCTCTAAGAAGTTCCTGCTATATTAAAAATCGGTGAATGTGGTTAGAGACATGACCCTGCCGTGTACCAGCTGAGAGACTCTAAATCCAGTGACAAGTCTG |
| D2 | TC15P1_pool1_12 | GAGATCATGTCCTAACCCTGATCCTCTTGTCCCACAGATATCCAGAACCCTCTAAGAAGTTCCTGCTATTAATATAATCGTAGAATGTGGTTAGAGACATGACCCTGCCGTGTACCAGCTGAGAGACTCTAAATCCAGTGACAAGTCTG |
| E2 | TC15P1_pool1_13 | GAGATCATGTCCTAACCCTGATCCTCTTGTCCCACAGATATCCAGAACCCTCTAAGAAGTTCCTGCTATTATATTAATCTATGAATGTGGTTAGAGACATGACCCTGCCGTGTACCAGCTGAGAGACTCTAAATCCAGTGACAAGTCTG |
| F2 | TC15P1_pool1_14 | GAGATCATGTCCTAACCCTGATCCTCTTGTCCCACAGATATCCAGAACCCTCTAAGAAGTTCCTGCTATATATTTAATCTCTGAATGTGGTTAGAGACATGACCCTGCCGTGTACCAGCTGAGAGACTCTAAATCCAGTGACAAGTCTG |
| G2 | TC15P1_pool1_15 | GAGATCATGTCCTAACCCTGATCCTCTTGTCCCACAGATATCCAGAACCCTCTAAGAAGTTCCTGCTAAAATATAAATCTGTGAATGTGGTTAGAGACATGACCCTGCCGTGTACCAGCTGAGAGACTCTAAATCCAGTGACAAGTCTG |
| H2 | TC15P1_pool1_16 | GAGATCATGTCCTAACCCTGATCCTCTTGTCCCACAGATATCCAGAACCCTCTAAGAAGTTCCTGCTTATAATAAAATCTTTGAATGTGGTTAGAGACATGACCCTGCCGTGTACCAGCTGAGAGACTCTAAATCCAGTGACAAGTCTG |
| A3 | TC15P1_pool1_17 | GAGATCATGTCCTAACCCTGATCCTCTTGTCCCACAGATATCCAGAACCCTCTAAGAAGTTCCTGCTTAATAAATACTCAATGAATGTGGTTAGAGACATGACCCTGCCGTGTACCAGCTGAGAGACTCTAAATCCAGTGACAAGTCTG |
| B3 | TC15P1_pool1_18 | GAGATCATGTCCTAACCCTGATCCTCTTGTCCCACAGATATCCAGAACCCTCTAAGAAGTTCCTGCTAATAATAAACTCACAGAATGTGGTTAGAGACATGACCCTGCCGTGTACCAGCTGAGAGACTCTAAATCCAGTGACAAGTCTG |
| C3 | TC15P1_pool1_19 | GAGATCATGTCCTAACCCTGATCCTCTTGTCCCACAGATATCCAGAACCCTCTAAGAAGTTCCTGCTATAATAATACTCAGTGAATGTGGTTAGAGACATGACCCTGCCGTGTACCAGCTGAGAGACTCTAAATCCAGTGACAAGTCTG |
| D3 | TC15P1_pool1_20 | GAGATCATGTCCTAACCCTGATCCTCTTGTCCCACAGATATCCAGAACCCTCTAAGAAGTTCCTGCTTTATTAAAACTCATAGAATGTGGTTAGAGACATGACCCTGCCGTGTACCAGCTGAGAGACTCTAAATCCAGTGACAAGTCTG |
| E3 | TC15P1_pool1_21 | GAGATCATGTCCTAACCCTGATCCTCTTGTCCCACAGATATCCAGAACCCTCTAAGAAGTTCCTGCTAATTATTTACTCCAAGAATGTGGTTAGAGACATGACCCTGCCGTGTACCAGCTGAGAGACTCTAAATCCAGTGACAAGTCTG |
| F3 | TC15P1_pool1_22 | GAGATCATGTCCTAACCCTGATCCTCTTGTCCCACAGATATCCAGAACCCTCTAAGAAGTTCCTGCTAAATAATTACTCCCAGAATGTGGTTAGAGACATGACCCTGCCGTGTACCAGCTGAGAGACTCTAAATCCAGTGACAAGTCTG |
| G3 | TC15P1_pool1_23 | GAGATCATGTCCTAACCCTGATCCTCTTGTCCCACAGATATCCAGAACCCTCTAAGAAGTTCCTGCTTATTTAATACTCCGTGAATGTGGTTAGAGACATGACCCTGCCGTGTACCAGCTGAGAGACTCTAAATCCAGTGACAAGTCTG |
| H3 | TC15P1_pool1_24 | GAGATCATGTCCTAACCCTGATCCTCTTGTCCCACAGATATCCAGAACCCTCTAAGAAGTTCCTGCTTTAATTTAACTCCTAGAATGTGGTTAGAGACATGACCCTGCCGTGTACCAGCTGAGAGACTCTAAATCCAGTGACAAGTCTG |
| A4 | TC15P1_pool1_25 | GAGATCATGTCCTAACCCTGATCCTCTTGTCCCACAGATATCCAGAACCCTCTAAGAAGTTCCTGCTAATTTAAAACTCGATGAATGTGGTTAGAGACATGACCCTGCCGTGTACCAGCTGAGAGACTCTAAATCCAGTGACAAGTCTG |
| B4 | TC15P1_pool1_26 | GAGATCATGTCCTAACCCTGATCCTCTTGTCCCACAGATATCCAGAACCCTCTAAGAAGTTCCTGCTTATTTATAACTCGCTGAATGTGGTTAGAGACATGACCCTGCCGTGTACCAGCTGAGAGACTCTAAATCCAGTGACAAGTCTG |
| C4 | TC15P1_pool1_27 | GAGATCATGTCCTAACCCTGATCCTCTTGTCCCACAGATATCCAGAACCCTCTAAGAAGTTCCTGCTTTTAATTAACTCGGTGAATGTGGTTAGAGACATGACCCTGCCGTGTACCAGCTGAGAGACTCTAAATCCAGTGACAAGTCTG |
| D4 | TC15P1_pool1_28 | GAGATCATGTCCTAACCCTGATCCTCTTGTCCCACAGATATCCAGAACCCTCTAAGAAGTTCCTGCTTTAATATAACTCGTTGAATGTGGTTAGAGACATGACCCTGCCGTGTACCAGCTGAGAGACTCTAAATCCAGTGACAAGTCTG |
| E4 | TC15P1_pool1_29 | GAGATCATGTCCTAACCCTGATCCTCTTGTCCCACAGATATCCAGAACCCTCTAAGAAGTTCCTGCTAAATTTAAACTCTAAGAATGTGGTTAGAGACATGACCCTGCCGTGTACCAGCTGAGAGACTCTAAATCCAGTGACAAGTCTG |
| F4 | TC15P1_pool1_30 | GAGATCATGTCCTAACCCTGATCCTCTTGTCCCACAGATATCCAGAACCCTCTAAGAAGTTCCTGCTATAAAAAAACTCTCTGAATGTGGTTAGAGACATGACCCTGCCGTGTACCAGCTGAGAGACTCTAAATCCAGTGACAAGTCTG |
| G4 | TC15P1_pool1_31 | GAGATCATGTCCTAACCCTGATCCTCTTGTCCCACAGATATCCAGAACCCTCTAAGAAGTTCCTGCTTTAATAATACTCTGAGAATGTGGTTAGAGACATGACCCTGCCGTGTACCAGCTGAGAGACTCTAAATCCAGTGACAAGTCTG |
| H4 | TC15P1_pool1_32 | GAGATCATGTCCTAACCCTGATCCTCTTGTCCCACAGATATCCAGAACCCTCTAAGAAGTTCCTGCTATTAATAAACTCTTTGAATGTGGTTAGAGACATGACCCTGCCGTGTACCAGCTGAGAGACTCTAAATCCAGTGACAAGTCTG |
| A5 | TC15P1_pool1_33 | GAGATCATGTCCTAACCCTGATCCTCTTGTCCCACAGATATCCAGAACCCTCTAAGAAGTTCCTGCTATATTAATAGTCAAAGAATGTGGTTAGAGACATGACCCTGCCGTGTACCAGCTGAGAGACTCTAAATCCAGTGACAAGTCTG |
| B5 | TC15P1_pool1_34 | GAGATCATGTCCTAACCCTGATCCTCTTGTCCCACAGATATCCAGAACCCTCTAAGAAGTTCCTGCTTTATAATTAGTCACTGAATGTGGTTAGAGACATGACCCTGCCGTGTACCAGCTGAGAGACTCTAAATCCAGTGACAAGTCTG |
| C5 | TC15P1_pool1_35 | GAGATCATGTCCTAACCCTGATCCTCTTGTCCCACAGATATCCAGAACCCTCTAAGAAGTTCCTGCTTATATATTAGTCAGTGAATGTGGTTAGAGACATGACCCTGCCGTGTACCAGCTGAGAGACTCTAAATCCAGTGACAAGTCTG |
| D5 | TC15P1_pool1_36 | GAGATCATGTCCTAACCCTGATCCTCTTGTCCCACAGATATCCAGAACCCTCTAAGAAGTTCCTGCTTATATTTTAGTCATAGAATGTGGTTAGAGACATGACCCTGCCGTGTACCAGCTGAGAGACTCTAAATCCAGTGACAAGTCTG |
| E5 | TC15P1_pool1_37 | GAGATCATGTCCTAACCCTGATCCTCTTGTCCCACAGATATCCAGAACCCTCTAAGAAGTTCCTGCTTAATATTAAGTCCATGAATGTGGTTAGAGACATGACCCTGCCGTGTACCAGCTGAGAGACTCTAAATCCAGTGACAAGTCTG |
| F5 | TC15P1_pool1_38 | GAGATCATGTCCTAACCCTGATCCTCTTGTCCCACAGATATCCAGAACCCTCTAAGAAGTTCCTGCTTAAAATTTAGTCCCAGAATGTGGTTAGAGACATGACCCTGCCGTGTACCAGCTGAGAGACTCTAAATCCAGTGACAAGTCTG |
| G5 | TC15P1_pool1_39 | GAGATCATGTCCTAACCCTGATCCTCTTGTCCCACAGATATCCAGAACCCTCTAAGAAGTTCCTGCTATATAATAAGTCCGTGAATGTGGTTAGAGACATGACCCTGCCGTGTACCAGCTGAGAGACTCTAAATCCAGTGACAAGTCTG |
| H5 | TC15P1_pool1_40 | GAGATCATGTCCTAACCCTGATCCTCTTGTCCCACAGATATCCAGAACCCTCTAAGAAGTTCCTGCTAATTAAAAAGTCCTAGAATGTGGTTAGAGACATGACCCTGCCGTGTACCAGCTGAGAGACTCTAAATCCAGTGACAAGTCTG |
| A6 | TC15P1_pool1_41 | GAGATCATGTCCTAACCCTGATCCTCTTGTCCCACAGATATCCAGAACCCTCTAAGAAGTTCCTGCTAATAAAATAGTCGATGAATGTGGTTAGAGACATGACCCTGCCGTGTACCAGCTGAGAGACTCTAAATCCAGTGACAAGTCTG |
| B6 | TC15P1_pool1_42 | GAGATCATGTCCTAACCCTGATCCTCTTGTCCCACAGATATCCAGAACCCTCTAAGAAGTTCCTGCTAAAAATAAAGTCGCTGAATGTGGTTAGAGACATGACCCTGCCGTGTACCAGCTGAGAGACTCTAAATCCAGTGACAAGTCTG |
| C6 | TC15P1_pool1_43 | GAGATCATGTCCTAACCCTGATCCTCTTGTCCCACAGATATCCAGAACCCTCTAAGAAGTTCCTGCTATTTTTAAAGTCGGTGAATGTGGTTAGAGACATGACCCTGCCGTGTACCAGCTGAGAGACTCTAAATCCAGTGACAAGTCTG |
| D6 | TC15P1_pool1_44 | GAGATCATGTCCTAACCCTGATCCTCTTGTCCCACAGATATCCAGAACCCTCTAAGAAGTTCCTGCTTTTTTAAAAGTCGTTGAATGTGGTTAGAGACATGACCCTGCCGTGTACCAGCTGAGAGACTCTAAATCCAGTGACAAGTCTG |
| E6 | TC15P1_pool1_45 | GAGATCATGTCCTAACCCTGATCCTCTTGTCCCACAGATATCCAGAACCCTCTAAGAAGTTCCTGCTATAATTATAGTCTAAGAATGTGGTTAGAGACATGACCCTGCCGTGTACCAGCTGAGAGACTCTAAATCCAGTGACAAGTCTG |
| F6 | TC15P1_pool1_46 | GAGATCATGTCCTAACCCTGATCCTCTTGTCCCACAGATATCCAGAACCCTCTAAGAAGTTCCTGCTAATAAATAAGTCTCAGAATGTGGTTAGAGACATGACCCTGCCGTGTACCAGCTGAGAGACTCTAAATCCAGTGACAAGTCTG |
| G6 | TC15P1_pool1_47 | GAGATCATGTCCTAACCCTGATCCTCTTGTCCCACAGATATCCAGAACCCTCTAAGAAGTTCCTGCTTATATAATAGTCTGAGAATGTGGTTAGAGACATGACCCTGCCGTGTACCAGCTGAGAGACTCTAAATCCAGTGACAAGTCTG |
| H6 | TC15P1_pool1_48 | GAGATCATGTCCTAACCCTGATCCTCTTGTCCCACAGATATCCAGAACCCTCTAAGAAGTTCCTGCTTAAAAAAAAGTCTTTGAATGTGGTTAGAGACATGACCCTGCCGTGTACCAGCTGAGAGACTCTAAATCCAGTGACAAGTCTG |
| A7 | TC15P1_pool1_49 | GAGATCATGTCCTAACCCTGATCCTCTTGTCCCACAGATATCCAGAACCCTCTAAGAAGTTCCTGCTATTTAATTATTCAAAGAATGTGGTTAGAGACATGACCCTGCCGTGTACCAGCTGAGAGACTCTAAATCCAGTGACAAGTCTG |
| B7 | TC15P1_pool1_50 | GAGATCATGTCCTAACCCTGATCCTCTTGTCCCACAGATATCCAGAACCCTCTAAGAAGTTCCTGCTTTATAATAATTCACAGAATGTGGTTAGAGACATGACCCTGCCGTGTACCAGCTGAGAGACTCTAAATCCAGTGACAAGTCTG |
| C7 | TC15P1_pool1_51 | GAGATCATGTCCTAACCCTGATCCTCTTGTCCCACAGATATCCAGAACCCTCTAAGAAGTTCCTGCTTATATATAATTCAGAGAATGTGGTTAGAGACATGACCCTGCCGTGTACCAGCTGAGAGACTCTAAATCCAGTGACAAGTCTG |
| D7 | TC15P1_pool1_52 | GAGATCATGTCCTAACCCTGATCCTCTTGTCCCACAGATATCCAGAACCCTCTAAGAAGTTCCTGCTAAAAAAAAATTCATAGAATGTGGTTAGAGACATGACCCTGCCGTGTACCAGCTGAGAGACTCTAAATCCAGTGACAAGTCTG |
| E7 | TC15P1_pool1_53 | GAGATCATGTCCTAACCCTGATCCTCTTGTCCCACAGATATCCAGAACCCTCTAAGAAGTTCCTGCTTATTTTAAATTCCAAGAATGTGGTTAGAGACATGACCCTGCCGTGTACCAGCTGAGAGACTCTAAATCCAGTGACAAGTCTG |
| F7 | TC15P1_pool1_54 | GAGATCATGTCCTAACCCTGATCCTCTTGTCCCACAGATATCCAGAACCCTCTAAGAAGTTCCTGCTAATTTTTAATTCCCAGAATGTGGTTAGAGACATGACCCTGCCGTGTACCAGCTGAGAGACTCTAAATCCAGTGACAAGTCTG |
| G7 | TC15P1_pool1_55 | GAGATCATGTCCTAACCCTGATCCTCTTGTCCCACAGATATCCAGAACCCTCTAAGAAGTTCCTGCTAAATTATAATTCCGAGAATGTGGTTAGAGACATGACCCTGCCGTGTACCAGCTGAGAGACTCTAAATCCAGTGACAAGTCTG |
| H7 | TC15P1_pool1_56 | GAGATCATGTCCTAACCCTGATCCTCTTGTCCCACAGATATCCAGAACCCTCTAAGAAGTTCCTGCTTTTAAATTATTCCTTGAATGTGGTTAGAGACATGACCCTGCCGTGTACCAGCTGAGAGACTCTAAATCCAGTGACAAGTCTG |
| A8 | TC15P1_pool1_57 | GAGATCATGTCCTAACCCTGATCCTCTTGTCCCACAGATATCCAGAACCCTCTAAGAAGTTCCTGCTTTTAATTTATTCGAAGAATGTGGTTAGAGACATGACCCTGCCGTGTACCAGCTGAGAGACTCTAAATCCAGTGACAAGTCTG |
| B8 | TC15P1_pool1_58 | GAGATCATGTCCTAACCCTGATCCTCTTGTCCCACAGATATCCAGAACCCTCTAAGAAGTTCCTGCTATAATTAAATTCGCTGAATGTGGTTAGAGACATGACCCTGCCGTGTACCAGCTGAGAGACTCTAAATCCAGTGACAAGTCTG |
| C8 | TC15P1_pool1_59 | GAGATCATGTCCTAACCCTGATCCTCTTGTCCCACAGATATCCAGAACCCTCTAAGAAGTTCCTGCTTTAAAATTATTCGGAGAATGTGGTTAGAGACATGACCCTGCCGTGTACCAGCTGAGAGACTCTAAATCCAGTGACAAGTCTG |
| D8 | TC15P1_pool1_60 | GAGATCATGTCCTAACCCTGATCCTCTTGTCCCACAGATATCCAGAACCCTCTAAGAAGTTCCTGCTTTATTTTTATTCGTTGAATGTGGTTAGAGACATGACCCTGCCGTGTACCAGCTGAGAGACTCTAAATCCAGTGACAAGTCTG |
| E8 | TC15P1_pool1_61 | GAGATCATGTCCTAACCCTGATCCTCTTGTCCCACAGATATCCAGAACCCTCTAAGAAGTTCCTGCTTAATTAAAATTCTATGAATGTGGTTAGAGACATGACCCTGCCGTGTACCAGCTGAGAGACTCTAAATCCAGTGACAAGTCTG |
| F8 | TC15P1_pool1_62 | GAGATCATGTCCTAACCCTGATCCTCTTGTCCCACAGATATCCAGAACCCTCTAAGAAGTTCCTGCTTATAAAAAATTCTCAGAATGTGGTTAGAGACATGACCCTGCCGTGTACCAGCTGAGAGACTCTAAATCCAGTGACAAGTCTG |
| G8 | TC15P1_pool1_63 | GAGATCATGTCCTAACCCTGATCCTCTTGTCCCACAGATATCCAGAACCCTCTAAGAAGTTCCTGCTTATATTTAATTCTGTGAATGTGGTTAGAGACATGACCCTGCCGTGTACCAGCTGAGAGACTCTAAATCCAGTGACAAGTCTG |
| H8 | TC15P1_pool1_64 | GAGATCATGTCCTAACCCTGATCCTCTTGTCCCACAGATATCCAGAACCCTCTAAGAAGTTCCTGCTTAAATAATATTCTTTGAATGTGGTTAGAGACATGACCCTGCCGTGTACCAGCTGAGAGACTCTAAATCCAGTGACAAGTCTG |
| A9 | TC15P1_pool1_65 | GAGATCATGTCCTAACCCTGATCCTCTTGTCCCACAGATATCCAGAACCCTCTAAGAAGTTCCTGCTAATTTTATCATCAAAGAATGTGGTTAGAGACATGACCCTGCCGTGTACCAGCTGAGAGACTCTAAATCCAGTGACAAGTCTG |
| B9 | TC15P1_pool1_66 | GAGATCATGTCCTAACCCTGATCCTCTTGTCCCACAGATATCCAGAACCCTCTAAGAAGTTCCTGCTATTTATTACATCACTGAATGTGGTTAGAGACATGACCCTGCCGTGTACCAGCTGAGAGACTCTAAATCCAGTGACAAGTCTG |
| C9 | TC15P1_pool1_67 | GAGATCATGTCCTAACCCTGATCCTCTTGTCCCACAGATATCCAGAACCCTCTAAGAAGTTCCTGCTAATTTTTTCATCAGTGAATGTGGTTAGAGACATGACCCTGCCGTGTACCAGCTGAGAGACTCTAAATCCAGTGACAAGTCTG |
| D9 | TC15P1_pool1_68 | GAGATCATGTCCTAACCCTGATCCTCTTGTCCCACAGATATCCAGAACCCTCTAAGAAGTTCCTGCTTTTTAATACATCATTGAATGTGGTTAGAGACATGACCCTGCCGTGTACCAGCTGAGAGACTCTAAATCCAGTGACAAGTCTG |
| E9 | TC15P1_pool1_69 | GAGATCATGTCCTAACCCTGATCCTCTTGTCCCACAGATATCCAGAACCCTCTAAGAAGTTCCTGCTAAATTTTACATCCATGAATGTGGTTAGAGACATGACCCTGCCGTGTACCAGCTGAGAGACTCTAAATCCAGTGACAAGTCTG |
| F9 | TC15P1_pool1_70 | GAGATCATGTCCTAACCCTGATCCTCTTGTCCCACAGATATCCAGAACCCTCTAAGAAGTTCCTGCTTTTTAAATCATCCCTGAATGTGGTTAGAGACATGACCCTGCCGTGTACCAGCTGAGAGACTCTAAATCCAGTGACAAGTCTG |
| G9 | TC15P1_pool1_71 | GAGATCATGTCCTAACCCTGATCCTCTTGTCCCACAGATATCCAGAACCCTCTAAGAAGTTCCTGCTATATTTTTCATCCGAGAATGTGGTTAGAGACATGACCCTGCCGTGTACCAGCTGAGAGACTCTAAATCCAGTGACAAGTCTG |
| H9 | TC15P1_pool1_72 | GAGATCATGTCCTAACCCTGATCCTCTTGTCCCACAGATATCCAGAACCCTCTAAGAAGTTCCTGCTTATAAATTCATCCTAGAATGTGGTTAGAGACATGACCCTGCCGTGTACCAGCTGAGAGACTCTAAATCCAGTGACAAGTCTG |
| A10 | TC15P1_pool1_73 | GAGATCATGTCCTAACCCTGATCCTCTTGTCCCACAGATATCCAGAACCCTCTAAGAAGTTCCTGCTAATATTAACATCGATGAATGTGGTTAGAGACATGACCCTGCCGTGTACCAGCTGAGAGACTCTAAATCCAGTGACAAGTCTG |
| B10 | TC15P1_pool1_74 | GAGATCATGTCCTAACCCTGATCCTCTTGTCCCACAGATATCCAGAACCCTCTAAGAAGTTCCTGCTAAATTTATCATCGCTGAATGTGGTTAGAGACATGACCCTGCCGTGTACCAGCTGAGAGACTCTAAATCCAGTGACAAGTCTG |
| C10 | TC15P1_pool1_75 | GAGATCATGTCCTAACCCTGATCCTCTTGTCCCACAGATATCCAGAACCCTCTAAGAAGTTCCTGCTTTATAAAACATCGGTGAATGTGGTTAGAGACATGACCCTGCCGTGTACCAGCTGAGAGACTCTAAATCCAGTGACAAGTCTG |
| D10 | TC15P1_pool1_76 | GAGATCATGTCCTAACCCTGATCCTCTTGTCCCACAGATATCCAGAACCCTCTAAGAAGTTCCTGCTTAAAAATACATCGTAGAATGTGGTTAGAGACATGACCCTGCCGTGTACCAGCTGAGAGACTCTAAATCCAGTGACAAGTCTG |
| E10 | TC15P1_pool1_77 | GAGATCATGTCCTAACCCTGATCCTCTTGTCCCACAGATATCCAGAACCCTCTAAGAAGTTCCTGCTATTAATTTCATCTATGAATGTGGTTAGAGACATGACCCTGCCGTGTACCAGCTGAGAGACTCTAAATCCAGTGACAAGTCTG |
| F10 | TC15P1_pool1_78 | GAGATCATGTCCTAACCCTGATCCTCTTGTCCCACAGATATCCAGAACCCTCTAAGAAGTTCCTGCTTATATAAACATCTCTGAATGTGGTTAGAGACATGACCCTGCCGTGTACCAGCTGAGAGACTCTAAATCCAGTGACAAGTCTG |
| G10 | TC15P1_pool1_79 | GAGATCATGTCCTAACCCTGATCCTCTTGTCCCACAGATATCCAGAACCCTCTAAGAAGTTCCTGCTATAAATTACATCTGTGAATGTGGTTAGAGACATGACCCTGCCGTGTACCAGCTGAGAGACTCTAAATCCAGTGACAAGTCTG |
| H10 | TC15P1_pool1_80 | GAGATCATGTCCTAACCCTGATCCTCTTGTCCCACAGATATCCAGAACCCTCTAAGAAGTTCCTGCTTTTATTATCATCTTTGAATGTGGTTAGAGACATGACCCTGCCGTGTACCAGCTGAGAGACTCTAAATCCAGTGACAAGTCTG |
| A11 | TC15P1_pool1_81 | GAGATCATGTCCTAACCCTGATCCTCTTGTCCCACAGATATCCAGAACCCTCTAAGAAGTTCCTGCTATAAATTTCCTCAAAGAATGTGGTTAGAGACATGACCCTGCCGTGTACCAGCTGAGAGACTCTAAATCCAGTGACAAGTCTG |
| B11 | TC15P1_pool1_82 | GAGATCATGTCCTAACCCTGATCCTCTTGTCCCACAGATATCCAGAACCCTCTAAGAAGTTCCTGCTATTATTTACCTCACTGAATGTGGTTAGAGACATGACCCTGCCGTGTACCAGCTGAGAGACTCTAAATCCAGTGACAAGTCTG |
| C11 | TC15P1_pool1_83 | GAGATCATGTCCTAACCCTGATCCTCTTGTCCCACAGATATCCAGAACCCTCTAAGAAGTTCCTGCTAAATAAAACCTCAGTGAATGTGGTTAGAGACATGACCCTGCCGTGTACCAGCTGAGAGACTCTAAATCCAGTGACAAGTCTG |
| D11 | TC15P1_pool1_84 | GAGATCATGTCCTAACCCTGATCCTCTTGTCCCACAGATATCCAGAACCCTCTAAGAAGTTCCTGCTTAATTAATCCTCATAGAATGTGGTTAGAGACATGACCCTGCCGTGTACCAGCTGAGAGACTCTAAATCCAGTGACAAGTCTG |
| E11 | TC15P1_pool1_85 | GAGATCATGTCCTAACCCTGATCCTCTTGTCCCACAGATATCCAGAACCCTCTAAGAAGTTCCTGCTAATTATAACCTCCATGAATGTGGTTAGAGACATGACCCTGCCGTGTACCAGCTGAGAGACTCTAAATCCAGTGACAAGTCTG |
| F11 | TC15P1_pool1_86 | GAGATCATGTCCTAACCCTGATCCTCTTGTCCCACAGATATCCAGAACCCTCTAAGAAGTTCCTGCTATATTATTCCTCCCTGAATGTGGTTAGAGACATGACCCTGCCGTGTACCAGCTGAGAGACTCTAAATCCAGTGACAAGTCTG |
| G11 | TC15P1_pool1_87 | GAGATCATGTCCTAACCCTGATCCTCTTGTCCCACAGATATCCAGAACCCTCTAAGAAGTTCCTGCTTTATAAATCCTCCGAGAATGTGGTTAGAGACATGACCCTGCCGTGTACCAGCTGAGAGACTCTAAATCCAGTGACAAGTCTG |
| H11 | TC15P1_pool1_88 | GAGATCATGTCCTAACCCTGATCCTCTTGTCCCACAGATATCCAGAACCCTCTAAGAAGTTCCTGCTTTTTATATCCTCCTAGAATGTGGTTAGAGACATGACCCTGCCGTGTACCAGCTGAGAGACTCTAAATCCAGTGACAAGTCTG |
| A12 | TC15P1_pool1_89 | GAGATCATGTCCTAACCCTGATCCTCTTGTCCCACAGATATCCAGAACCCTCTAAGAAGTTCCTGCTAAAATTTTCCTCGAAGAATGTGGTTAGAGACATGACCCTGCCGTGTACCAGCTGAGAGACTCTAAATCCAGTGACAAGTCTG |
| B12 | TC15P1_pool1_90 | GAGATCATGTCCTAACCCTGATCCTCTTGTCCCACAGATATCCAGAACCCTCTAAGAAGTTCCTGCTTATTAAAACCTCGCTGAATGTGGTTAGAGACATGACCCTGCCGTGTACCAGCTGAGAGACTCTAAATCCAGTGACAAGTCTG |
| C12 | TC15P1_pool1_91 | GAGATCATGTCCTAACCCTGATCCTCTTGTCCCACAGATATCCAGAACCCTCTAAGAAGTTCCTGCTTAAATTAACCTCGGAGAATGTGGTTAGAGACATGACCCTGCCGTGTACCAGCTGAGAGACTCTAAATCCAGTGACAAGTCTG |
| D12 | TC15P1_pool1_92 | GAGATCATGTCCTAACCCTGATCCTCTTGTCCCACAGATATCCAGAACCCTCTAAGAAGTTCCTGCTTAAATATTCCTCGTAGAATGTGGTTAGAGACATGACCCTGCCGTGTACCAGCTGAGAGACTCTAAATCCAGTGACAAGTCTG |
| E12 | TC15P1_pool1_93 | GAGATCATGTCCTAACCCTGATCCTCTTGTCCCACAGATATCCAGAACCCTCTAAGAAGTTCCTGCTAATAATTACCTCTATGAATGTGGTTAGAGACATGACCCTGCCGTGTACCAGCTGAGAGACTCTAAATCCAGTGACAAGTCTG |
| F12 | TC15P1_pool1_94 | GAGATCATGTCCTAACCCTGATCCTCTTGTCCCACAGATATCCAGAACCCTCTAAGAAGTTCCTGCTATATAAATCCTCTCTGAATGTGGTTAGAGACATGACCCTGCCGTGTACCAGCTGAGAGACTCTAAATCCAGTGACAAGTCTG |
| G12 | TC15P1_pool1_95 | GAGATCATGTCCTAACCCTGATCCTCTTGTCCCACAGATATCCAGAACCCTCTAAGAAGTTCCTGCTTTAAATATCCTCTGTGAATGTGGTTAGAGACATGACCCTGCCGTGTACCAGCTGAGAGACTCTAAATCCAGTGACAAGTCTG |
| H12 | TC15P1_pool1_96 | GAGATCATGTCCTAACCCTGATCCTCTTGTCCCACAGATATCCAGAACCCTCTAAGAAGTTCCTGCTAAAAATATCCTCTTAGAATGTGGTTAGAGACATGACCCTGCCGTGTACCAGCTGAGAGACTCTAAATCCAGTGACAAGTCTG |
| A1 | TC15P2_pool1_97 | GAGATCATGTCCTAACCCTGATCCTCTTGTCCCACAGATATCCAGAACCCTCTAAGAAGTTCCTGCTATATATATCGTCAAAGAATGTGGTTAGAGACATGACCCTGCCGTGTACCAGCTGAGAGACTCTAAATCCAGTGACAAGTCTG |
| B1 | TC15P2_pool1_98 | GAGATCATGTCCTAACCCTGATCCTCTTGTCCCACAGATATCCAGAACCCTCTAAGAAGTTCCTGCTTTATTAATCGTCACTGAATGTGGTTAGAGACATGACCCTGCCGTGTACCAGCTGAGAGACTCTAAATCCAGTGACAAGTCTG |
| C1 | TC15P2_pool1_99 | GAGATCATGTCCTAACCCTGATCCTCTTGTCCCACAGATATCCAGAACCCTCTAAGAAGTTCCTGCTATTATAAACGTCAGAGAATGTGGTTAGAGACATGACCCTGCCGTGTACCAGCTGAGAGACTCTAAATCCAGTGACAAGTCTG |
| D1 | TC15P2_pool1_100 | GAGATCATGTCCTAACCCTGATCCTCTTGTCCCACAGATATCCAGAACCCTCTAAGAAGTTCCTGCTTTTAAAAACGTCATTGAATGTGGTTAGAGACATGACCCTGCCGTGTACCAGCTGAGAGACTCTAAATCCAGTGACAAGTCTG |
| E1 | TC15P2_pool1_101 | GAGATCATGTCCTAACCCTGATCCTCTTGTCCCACAGATATCCAGAACCCTCTAAGAAGTTCCTGCTTATAAATACGTCCATGAATGTGGTTAGAGACATGACCCTGCCGTGTACCAGCTGAGAGACTCTAAATCCAGTGACAAGTCTG |
| F1 | TC15P2_pool1_102 | GAGATCATGTCCTAACCCTGATCCTCTTGTCCCACAGATATCCAGAACCCTCTAAGAAGTTCCTGCTTTTAATAACGTCCCAGAATGTGGTTAGAGACATGACCCTGCCGTGTACCAGCTGAGAGACTCTAAATCCAGTGACAAGTCTG |
| G1 | TC15P2_pool1_103 | GAGATCATGTCCTAACCCTGATCCTCTTGTCCCACAGATATCCAGAACCCTCTAAGAAGTTCCTGCTATAATTTTCGTCCGTGAATGTGGTTAGAGACATGACCCTGCCGTGTACCAGCTGAGAGACTCTAAATCCAGTGACAAGTCTG |
| H1 | TC15P2_pool1_104 | GAGATCATGTCCTAACCCTGATCCTCTTGTCCCACAGATATCCAGAACCCTCTAAGAAGTTCCTGCTTTTTTATTCGTCCTAGAATGTGGTTAGAGACATGACCCTGCCGTGTACCAGCTGAGAGACTCTAAATCCAGTGACAAGTCTG |
| A2 | TC15P2_pool1_105 | GAGATCATGTCCTAACCCTGATCCTCTTGTCCCACAGATATCCAGAACCCTCTAAGAAGTTCCTGCTTAATTATTCGTCGATGAATGTGGTTAGAGACATGACCCTGCCGTGTACCAGCTGAGAGACTCTAAATCCAGTGACAAGTCTG |
| B2 | TC15P2_pool1_106 | GAGATCATGTCCTAACCCTGATCCTCTTGTCCCACAGATATCCAGAACCCTCTAAGAAGTTCCTGCTATAAAAATCGTCGCAGAATGTGGTTAGAGACATGACCCTGCCGTGTACCAGCTGAGAGACTCTAAATCCAGTGACAAGTCTG |
| C2 | TC15P2_pool1_107 | GAGATCATGTCCTAACCCTGATCCTCTTGTCCCACAGATATCCAGAACCCTCTAAGAAGTTCCTGCTTATAATATCGTCGGAGAATGTGGTTAGAGACATGACCCTGCCGTGTACCAGCTGAGAGACTCTAAATCCAGTGACAAGTCTG |
| D2 | TC15P2_pool1_108 | GAGATCATGTCCTAACCCTGATCCTCTTGTCCCACAGATATCCAGAACCCTCTAAGAAGTTCCTGCTTAATATATCGTCGTAGAATGTGGTTAGAGACATGACCCTGCCGTGTACCAGCTGAGAGACTCTAAATCCAGTGACAAGTCTG |
| E2 | TC15P2_pool1_109 | GAGATCATGTCCTAACCCTGATCCTCTTGTCCCACAGATATCCAGAACCCTCTAAGAAGTTCCTGCTTAATTTTTCGTCTAAGAATGTGGTTAGAGACATGACCCTGCCGTGTACCAGCTGAGAGACTCTAAATCCAGTGACAAGTCTG |
| F2 | TC15P2_pool1_110 | GAGATCATGTCCTAACCCTGATCCTCTTGTCCCACAGATATCCAGAACCCTCTAAGAAGTTCCTGCTATTTAAAACGTCTCTGAATGTGGTTAGAGACATGACCCTGCCGTGTACCAGCTGAGAGACTCTAAATCCAGTGACAAGTCTG |
| G2 | TC15P2_pool1_111 | GAGATCATGTCCTAACCCTGATCCTCTTGTCCCACAGATATCCAGAACCCTCTAAGAAGTTCCTGCTATATAAAACGTCTGAGAATGTGGTTAGAGACATGACCCTGCCGTGTACCAGCTGAGAGACTCTAAATCCAGTGACAAGTCTG |
| H2 | TC15P2_pool1_112 | GAGATCATGTCCTAACCCTGATCCTCTTGTCCCACAGATATCCAGAACCCTCTAAGAAGTTCCTGCTATAATATTCGTCTTAGAATGTGGTTAGAGACATGACCCTGCCGTGTACCAGCTGAGAGACTCTAAATCCAGTGACAAGTCTG |
| A3 | TC15P2_pool1_113 | GAGATCATGTCCTAACCCTGATCCTCTTGTCCCACAGATATCCAGAACCCTCTAAGAAGTTCCTGCTATTATTTTCTTCAAAGAATGTGGTTAGAGACATGACCCTGCCGTGTACCAGCTGAGAGACTCTAAATCCAGTGACAAGTCTG |
| B3 | TC15P2_pool1_114 | GAGATCATGTCCTAACCCTGATCCTCTTGTCCCACAGATATCCAGAACCCTCTAAGAAGTTCCTGCTAATTAATACTTCACTGAATGTGGTTAGAGACATGACCCTGCCGTGTACCAGCTGAGAGACTCTAAATCCAGTGACAAGTCTG |
| C3 | TC15P2_pool1_115 | GAGATCATGTCCTAACCCTGATCCTCTTGTCCCACAGATATCCAGAACCCTCTAAGAAGTTCCTGCTTATTATTACTTCAGAGAATGTGGTTAGAGACATGACCCTGCCGTGTACCAGCTGAGAGACTCTAAATCCAGTGACAAGTCTG |
| D3 | TC15P2_pool1_116 | GAGATCATGTCCTAACCCTGATCCTCTTGTCCCACAGATATCCAGAACCCTCTAAGAAGTTCCTGCTATTAAATACTTCATTGAATGTGGTTAGAGACATGACCCTGCCGTGTACCAGCTGAGAGACTCTAAATCCAGTGACAAGTCTG |
| E3 | TC15P2_pool1_117 | GAGATCATGTCCTAACCCTGATCCTCTTGTCCCACAGATATCCAGAACCCTCTAAGAAGTTCCTGCTAAAAAATTCTTCCATGAATGTGGTTAGAGACATGACCCTGCCGTGTACCAGCTGAGAGACTCTAAATCCAGTGACAAGTCTG |
| F3 | TC15P2_pool1_118 | GAGATCATGTCCTAACCCTGATCCTCTTGTCCCACAGATATCCAGAACCCTCTAAGAAGTTCCTGCTTTTTAAAACTTCCCAGAATGTGGTTAGAGACATGACCCTGCCGTGTACCAGCTGAGAGACTCTAAATCCAGTGACAAGTCTG |
| G3 | TC15P2_pool1_119 | GAGATCATGTCCTAACCCTGATCCTCTTGTCCCACAGATATCCAGAACCCTCTAAGAAGTTCCTGCTTAAATTTTCTTCCGTGAATGTGGTTAGAGACATGACCCTGCCGTGTACCAGCTGAGAGACTCTAAATCCAGTGACAAGTCTG |
| H3 | TC15P2_pool1_120 | GAGATCATGTCCTAACCCTGATCCTCTTGTCCCACAGATATCCAGAACCCTCTAAGAAGTTCCTGCTTTATTTATCTTCCTAGAATGTGGTTAGAGACATGACCCTGCCGTGTACCAGCTGAGAGACTCTAAATCCAGTGACAAGTCTG |
| A4 | TC15P2_pool1_121 | GAGATCATGTCCTAACCCTGATCCTCTTGTCCCACAGATATCCAGAACCCTCTAAGAAGTTCCTGCTATAAAATACTTCGAAGAATGTGGTTAGAGACATGACCCTGCCGTGTACCAGCTGAGAGACTCTAAATCCAGTGACAAGTCTG |
| B4 | TC15P2_pool1_122 | GAGATCATGTCCTAACCCTGATCCTCTTGTCCCACAGATATCCAGAACCCTCTAAGAAGTTCCTGCTTTTTTTTACTTCGCAGAATGTGGTTAGAGACATGACCCTGCCGTGTACCAGCTGAGAGACTCTAAATCCAGTGACAAGTCTG |
| C4 | TC15P2_pool1_123 | GAGATCATGTCCTAACCCTGATCCTCTTGTCCCACAGATATCCAGAACCCTCTAAGAAGTTCCTGCTTATTAAATCTTCGGAGAATGTGGTTAGAGACATGACCCTGCCGTGTACCAGCTGAGAGACTCTAAATCCAGTGACAAGTCTG |
| D4 | TC15P2_pool1_124 | GAGATCATGTCCTAACCCTGATCCTCTTGTCCCACAGATATCCAGAACCCTCTAAGAAGTTCCTGCTATTAATTACTTCGTAGAATGTGGTTAGAGACATGACCCTGCCGTGTACCAGCTGAGAGACTCTAAATCCAGTGACAAGTCTG |
| E4 | TC15P2_pool1_125 | GAGATCATGTCCTAACCCTGATCCTCTTGTCCCACAGATATCCAGAACCCTCTAAGAAGTTCCTGCTAAATATTACTTCTAAGAATGTGGTTAGAGACATGACCCTGCCGTGTACCAGCTGAGAGACTCTAAATCCAGTGACAAGTCTG |
|  |  |  |
| F4 | TC15P2_pool2_1 | GAGATCATGTCCTAACCCTGATCCTCTTGTCCCACAGATATCCAGAACCCTCTAAGAAGTTCCTGCTTTTAAAATCTTCTCAGAATGTGGTTAGAGACATGACCCTGCCGTGTACCAGCTGAGAGACTCTAAATCCAGTGACAAGTCTG |
| G4 | TC15P2_pool2_2 | GAGATCATGTCCTAACCCTGATCCTCTTGTCCCACAGATATCCAGAACCCTCTAAGAAGTTCCTGCTATTTTAATCTTCTGTGAATGTGGTTAGAGACATGACCCTGCCGTGTACCAGCTGAGAGACTCTAAATCCAGTGACAAGTCTG |
| H4 | TC15P2_pool2_3 | GAGATCATGTCCTAACCCTGATCCTCTTGTCCCACAGATATCCAGAACCCTCTAAGAAGTTCCTGCTATAAATAACTTCTTAGAATGTGGTTAGAGACATGACCCTGCCGTGTACCAGCTGAGAGACTCTAAATCCAGTGACAAGTCTG |
| A5 | TC15P2_pool2_4 | GAGATCATGTCCTAACCCTGATCCTCTTGTCCCACAGATATCCAGAACCCTCTAAGAAGTTCCTGCTTTATATTTGATCAAAGAATGTGGTTAGAGACATGACCCTGCCGTGTACCAGCTGAGAGACTCTAAATCCAGTGACAAGTCTG |
| B5 | TC15P2_pool2_5 | GAGATCATGTCCTAACCCTGATCCTCTTGTCCCACAGATATCCAGAACCCTCTAAGAAGTTCCTGCTAATATATTGATCACAGAATGTGGTTAGAGACATGACCCTGCCGTGTACCAGCTGAGAGACTCTAAATCCAGTGACAAGTCTG |
| C5 | TC15P2_pool2_6 | GAGATCATGTCCTAACCCTGATCCTCTTGTCCCACAGATATCCAGAACCCTCTAAGAAGTTCCTGCTTTTAAATAGATCAGAGAATGTGGTTAGAGACATGACCCTGCCGTGTACCAGCTGAGAGACTCTAAATCCAGTGACAAGTCTG |
| D5 | TC15P2_pool2_7 | GAGATCATGTCCTAACCCTGATCCTCTTGTCCCACAGATATCCAGAACCCTCTAAGAAGTTCCTGCTTTAAAAAAGATCATAGAATGTGGTTAGAGACATGACCCTGCCGTGTACCAGCTGAGAGACTCTAAATCCAGTGACAAGTCTG |
| E5 | TC15P2_pool2_8 | GAGATCATGTCCTAACCCTGATCCTCTTGTCCCACAGATATCCAGAACCCTCTAAGAAGTTCCTGCTTATTATTTGATCCATGAATGTGGTTAGAGACATGACCCTGCCGTGTACCAGCTGAGAGACTCTAAATCCAGTGACAAGTCTG |
| F5 | TC15P2_pool2_9 | GAGATCATGTCCTAACCCTGATCCTCTTGTCCCACAGATATCCAGAACCCTCTAAGAAGTTCCTGCTTTATTTAAGATCCCTGAATGTGGTTAGAGACATGACCCTGCCGTGTACCAGCTGAGAGACTCTAAATCCAGTGACAAGTCTG |
| G5 | TC15P2_pool2_10 | GAGATCATGTCCTAACCCTGATCCTCTTGTCCCACAGATATCCAGAACCCTCTAAGAAGTTCCTGCTAAAATAATGATCCGAGAATGTGGTTAGAGACATGACCCTGCCGTGTACCAGCTGAGAGACTCTAAATCCAGTGACAAGTCTG |
| H5 | TC15P2_pool2_11 | GAGATCATGTCCTAACCCTGATCCTCTTGTCCCACAGATATCCAGAACCCTCTAAGAAGTTCCTGCTATATTAAAGATCCTTGAATGTGGTTAGAGACATGACCCTGCCGTGTACCAGCTGAGAGACTCTAAATCCAGTGACAAGTCTG |
| A6 | TC15P2_pool2_12 | GAGATCATGTCCTAACCCTGATCCTCTTGTCCCACAGATATCCAGAACCCTCTAAGAAGTTCCTGCTATTAATATGATCGAAGAATGTGGTTAGAGACATGACCCTGCCGTGTACCAGCTGAGAGACTCTAAATCCAGTGACAAGTCTG |
| B6 | TC15P2_pool2_13 | GAGATCATGTCCTAACCCTGATCCTCTTGTCCCACAGATATCCAGAACCCTCTAAGAAGTTCCTGCTATTATATTGATCGCTGAATGTGGTTAGAGACATGACCCTGCCGTGTACCAGCTGAGAGACTCTAAATCCAGTGACAAGTCTG |
| C6 | TC15P2_pool2_14 | GAGATCATGTCCTAACCCTGATCCTCTTGTCCCACAGATATCCAGAACCCTCTAAGAAGTTCCTGCTATATATTTGATCGGTGAATGTGGTTAGAGACATGACCCTGCCGTGTACCAGCTGAGAGACTCTAAATCCAGTGACAAGTCTG |
| D6 | TC15P2_pool2_15 | GAGATCATGTCCTAACCCTGATCCTCTTGTCCCACAGATATCCAGAACCCTCTAAGAAGTTCCTGCTAAAATATAGATCGTTGAATGTGGTTAGAGACATGACCCTGCCGTGTACCAGCTGAGAGACTCTAAATCCAGTGACAAGTCTG |
| E6 | TC15P2_pool2_16 | GAGATCATGTCCTAACCCTGATCCTCTTGTCCCACAGATATCCAGAACCCTCTAAGAAGTTCCTGCTTATAATAAGATCTATGAATGTGGTTAGAGACATGACCCTGCCGTGTACCAGCTGAGAGACTCTAAATCCAGTGACAAGTCTG |
| F6 | TC15P2_pool2_17 | GAGATCATGTCCTAACCCTGATCCTCTTGTCCCACAGATATCCAGAACCCTCTAAGAAGTTCCTGCTTAATAAATGATCTCTGAATGTGGTTAGAGACATGACCCTGCCGTGTACCAGCTGAGAGACTCTAAATCCAGTGACAAGTCTG |
| G6 | TC15P2_pool2_18 | GAGATCATGTCCTAACCCTGATCCTCTTGTCCCACAGATATCCAGAACCCTCTAAGAAGTTCCTGCTAATAATAAGATCTGAGAATGTGGTTAGAGACATGACCCTGCCGTGTACCAGCTGAGAGACTCTAAATCCAGTGACAAGTCTG |
| H6 | TC15P2_pool2_19 | GAGATCATGTCCTAACCCTGATCCTCTTGTCCCACAGATATCCAGAACCCTCTAAGAAGTTCCTGCTATAATAATGATCTTTGAATGTGGTTAGAGACATGACCCTGCCGTGTACCAGCTGAGAGACTCTAAATCCAGTGACAAGTCTG |
| A7 | TC15P2_pool2_20 | GAGATCATGTCCTAACCCTGATCCTCTTGTCCCACAGATATCCAGAACCCTCTAAGAAGTTCCTGCTTTATTAAAGCTCAAAGAATGTGGTTAGAGACATGACCCTGCCGTGTACCAGCTGAGAGACTCTAAATCCAGTGACAAGTCTG |
| B7 | TC15P2_pool2_21 | GAGATCATGTCCTAACCCTGATCCTCTTGTCCCACAGATATCCAGAACCCTCTAAGAAGTTCCTGCTAATTATTTGCTCACAGAATGTGGTTAGAGACATGACCCTGCCGTGTACCAGCTGAGAGACTCTAAATCCAGTGACAAGTCTG |
| C7 | TC15P2_pool2_22 | GAGATCATGTCCTAACCCTGATCCTCTTGTCCCACAGATATCCAGAACCCTCTAAGAAGTTCCTGCTAAATAATTGCTCAGAGAATGTGGTTAGAGACATGACCCTGCCGTGTACCAGCTGAGAGACTCTAAATCCAGTGACAAGTCTG |
| D7 | TC15P2_pool2_23 | GAGATCATGTCCTAACCCTGATCCTCTTGTCCCACAGATATCCAGAACCCTCTAAGAAGTTCCTGCTTATTTAATGCTCATTGAATGTGGTTAGAGACATGACCCTGCCGTGTACCAGCTGAGAGACTCTAAATCCAGTGACAAGTCTG |
| E7 | TC15P2_pool2_24 | GAGATCATGTCCTAACCCTGATCCTCTTGTCCCACAGATATCCAGAACCCTCTAAGAAGTTCCTGCTTTAATTTAGCTCCAAGAATGTGGTTAGAGACATGACCCTGCCGTGTACCAGCTGAGAGACTCTAAATCCAGTGACAAGTCTG |
| F7 | TC15P2_pool2_25 | GAGATCATGTCCTAACCCTGATCCTCTTGTCCCACAGATATCCAGAACCCTCTAAGAAGTTCCTGCTAATTTAAAGCTCCCTGAATGTGGTTAGAGACATGACCCTGCCGTGTACCAGCTGAGAGACTCTAAATCCAGTGACAAGTCTG |
| G7 | TC15P2_pool2_26 | GAGATCATGTCCTAACCCTGATCCTCTTGTCCCACAGATATCCAGAACCCTCTAAGAAGTTCCTGCTTATTTATAGCTCCGTGAATGTGGTTAGAGACATGACCCTGCCGTGTACCAGCTGAGAGACTCTAAATCCAGTGACAAGTCTG |
| H7 | TC15P2_pool2_27 | GAGATCATGTCCTAACCCTGATCCTCTTGTCCCACAGATATCCAGAACCCTCTAAGAAGTTCCTGCTTTTAATTAGCTCCTTGAATGTGGTTAGAGACATGACCCTGCCGTGTACCAGCTGAGAGACTCTAAATCCAGTGACAAGTCTG |
| A8 | TC15P2_pool2_28 | GAGATCATGTCCTAACCCTGATCCTCTTGTCCCACAGATATCCAGAACCCTCTAAGAAGTTCCTGCTTTAATATAGCTCGATGAATGTGGTTAGAGACATGACCCTGCCGTGTACCAGCTGAGAGACTCTAAATCCAGTGACAAGTCTG |
| B8 | TC15P2_pool2_29 | GAGATCATGTCCTAACCCTGATCCTCTTGTCCCACAGATATCCAGAACCCTCTAAGAAGTTCCTGCTAAATTTAAGCTCGCAGAATGTGGTTAGAGACATGACCCTGCCGTGTACCAGCTGAGAGACTCTAAATCCAGTGACAAGTCTG |
| C8 | TC15P2_pool2_30 | GAGATCATGTCCTAACCCTGATCCTCTTGTCCCACAGATATCCAGAACCCTCTAAGAAGTTCCTGCTATAAAAAAGCTCGGTGAATGTGGTTAGAGACATGACCCTGCCGTGTACCAGCTGAGAGACTCTAAATCCAGTGACAAGTCTG |
| D8 | TC15P2_pool2_31 | GAGATCATGTCCTAACCCTGATCCTCTTGTCCCACAGATATCCAGAACCCTCTAAGAAGTTCCTGCTTTAATAATGCTCGTAGAATGTGGTTAGAGACATGACCCTGCCGTGTACCAGCTGAGAGACTCTAAATCCAGTGACAAGTCTG |
| E8 | TC15P2_pool2_32 | GAGATCATGTCCTAACCCTGATCCTCTTGTCCCACAGATATCCAGAACCCTCTAAGAAGTTCCTGCTATTAATAAGCTCTATGAATGTGGTTAGAGACATGACCCTGCCGTGTACCAGCTGAGAGACTCTAAATCCAGTGACAAGTCTG |
| F8 | TC15P2_pool2_33 | GAGATCATGTCCTAACCCTGATCCTCTTGTCCCACAGATATCCAGAACCCTCTAAGAAGTTCCTGCTATATTAATGCTCTCAGAATGTGGTTAGAGACATGACCCTGCCGTGTACCAGCTGAGAGACTCTAAATCCAGTGACAAGTCTG |
| G8 | TC15P2_pool2_34 | GAGATCATGTCCTAACCCTGATCCTCTTGTCCCACAGATATCCAGAACCCTCTAAGAAGTTCCTGCTTTATAATTGCTCTGTGAATGTGGTTAGAGACATGACCCTGCCGTGTACCAGCTGAGAGACTCTAAATCCAGTGACAAGTCTG |
| H8 | TC15P2_pool2_35 | GAGATCATGTCCTAACCCTGATCCTCTTGTCCCACAGATATCCAGAACCCTCTAAGAAGTTCCTGCTTATATATTGCTCTTTGAATGTGGTTAGAGACATGACCCTGCCGTGTACCAGCTGAGAGACTCTAAATCCAGTGACAAGTCTG |
| A9 | TC15P2_pool2_36 | GAGATCATGTCCTAACCCTGATCCTCTTGTCCCACAGATATCCAGAACCCTCTAAGAAGTTCCTGCTTATATTTTGGTCAAAGAATGTGGTTAGAGACATGACCCTGCCGTGTACCAGCTGAGAGACTCTAAATCCAGTGACAAGTCTG |
| B9 | TC15P2_pool2_37 | GAGATCATGTCCTAACCCTGATCCTCTTGTCCCACAGATATCCAGAACCCTCTAAGAAGTTCCTGCTTAATATTAGGTCACTGAATGTGGTTAGAGACATGACCCTGCCGTGTACCAGCTGAGAGACTCTAAATCCAGTGACAAGTCTG |
| C9 | TC15P2_pool2_38 | GAGATCATGTCCTAACCCTGATCCTCTTGTCCCACAGATATCCAGAACCCTCTAAGAAGTTCCTGCTTAAAATTTGGTCAGAGAATGTGGTTAGAGACATGACCCTGCCGTGTACCAGCTGAGAGACTCTAAATCCAGTGACAAGTCTG |
| D9 | TC15P2_pool2_39 | GAGATCATGTCCTAACCCTGATCCTCTTGTCCCACAGATATCCAGAACCCTCTAAGAAGTTCCTGCTATATAATAGGTCATTGAATGTGGTTAGAGACATGACCCTGCCGTGTACCAGCTGAGAGACTCTAAATCCAGTGACAAGTCTG |
| E9 | TC15P2_pool2_40 | GAGATCATGTCCTAACCCTGATCCTCTTGTCCCACAGATATCCAGAACCCTCTAAGAAGTTCCTGCTAATTAAAAGGTCCAAGAATGTGGTTAGAGACATGACCCTGCCGTGTACCAGCTGAGAGACTCTAAATCCAGTGACAAGTCTG |
| F9 | TC15P2_pool2_41 | GAGATCATGTCCTAACCCTGATCCTCTTGTCCCACAGATATCCAGAACCCTCTAAGAAGTTCCTGCTAATAAAATGGTCCCTGAATGTGGTTAGAGACATGACCCTGCCGTGTACCAGCTGAGAGACTCTAAATCCAGTGACAAGTCTG |
| G9 | TC15P2_pool2_42 | GAGATCATGTCCTAACCCTGATCCTCTTGTCCCACAGATATCCAGAACCCTCTAAGAAGTTCCTGCTAAAAATAAGGTCCGTGAATGTGGTTAGAGACATGACCCTGCCGTGTACCAGCTGAGAGACTCTAAATCCAGTGACAAGTCTG |
| H9 | TC15P2_pool2_43 | GAGATCATGTCCTAACCCTGATCCTCTTGTCCCACAGATATCCAGAACCCTCTAAGAAGTTCCTGCTATTTTTAAGGTCCTTGAATGTGGTTAGAGACATGACCCTGCCGTGTACCAGCTGAGAGACTCTAAATCCAGTGACAAGTCTG |
| A10 | TC15P2_pool2_44 | GAGATCATGTCCTAACCCTGATCCTCTTGTCCCACAGATATCCAGAACCCTCTAAGAAGTTCCTGCTTTTTTAAAGGTCGATGAATGTGGTTAGAGACATGACCCTGCCGTGTACCAGCTGAGAGACTCTAAATCCAGTGACAAGTCTG |
| B10 | TC15P2_pool2_45 | GAGATCATGTCCTAACCCTGATCCTCTTGTCCCACAGATATCCAGAACCCTCTAAGAAGTTCCTGCTATAATTATGGTCGCAGAATGTGGTTAGAGACATGACCCTGCCGTGTACCAGCTGAGAGACTCTAAATCCAGTGACAAGTCTG |
| C10 | TC15P2_pool2_46 | GAGATCATGTCCTAACCCTGATCCTCTTGTCCCACAGATATCCAGAACCCTCTAAGAAGTTCCTGCTAATAAATAGGTCGGAGAATGTGGTTAGAGACATGACCCTGCCGTGTACCAGCTGAGAGACTCTAAATCCAGTGACAAGTCTG |
| D10 | TC15P2_pool2_47 | GAGATCATGTCCTAACCCTGATCCTCTTGTCCCACAGATATCCAGAACCCTCTAAGAAGTTCCTGCTTATATAATGGTCGTAGAATGTGGTTAGAGACATGACCCTGCCGTGTACCAGCTGAGAGACTCTAAATCCAGTGACAAGTCTG |
| E10 | TC15P2_pool2_48 | GAGATCATGTCCTAACCCTGATCCTCTTGTCCCACAGATATCCAGAACCCTCTAAGAAGTTCCTGCTTAAAAAAAGGTCTATGAATGTGGTTAGAGACATGACCCTGCCGTGTACCAGCTGAGAGACTCTAAATCCAGTGACAAGTCTG |
| F10 | TC15P2_pool2_49 | GAGATCATGTCCTAACCCTGATCCTCTTGTCCCACAGATATCCAGAACCCTCTAAGAAGTTCCTGCTATTTAATTGGTCTCAGAATGTGGTTAGAGACATGACCCTGCCGTGTACCAGCTGAGAGACTCTAAATCCAGTGACAAGTCTG |
| G10 | TC15P2_pool2_50 | GAGATCATGTCCTAACCCTGATCCTCTTGTCCCACAGATATCCAGAACCCTCTAAGAAGTTCCTGCTTTATAATAGGTCTGAGAATGTGGTTAGAGACATGACCCTGCCGTGTACCAGCTGAGAGACTCTAAATCCAGTGACAAGTCTG |
| H10 | TC15P2_pool2_51 | GAGATCATGTCCTAACCCTGATCCTCTTGTCCCACAGATATCCAGAACCCTCTAAGAAGTTCCTGCTTATATATAGGTCTTAGAATGTGGTTAGAGACATGACCCTGCCGTGTACCAGCTGAGAGACTCTAAATCCAGTGACAAGTCTG |
| A11 | TC15P2_pool2_52 | GAGATCATGTCCTAACCCTGATCCTCTTGTCCCACAGATATCCAGAACCCTCTAAGAAGTTCCTGCTAAAAAAAAGTTCAAAGAATGTGGTTAGAGACATGACCCTGCCGTGTACCAGCTGAGAGACTCTAAATCCAGTGACAAGTCTG |
| B11 | TC15P2_pool2_53 | GAGATCATGTCCTAACCCTGATCCTCTTGTCCCACAGATATCCAGAACCCTCTAAGAAGTTCCTGCTTATTTTAAGTTCACAGAATGTGGTTAGAGACATGACCCTGCCGTGTACCAGCTGAGAGACTCTAAATCCAGTGACAAGTCTG |
| C11 | TC15P2_pool2_54 | GAGATCATGTCCTAACCCTGATCCTCTTGTCCCACAGATATCCAGAACCCTCTAAGAAGTTCCTGCTAATTTTTAGTTCAGAGAATGTGGTTAGAGACATGACCCTGCCGTGTACCAGCTGAGAGACTCTAAATCCAGTGACAAGTCTG |
| D11 | TC15P2_pool2_55 | GAGATCATGTCCTAACCCTGATCCTCTTGTCCCACAGATATCCAGAACCCTCTAAGAAGTTCCTGCTAAATTATAGTTCATAGAATGTGGTTAGAGACATGACCCTGCCGTGTACCAGCTGAGAGACTCTAAATCCAGTGACAAGTCTG |
| E11 | TC15P2_pool2_56 | GAGATCATGTCCTAACCCTGATCCTCTTGTCCCACAGATATCCAGAACCCTCTAAGAAGTTCCTGCTTTTAAATTGTTCCATGAATGTGGTTAGAGACATGACCCTGCCGTGTACCAGCTGAGAGACTCTAAATCCAGTGACAAGTCTG |
| F11 | TC15P2_pool2_57 | GAGATCATGTCCTAACCCTGATCCTCTTGTCCCACAGATATCCAGAACCCTCTAAGAAGTTCCTGCTTTTAATTTGTTCCCAGAATGTGGTTAGAGACATGACCCTGCCGTGTACCAGCTGAGAGACTCTAAATCCAGTGACAAGTCTG |
| G11 | TC15P2_pool2_58 | GAGATCATGTCCTAACCCTGATCCTCTTGTCCCACAGATATCCAGAACCCTCTAAGAAGTTCCTGCTATAATTAAGTTCCGTGAATGTGGTTAGAGACATGACCCTGCCGTGTACCAGCTGAGAGACTCTAAATCCAGTGACAAGTCTG |
| H11 | TC15P2_pool2_59 | GAGATCATGTCCTAACCCTGATCCTCTTGTCCCACAGATATCCAGAACCCTCTAAGAAGTTCCTGCTTTAAAATTGTTCCTAGAATGTGGTTAGAGACATGACCCTGCCGTGTACCAGCTGAGAGACTCTAAATCCAGTGACAAGTCTG |
| A12 | TC15P2_pool2_60 | GAGATCATGTCCTAACCCTGATCCTCTTGTCCCACAGATATCCAGAACCCTCTAAGAAGTTCCTGCTTTATTTTTGTTCGATGAATGTGGTTAGAGACATGACCCTGCCGTGTACCAGCTGAGAGACTCTAAATCCAGTGACAAGTCTG |
| B12 | TC15P2_pool2_61 | GAGATCATGTCCTAACCCTGATCCTCTTGTCCCACAGATATCCAGAACCCTCTAAGAAGTTCCTGCTTAATTAAAGTTCGCTGAATGTGGTTAGAGACATGACCCTGCCGTGTACCAGCTGAGAGACTCTAAATCCAGTGACAAGTCTG |
| C12 | TC15P2_pool2_62 | GAGATCATGTCCTAACCCTGATCCTCTTGTCCCACAGATATCCAGAACCCTCTAAGAAGTTCCTGCTTATAAAAAGTTCGGAGAATGTGGTTAGAGACATGACCCTGCCGTGTACCAGCTGAGAGACTCTAAATCCAGTGACAAGTCTG |
| D12 | TC15P2_pool2_63 | GAGATCATGTCCTAACCCTGATCCTCTTGTCCCACAGATATCCAGAACCCTCTAAGAAGTTCCTGCTTATATTTAGTTCGTTGAATGTGGTTAGAGACATGACCCTGCCGTGTACCAGCTGAGAGACTCTAAATCCAGTGACAAGTCTG |
| E12 | TC15P2_pool2_64 | GAGATCATGTCCTAACCCTGATCCTCTTGTCCCACAGATATCCAGAACCCTCTAAGAAGTTCCTGCTTAAATAATGTTCTATGAATGTGGTTAGAGACATGACCCTGCCGTGTACCAGCTGAGAGACTCTAAATCCAGTGACAAGTCTG |
| F12 | TC15P2_pool2_65 | GAGATCATGTCCTAACCCTGATCCTCTTGTCCCACAGATATCCAGAACCCTCTAAGAAGTTCCTGCTAATTTTATGTTCTCAGAATGTGGTTAGAGACATGACCCTGCCGTGTACCAGCTGAGAGACTCTAAATCCAGTGACAAGTCTG |
| G12 | TC15P2_pool2_66 | GAGATCATGTCCTAACCCTGATCCTCTTGTCCCACAGATATCCAGAACCCTCTAAGAAGTTCCTGCTATTTATTAGTTCTGTGAATGTGGTTAGAGACATGACCCTGCCGTGTACCAGCTGAGAGACTCTAAATCCAGTGACAAGTCTG |
| H12 | TC15P2_pool2_67 | GAGATCATGTCCTAACCCTGATCCTCTTGTCCCACAGATATCCAGAACCCTCTAAGAAGTTCCTGCTAATTTTTTGTTCTTTGAATGTGGTTAGAGACATGACCCTGCCGTGTACCAGCTGAGAGACTCTAAATCCAGTGACAAGTCTG |
| A1 | TC15P3_pool2_68 | GAGATCATGTCCTAACCCTGATCCTCTTGTCCCACAGATATCCAGAACCCTCTAAGAAGTTCCTGCTTTTTAATATATCAATGAATGTGGTTAGAGACATGACCCTGCCGTGTACCAGCTGAGAGACTCTAAATCCAGTGACAAGTCTG |
| B1 | TC15P3_pool2_69 | GAGATCATGTCCTAACCCTGATCCTCTTGTCCCACAGATATCCAGAACCCTCTAAGAAGTTCCTGCTAAATTTTATATCACTGAATGTGGTTAGAGACATGACCCTGCCGTGTACCAGCTGAGAGACTCTAAATCCAGTGACAAGTCTG |
| C1 | TC15P3_pool2_70 | GAGATCATGTCCTAACCCTGATCCTCTTGTCCCACAGATATCCAGAACCCTCTAAGAAGTTCCTGCTTTTTAAATTATCAGTGAATGTGGTTAGAGACATGACCCTGCCGTGTACCAGCTGAGAGACTCTAAATCCAGTGACAAGTCTG |
| D1 | TC15P3_pool2_71 | GAGATCATGTCCTAACCCTGATCCTCTTGTCCCACAGATATCCAGAACCCTCTAAGAAGTTCCTGCTATATTTTTTATCATAGAATGTGGTTAGAGACATGACCCTGCCGTGTACCAGCTGAGAGACTCTAAATCCAGTGACAAGTCTG |
| E1 | TC15P3_pool2_72 | GAGATCATGTCCTAACCCTGATCCTCTTGTCCCACAGATATCCAGAACCCTCTAAGAAGTTCCTGCTTATAAATTTATCCAAGAATGTGGTTAGAGACATGACCCTGCCGTGTACCAGCTGAGAGACTCTAAATCCAGTGACAAGTCTG |
| F1 | TC15P3_pool2_73 | GAGATCATGTCCTAACCCTGATCCTCTTGTCCCACAGATATCCAGAACCCTCTAAGAAGTTCCTGCTAATATTAATATCCCTGAATGTGGTTAGAGACATGACCCTGCCGTGTACCAGCTGAGAGACTCTAAATCCAGTGACAAGTCTG |
| G1 | TC15P3_pool2_74 | GAGATCATGTCCTAACCCTGATCCTCTTGTCCCACAGATATCCAGAACCCTCTAAGAAGTTCCTGCTAAATTTATTATCCGTGAATGTGGTTAGAGACATGACCCTGCCGTGTACCAGCTGAGAGACTCTAAATCCAGTGACAAGTCTG |
| H1 | TC15P3_pool2_75 | GAGATCATGTCCTAACCCTGATCCTCTTGTCCCACAGATATCCAGAACCCTCTAAGAAGTTCCTGCTTTATAAAATATCCTTGAATGTGGTTAGAGACATGACCCTGCCGTGTACCAGCTGAGAGACTCTAAATCCAGTGACAAGTCTG |
| A2 | TC15P3_pool2_76 | GAGATCATGTCCTAACCCTGATCCTCTTGTCCCACAGATATCCAGAACCCTCTAAGAAGTTCCTGCTTAAAAATATATCGAAGAATGTGGTTAGAGACATGACCCTGCCGTGTACCAGCTGAGAGACTCTAAATCCAGTGACAAGTCTG |
| B2 | TC15P3_pool2_77 | GAGATCATGTCCTAACCCTGATCCTCTTGTCCCACAGATATCCAGAACCCTCTAAGAAGTTCCTGCTATTAATTTTATCGCTGAATGTGGTTAGAGACATGACCCTGCCGTGTACCAGCTGAGAGACTCTAAATCCAGTGACAAGTCTG |
| C2 | TC15P3_pool2_78 | GAGATCATGTCCTAACCCTGATCCTCTTGTCCCACAGATATCCAGAACCCTCTAAGAAGTTCCTGCTTATATAAATATCGGTGAATGTGGTTAGAGACATGACCCTGCCGTGTACCAGCTGAGAGACTCTAAATCCAGTGACAAGTCTG |
| D2 | TC15P3_pool2_79 | GAGATCATGTCCTAACCCTGATCCTCTTGTCCCACAGATATCCAGAACCCTCTAAGAAGTTCCTGCTATAAATTATATCGTTGAATGTGGTTAGAGACATGACCCTGCCGTGTACCAGCTGAGAGACTCTAAATCCAGTGACAAGTCTG |
| E2 | TC15P3_pool2_80 | GAGATCATGTCCTAACCCTGATCCTCTTGTCCCACAGATATCCAGAACCCTCTAAGAAGTTCCTGCTTTTATTATTATCTATGAATGTGGTTAGAGACATGACCCTGCCGTGTACCAGCTGAGAGACTCTAAATCCAGTGACAAGTCTG |
| F2 | TC15P3_pool2_81 | GAGATCATGTCCTAACCCTGATCCTCTTGTCCCACAGATATCCAGAACCCTCTAAGAAGTTCCTGCTATAAATTTTATCTCAGAATGTGGTTAGAGACATGACCCTGCCGTGTACCAGCTGAGAGACTCTAAATCCAGTGACAAGTCTG |
| G2 | TC15P3_pool2_82 | GAGATCATGTCCTAACCCTGATCCTCTTGTCCCACAGATATCCAGAACCCTCTAAGAAGTTCCTGCTATTATTTATATCTGTGAATGTGGTTAGAGACATGACCCTGCCGTGTACCAGCTGAGAGACTCTAAATCCAGTGACAAGTCTG |
| H2 | TC15P3_pool2_83 | GAGATCATGTCCTAACCCTGATCCTCTTGTCCCACAGATATCCAGAACCCTCTAAGAAGTTCCTGCTAAATAAAATATCTTTGAATGTGGTTAGAGACATGACCCTGCCGTGTACCAGCTGAGAGACTCTAAATCCAGTGACAAGTCTG |
| A3 | TC15P3_pool2_84 | GAGATCATGTCCTAACCCTGATCCTCTTGTCCCACAGATATCCAGAACCCTCTAAGAAGTTCCTGCTTAATTAATTCTCAAAGAATGTGGTTAGAGACATGACCCTGCCGTGTACCAGCTGAGAGACTCTAAATCCAGTGACAAGTCTG |
| B3 | TC15P3_pool2_85 | GAGATCATGTCCTAACCCTGATCCTCTTGTCCCACAGATATCCAGAACCCTCTAAGAAGTTCCTGCTAATTATAATCTCACTGAATGTGGTTAGAGACATGACCCTGCCGTGTACCAGCTGAGAGACTCTAAATCCAGTGACAAGTCTG |
| C3 | TC15P3_pool2_86 | GAGATCATGTCCTAACCCTGATCCTCTTGTCCCACAGATATCCAGAACCCTCTAAGAAGTTCCTGCTATATTATTTCTCAGTGAATGTGGTTAGAGACATGACCCTGCCGTGTACCAGCTGAGAGACTCTAAATCCAGTGACAAGTCTG |
| D3 | TC15P3_pool2_87 | GAGATCATGTCCTAACCCTGATCCTCTTGTCCCACAGATATCCAGAACCCTCTAAGAAGTTCCTGCTTTATAAATTCTCATAGAATGTGGTTAGAGACATGACCCTGCCGTGTACCAGCTGAGAGACTCTAAATCCAGTGACAAGTCTG |
| E3 | TC15P3_pool2_88 | GAGATCATGTCCTAACCCTGATCCTCTTGTCCCACAGATATCCAGAACCCTCTAAGAAGTTCCTGCTTTTTATATTCTCCAAGAATGTGGTTAGAGACATGACCCTGCCGTGTACCAGCTGAGAGACTCTAAATCCAGTGACAAGTCTG |
| F3 | TC15P3_pool2_89 | GAGATCATGTCCTAACCCTGATCCTCTTGTCCCACAGATATCCAGAACCCTCTAAGAAGTTCCTGCTAAAATTTTTCTCCCAGAATGTGGTTAGAGACATGACCCTGCCGTGTACCAGCTGAGAGACTCTAAATCCAGTGACAAGTCTG |
| G3 | TC15P3_pool2_90 | GAGATCATGTCCTAACCCTGATCCTCTTGTCCCACAGATATCCAGAACCCTCTAAGAAGTTCCTGCTTATTAAAATCTCCGTGAATGTGGTTAGAGACATGACCCTGCCGTGTACCAGCTGAGAGACTCTAAATCCAGTGACAAGTCTG |
| H3 | TC15P3_pool2_91 | GAGATCATGTCCTAACCCTGATCCTCTTGTCCCACAGATATCCAGAACCCTCTAAGAAGTTCCTGCTTAAATTAATCTCCTAGAATGTGGTTAGAGACATGACCCTGCCGTGTACCAGCTGAGAGACTCTAAATCCAGTGACAAGTCTG |
| A4 | TC15P3_pool2_92 | GAGATCATGTCCTAACCCTGATCCTCTTGTCCCACAGATATCCAGAACCCTCTAAGAAGTTCCTGCTTAAATATTTCTCGAAGAATGTGGTTAGAGACATGACCCTGCCGTGTACCAGCTGAGAGACTCTAAATCCAGTGACAAGTCTG |
| B4 | TC15P3_pool2_93 | GAGATCATGTCCTAACCCTGATCCTCTTGTCCCACAGATATCCAGAACCCTCTAAGAAGTTCCTGCTAATAATTATCTCGCTGAATGTGGTTAGAGACATGACCCTGCCGTGTACCAGCTGAGAGACTCTAAATCCAGTGACAAGTCTG |
| C4 | TC15P3_pool2_94 | GAGATCATGTCCTAACCCTGATCCTCTTGTCCCACAGATATCCAGAACCCTCTAAGAAGTTCCTGCTATATAAATTCTCGGTGAATGTGGTTAGAGACATGACCCTGCCGTGTACCAGCTGAGAGACTCTAAATCCAGTGACAAGTCTG |
| D4 | TC15P3_pool2_95 | GAGATCATGTCCTAACCCTGATCCTCTTGTCCCACAGATATCCAGAACCCTCTAAGAAGTTCCTGCTTTAAATATTCTCGTTGAATGTGGTTAGAGACATGACCCTGCCGTGTACCAGCTGAGAGACTCTAAATCCAGTGACAAGTCTG |
| E4 | TC15P3_pool2_96 | GAGATCATGTCCTAACCCTGATCCTCTTGTCCCACAGATATCCAGAACCCTCTAAGAAGTTCCTGCTAAAAATATTCTCTAAGAATGTGGTTAGAGACATGACCCTGCCGTGTACCAGCTGAGAGACTCTAAATCCAGTGACAAGTCTG |
| F4 | TC15P3_pool2_97 | GAGATCATGTCCTAACCCTGATCCTCTTGTCCCACAGATATCCAGAACCCTCTAAGAAGTTCCTGCTATATATATTCTCTCAGAATGTGGTTAGAGACATGACCCTGCCGTGTACCAGCTGAGAGACTCTAAATCCAGTGACAAGTCTG |
| G4 | TC15P3_pool2_98 | GAGATCATGTCCTAACCCTGATCCTCTTGTCCCACAGATATCCAGAACCCTCTAAGAAGTTCCTGCTTTATTAATTCTCTGTGAATGTGGTTAGAGACATGACCCTGCCGTGTACCAGCTGAGAGACTCTAAATCCAGTGACAAGTCTG |
| H4 | TC15P3_pool2_99 | GAGATCATGTCCTAACCCTGATCCTCTTGTCCCACAGATATCCAGAACCCTCTAAGAAGTTCCTGCTATTATAAATCTCTTAGAATGTGGTTAGAGACATGACCCTGCCGTGTACCAGCTGAGAGACTCTAAATCCAGTGACAAGTCTG |
| A5 | TC15P3_pool2_100 | GAGATCATGTCCTAACCCTGATCCTCTTGTCCCACAGATATCCAGAACCCTCTAAGAAGTTCCTGCTTTTAAAAATGTCAATGAATGTGGTTAGAGACATGACCCTGCCGTGTACCAGCTGAGAGACTCTAAATCCAGTGACAAGTCTG |
| B5 | TC15P3_pool2_101 | GAGATCATGTCCTAACCCTGATCCTCTTGTCCCACAGATATCCAGAACCCTCTAAGAAGTTCCTGCTTATAAATATGTCACTGAATGTGGTTAGAGACATGACCCTGCCGTGTACCAGCTGAGAGACTCTAAATCCAGTGACAAGTCTG |
| C5 | TC15P3_pool2_102 | GAGATCATGTCCTAACCCTGATCCTCTTGTCCCACAGATATCCAGAACCCTCTAAGAAGTTCCTGCTTTTAATAATGTCAGAGAATGTGGTTAGAGACATGACCCTGCCGTGTACCAGCTGAGAGACTCTAAATCCAGTGACAAGTCTG |
| D5 | TC15P3_pool2_103 | GAGATCATGTCCTAACCCTGATCCTCTTGTCCCACAGATATCCAGAACCCTCTAAGAAGTTCCTGCTATAATTTTTGTCATTGAATGTGGTTAGAGACATGACCCTGCCGTGTACCAGCTGAGAGACTCTAAATCCAGTGACAAGTCTG |
| E5 | TC15P3_pool2_104 | GAGATCATGTCCTAACCCTGATCCTCTTGTCCCACAGATATCCAGAACCCTCTAAGAAGTTCCTGCTTTTTTATTTGTCCAAGAATGTGGTTAGAGACATGACCCTGCCGTGTACCAGCTGAGAGACTCTAAATCCAGTGACAAGTCTG |
| F5 | TC15P3_pool2_105 | GAGATCATGTCCTAACCCTGATCCTCTTGTCCCACAGATATCCAGAACCCTCTAAGAAGTTCCTGCTTAATTATTTGTCCCTGAATGTGGTTAGAGACATGACCCTGCCGTGTACCAGCTGAGAGACTCTAAATCCAGTGACAAGTCTG |
| G5 | TC15P3_pool2_106 | GAGATCATGTCCTAACCCTGATCCTCTTGTCCCACAGATATCCAGAACCCTCTAAGAAGTTCCTGCTATAAAAATTGTCCGAGAATGTGGTTAGAGACATGACCCTGCCGTGTACCAGCTGAGAGACTCTAAATCCAGTGACAAGTCTG |
| H5 | TC15P3_pool2_107 | GAGATCATGTCCTAACCCTGATCCTCTTGTCCCACAGATATCCAGAACCCTCTAAGAAGTTCCTGCTTATAATATTGTCCTAGAATGTGGTTAGAGACATGACCCTGCCGTGTACCAGCTGAGAGACTCTAAATCCAGTGACAAGTCTG |
| A6 | TC15P3_pool2_108 | GAGATCATGTCCTAACCCTGATCCTCTTGTCCCACAGATATCCAGAACCCTCTAAGAAGTTCCTGCTTAATATATTGTCGAAGAATGTGGTTAGAGACATGACCCTGCCGTGTACCAGCTGAGAGACTCTAAATCCAGTGACAAGTCTG |
| B6 | TC15P3_pool2_109 | GAGATCATGTCCTAACCCTGATCCTCTTGTCCCACAGATATCCAGAACCCTCTAAGAAGTTCCTGCTTAATTTTTTGTCGCAGAATGTGGTTAGAGACATGACCCTGCCGTGTACCAGCTGAGAGACTCTAAATCCAGTGACAAGTCTG |
| C6 | TC15P3_pool2_110 | GAGATCATGTCCTAACCCTGATCCTCTTGTCCCACAGATATCCAGAACCCTCTAAGAAGTTCCTGCTATTTAAAATGTCGGTGAATGTGGTTAGAGACATGACCCTGCCGTGTACCAGCTGAGAGACTCTAAATCCAGTGACAAGTCTG |
| D6 | TC15P3_pool2_111 | GAGATCATGTCCTAACCCTGATCCTCTTGTCCCACAGATATCCAGAACCCTCTAAGAAGTTCCTGCTATATAAAATGTCGTAGAATGTGGTTAGAGACATGACCCTGCCGTGTACCAGCTGAGAGACTCTAAATCCAGTGACAAGTCTG |
| E6 | TC15P3_pool2_112 | GAGATCATGTCCTAACCCTGATCCTCTTGTCCCACAGATATCCAGAACCCTCTAAGAAGTTCCTGCTATAATATTTGTCTAAGAATGTGGTTAGAGACATGACCCTGCCGTGTACCAGCTGAGAGACTCTAAATCCAGTGACAAGTCTG |
| F6 | TC15P3_pool2_113 | GAGATCATGTCCTAACCCTGATCCTCTTGTCCCACAGATATCCAGAACCCTCTAAGAAGTTCCTGCTATTATTTTTGTCTCAGAATGTGGTTAGAGACATGACCCTGCCGTGTACCAGCTGAGAGACTCTAAATCCAGTGACAAGTCTG |
| G6 | TC15P3_pool2_114 | GAGATCATGTCCTAACCCTGATCCTCTTGTCCCACAGATATCCAGAACCCTCTAAGAAGTTCCTGCTAATTAATATGTCTGTGAATGTGGTTAGAGACATGACCCTGCCGTGTACCAGCTGAGAGACTCTAAATCCAGTGACAAGTCTG |
| H6 | TC15P3_pool2_115 | GAGATCATGTCCTAACCCTGATCCTCTTGTCCCACAGATATCCAGAACCCTCTAAGAAGTTCCTGCTTATTATTATGTCTTAGAATGTGGTTAGAGACATGACCCTGCCGTGTACCAGCTGAGAGACTCTAAATCCAGTGACAAGTCTG |
| A7 | TC15P3_pool2_116 | GAGATCATGTCCTAACCCTGATCCTCTTGTCCCACAGATATCCAGAACCCTCTAAGAAGTTCCTGCTATTAAATATTTCAATGAATGTGGTTAGAGACATGACCCTGCCGTGTACCAGCTGAGAGACTCTAAATCCAGTGACAAGTCTG |
| B7 | TC15P3_pool2_117 | GAGATCATGTCCTAACCCTGATCCTCTTGTCCCACAGATATCCAGAACCCTCTAAGAAGTTCCTGCTAAAAAATTTTTCACTGAATGTGGTTAGAGACATGACCCTGCCGTGTACCAGCTGAGAGACTCTAAATCCAGTGACAAGTCTG |
| C7 | TC15P3_pool2_118 | GAGATCATGTCCTAACCCTGATCCTCTTGTCCCACAGATATCCAGAACCCTCTAAGAAGTTCCTGCTTTTTAAAATTTCAGAGAATGTGGTTAGAGACATGACCCTGCCGTGTACCAGCTGAGAGACTCTAAATCCAGTGACAAGTCTG |
| D7 | TC15P3_pool2_119 | GAGATCATGTCCTAACCCTGATCCTCTTGTCCCACAGATATCCAGAACCCTCTAAGAAGTTCCTGCTTAAATTTTTTTCATTGAATGTGGTTAGAGACATGACCCTGCCGTGTACCAGCTGAGAGACTCTAAATCCAGTGACAAGTCTG |
| E7 | TC15P3_pool2_120 | GAGATCATGTCCTAACCCTGATCCTCTTGTCCCACAGATATCCAGAACCCTCTAAGAAGTTCCTGCTTTATTTATTTTCCAAGAATGTGGTTAGAGACATGACCCTGCCGTGTACCAGCTGAGAGACTCTAAATCCAGTGACAAGTCTG |
| F7 | TC15P3_pool2_121 | GAGATCATGTCCTAACCCTGATCCTCTTGTCCCACAGATATCCAGAACCCTCTAAGAAGTTCCTGCTATAAAATATTTCCCAGAATGTGGTTAGAGACATGACCCTGCCGTGTACCAGCTGAGAGACTCTAAATCCAGTGACAAGTCTG |
| G7 | TC15P3_pool2_122 | GAGATCATGTCCTAACCCTGATCCTCTTGTCCCACAGATATCCAGAACCCTCTAAGAAGTTCCTGCTTTTTTTTATTTCCGAGAATGTGGTTAGAGACATGACCCTGCCGTGTACCAGCTGAGAGACTCTAAATCCAGTGACAAGTCTG |
| H7 | TC15P3_pool2_123 | GAGATCATGTCCTAACCCTGATCCTCTTGTCCCACAGATATCCAGAACCCTCTAAGAAGTTCCTGCTTATTAAATTTTCCTAGAATGTGGTTAGAGACATGACCCTGCCGTGTACCAGCTGAGAGACTCTAAATCCAGTGACAAGTCTG |
| A8 | TC15P3_pool2_124 | GAGATCATGTCCTAACCCTGATCCTCTTGTCCCACAGATATCCAGAACCCTCTAAGAAGTTCCTGCTATTAATTATTTCGAAGAATGTGGTTAGAGACATGACCCTGCCGTGTACCAGCTGAGAGACTCTAAATCCAGTGACAAGTCTG |
| B8 | TC15P3_pool2_125 | GAGATCATGTCCTAACCCTGATCCTCTTGTCCCACAGATATCCAGAACCCTCTAAGAAGTTCCTGCTAAATATTATTTCGCAGAATGTGGTTAGAGACATGACCCTGCCGTGTACCAGCTGAGAGACTCTAAATCCAGTGACAAGTCTG |
|  |  |  |
| A1 | TC15P3_pool3_1 | GAGATCATGTCCTAACCCTGATCCTCTTGTCCCACAGATATCCAGAACCCTCTAAGAAGTTCCTGCTTTTAAAATTTTCGGAGAATGTGGTTAGAGACATGACCCTGCCGTGTACCAGCTGAGAGACTCTAAATCCAGTGACAAGTCTG |
| B1 | TC15P3_pool3_2 | GAGATCATGTCCTAACCCTGATCCTCTTGTCCCACAGATATCCAGAACCCTCTAAGAAGTTCCTGCTATTTTAATTTTCGTTGAATGTGGTTAGAGACATGACCCTGCCGTGTACCAGCTGAGAGACTCTAAATCCAGTGACAAGTCTG |
| C1 | TC15P3_pool3_3 | GAGATCATGTCCTAACCCTGATCCTCTTGTCCCACAGATATCCAGAACCCTCTAAGAAGTTCCTGCTATAAATAATTTCTAAGAATGTGGTTAGAGACATGACCCTGCCGTGTACCAGCTGAGAGACTCTAAATCCAGTGACAAGTCTG |
| D1 | TC15P3_pool3_4 | GAGATCATGTCCTAACCCTGATCCTCTTGTCCCACAGATATCCAGAACCCTCTAAGAAGTTCCTGCTTTATATTTTTTCTCAGAATGTGGTTAGAGACATGACCCTGCCGTGTACCAGCTGAGAGACTCTAAATCCAGTGACAAGTCTG |
| E1 | TC15P3_pool3_5 | GAGATCATGTCCTAACCCTGATCCTCTTGTCCCACAGATATCCAGAACCCTCTAAGAAGTTCCTGCTAATATATTTTTCTGAGAATGTGGTTAGAGACATGACCCTGCCGTGTACCAGCTGAGAGACTCTAAATCCAGTGACAAGTCTG |
| F1 | TC15P3_pool3_6 | GAGATCATGTCCTAACCCTGATCCTCTTGTCCCACAGATATCCAGAACCCTCTAAGAAGTTCCTGCTTTTAAATATTTCTTAGAATGTGGTTAGAGACATGACCCTGCCGTGTACCAGCTGAGAGACTCTAAATCCAGTGACAAGTCTG |

**Supplementary Table 3. Sequences of the pool of ssODN with spacer lengths of 13 bp (from left to right: purple=TRAC left homology arm; pink= STAT3 left binding site; black= barcode; red= targeted sequence; orange= STAT3 right binding site; green TRAC right homology arm).**

| Well Position | Name | Sequence |
| --- | --- | --- |
| A1 | TC13P1_pool1_1 | GAGATCATGTCCTAACCCTGATCCTCTTGTCCCACAGATATCCAGAACCCTCTAAGAAGTTCCTGCTTTAATAAATCAAAGAATGTGGTTAGAGACATGACCCTGCCGTGTACCAGCTGAGAGACTCTAAATCCAGTGACAAGTCTG |
| B1 | TC13P1_pool1_2 | GAGATCATGTCCTAACCCTGATCCTCTTGTCCCACAGATATCCAGAACCCTCTAAGAAGTTCCTGCTTAATTTAATCACTGAATGTGGTTAGAGACATGACCCTGCCGTGTACCAGCTGAGAGACTCTAAATCCAGTGACAAGTCTG |
| C1 | TC13P1_pool1_3 | GAGATCATGTCCTAACCCTGATCCTCTTGTCCCACAGATATCCAGAACCCTCTAAGAAGTTCCTGCTAATTTTAATCAGAGAATGTGGTTAGAGACATGACCCTGCCGTGTACCAGCTGAGAGACTCTAAATCCAGTGACAAGTCTG |
| D1 | TC13P1_pool1_4 | GAGATCATGTCCTAACCCTGATCCTCTTGTCCCACAGATATCCAGAACCCTCTAAGAAGTTCCTGCTTTATATAATCATAGAATGTGGTTAGAGACATGACCCTGCCGTGTACCAGCTGAGAGACTCTAAATCCAGTGACAAGTCTG |
| E1 | TC13P1_pool1_5 | GAGATCATGTCCTAACCCTGATCCTCTTGTCCCACAGATATCCAGAACCCTCTAAGAAGTTCCTGCTAATTATAATCCATGAATGTGGTTAGAGACATGACCCTGCCGTGTACCAGCTGAGAGACTCTAAATCCAGTGACAAGTCTG |
| F1 | TC13P1_pool1_6 | GAGATCATGTCCTAACCCTGATCCTCTTGTCCCACAGATATCCAGAACCCTCTAAGAAGTTCCTGCTTTTTAAAATCCCAGAATGTGGTTAGAGACATGACCCTGCCGTGTACCAGCTGAGAGACTCTAAATCCAGTGACAAGTCTG |
| G1 | TC13P1_pool1_7 | GAGATCATGTCCTAACCCTGATCCTCTTGTCCCACAGATATCCAGAACCCTCTAAGAAGTTCCTGCTATTTATAATCCGAGAATGTGGTTAGAGACATGACCCTGCCGTGTACCAGCTGAGAGACTCTAAATCCAGTGACAAGTCTG |
| H1 | TC13P1_pool1_8 | GAGATCATGTCCTAACCCTGATCCTCTTGTCCCACAGATATCCAGAACCCTCTAAGAAGTTCCTGCTAAATTAAATCCTTGAATGTGGTTAGAGACATGACCCTGCCGTGTACCAGCTGAGAGACTCTAAATCCAGTGACAAGTCTG |
| A2 | TC13P1_pool1_9 | GAGATCATGTCCTAACCCTGATCCTCTTGTCCCACAGATATCCAGAACCCTCTAAGAAGTTCCTGCTATAAAAAATCGAAGAATGTGGTTAGAGACATGACCCTGCCGTGTACCAGCTGAGAGACTCTAAATCCAGTGACAAGTCTG |
| B2 | TC13P1_pool1_10 | GAGATCATGTCCTAACCCTGATCCTCTTGTCCCACAGATATCCAGAACCCTCTAAGAAGTTCCTGCTTATAATAATCGCTGAATGTGGTTAGAGACATGACCCTGCCGTGTACCAGCTGAGAGACTCTAAATCCAGTGACAAGTCTG |
| C2 | TC13P1_pool1_11 | GAGATCATGTCCTAACCCTGATCCTCTTGTCCCACAGATATCCAGAACCCTCTAAGAAGTTCCTGCTAAAAATAATCGGTGAATGTGGTTAGAGACATGACCCTGCCGTGTACCAGCTGAGAGACTCTAAATCCAGTGACAAGTCTG |
| D2 | TC13P1_pool1_12 | GAGATCATGTCCTAACCCTGATCCTCTTGTCCCACAGATATCCAGAACCCTCTAAGAAGTTCCTGCTTTTATTAATCGTAGAATGTGGTTAGAGACATGACCCTGCCGTGTACCAGCTGAGAGACTCTAAATCCAGTGACAAGTCTG |
| E2 | TC13P1_pool1_13 | GAGATCATGTCCTAACCCTGATCCTCTTGTCCCACAGATATCCAGAACCCTCTAAGAAGTTCCTGCTAATAAAAATCTATGAATGTGGTTAGAGACATGACCCTGCCGTGTACCAGCTGAGAGACTCTAAATCCAGTGACAAGTCTG |
| F2 | TC13P1_pool1_14 | GAGATCATGTCCTAACCCTGATCCTCTTGTCCCACAGATATCCAGAACCCTCTAAGAAGTTCCTGCTTATTTAAATCTCAGAATGTGGTTAGAGACATGACCCTGCCGTGTACCAGCTGAGAGACTCTAAATCCAGTGACAAGTCTG |
| G2 | TC13P1_pool1_15 | GAGATCATGTCCTAACCCTGATCCTCTTGTCCCACAGATATCCAGAACCCTCTAAGAAGTTCCTGCTTATTAAAATCTGTGAATGTGGTTAGAGACATGACCCTGCCGTGTACCAGCTGAGAGACTCTAAATCCAGTGACAAGTCTG |
| H2 | TC13P1_pool1_16 | GAGATCATGTCCTAACCCTGATCCTCTTGTCCCACAGATATCCAGAACCCTCTAAGAAGTTCCTGCTTTTATAAATCTTTGAATGTGGTTAGAGACATGACCCTGCCGTGTACCAGCTGAGAGACTCTAAATCCAGTGACAAGTCTG |
| A3 | TC13P1_pool1_17 | GAGATCATGTCCTAACCCTGATCCTCTTGTCCCACAGATATCCAGAACCCTCTAAGAAGTTCCTGCTTATTATACTCAAAGAATGTGGTTAGAGACATGACCCTGCCGTGTACCAGCTGAGAGACTCTAAATCCAGTGACAAGTCTG |
| B3 | TC13P1_pool1_18 | GAGATCATGTCCTAACCCTGATCCTCTTGTCCCACAGATATCCAGAACCCTCTAAGAAGTTCCTGCTATATATACTCACTGAATGTGGTTAGAGACATGACCCTGCCGTGTACCAGCTGAGAGACTCTAAATCCAGTGACAAGTCTG |
| C3 | TC13P1_pool1_19 | GAGATCATGTCCTAACCCTGATCCTCTTGTCCCACAGATATCCAGAACCCTCTAAGAAGTTCCTGCTATATTTACTCAGAGAATGTGGTTAGAGACATGACCCTGCCGTGTACCAGCTGAGAGACTCTAAATCCAGTGACAAGTCTG |
| D3 | TC13P1_pool1_20 | GAGATCATGTCCTAACCCTGATCCTCTTGTCCCACAGATATCCAGAACCCTCTAAGAAGTTCCTGCTATTATAACTCATAGAATGTGGTTAGAGACATGACCCTGCCGTGTACCAGCTGAGAGACTCTAAATCCAGTGACAAGTCTG |
| E3 | TC13P1_pool1_21 | GAGATCATGTCCTAACCCTGATCCTCTTGTCCCACAGATATCCAGAACCCTCTAAGAAGTTCCTGCTAATAATACTCCAAGAATGTGGTTAGAGACATGACCCTGCCGTGTACCAGCTGAGAGACTCTAAATCCAGTGACAAGTCTG |
| F3 | TC13P1_pool1_22 | GAGATCATGTCCTAACCCTGATCCTCTTGTCCCACAGATATCCAGAACCCTCTAAGAAGTTCCTGCTATAATTACTCCCTGAATGTGGTTAGAGACATGACCCTGCCGTGTACCAGCTGAGAGACTCTAAATCCAGTGACAAGTCTG |
| G3 | TC13P1_pool1_23 | GAGATCATGTCCTAACCCTGATCCTCTTGTCCCACAGATATCCAGAACCCTCTAAGAAGTTCCTGCTAAATATACTCCGAGAATGTGGTTAGAGACATGACCCTGCCGTGTACCAGCTGAGAGACTCTAAATCCAGTGACAAGTCTG |
| H3 | TC13P1_pool1_24 | GAGATCATGTCCTAACCCTGATCCTCTTGTCCCACAGATATCCAGAACCCTCTAAGAAGTTCCTGCTAAAATAACTCCTAGAATGTGGTTAGAGACATGACCCTGCCGTGTACCAGCTGAGAGACTCTAAATCCAGTGACAAGTCTG |
| A4 | TC13P1_pool1_25 | GAGATCATGTCCTAACCCTGATCCTCTTGTCCCACAGATATCCAGAACCCTCTAAGAAGTTCCTGCTATTTTAACTCGATGAATGTGGTTAGAGACATGACCCTGCCGTGTACCAGCTGAGAGACTCTAAATCCAGTGACAAGTCTG |
| B4 | TC13P1_pool1_26 | GAGATCATGTCCTAACCCTGATCCTCTTGTCCCACAGATATCCAGAACCCTCTAAGAAGTTCCTGCTTTTTATACTCGCTGAATGTGGTTAGAGACATGACCCTGCCGTGTACCAGCTGAGAGACTCTAAATCCAGTGACAAGTCTG |
| C4 | TC13P1_pool1_27 | GAGATCATGTCCTAACCCTGATCCTCTTGTCCCACAGATATCCAGAACCCTCTAAGAAGTTCCTGCTTAAAATACTCGGAGAATGTGGTTAGAGACATGACCCTGCCGTGTACCAGCTGAGAGACTCTAAATCCAGTGACAAGTCTG |
| D4 | TC13P1_pool1_28 | GAGATCATGTCCTAACCCTGATCCTCTTGTCCCACAGATATCCAGAACCCTCTAAGAAGTTCCTGCTATTAATACTCGTTGAATGTGGTTAGAGACATGACCCTGCCGTGTACCAGCTGAGAGACTCTAAATCCAGTGACAAGTCTG |
| E4 | TC13P1_pool1_29 | GAGATCATGTCCTAACCCTGATCCTCTTGTCCCACAGATATCCAGAACCCTCTAAGAAGTTCCTGCTTAATAAACTCTAAGAATGTGGTTAGAGACATGACCCTGCCGTGTACCAGCTGAGAGACTCTAAATCCAGTGACAAGTCTG |
| F4 | TC13P1_pool1_30 | GAGATCATGTCCTAACCCTGATCCTCTTGTCCCACAGATATCCAGAACCCTCTAAGAAGTTCCTGCTTAAATAACTCTCTGAATGTGGTTAGAGACATGACCCTGCCGTGTACCAGCTGAGAGACTCTAAATCCAGTGACAAGTCTG |
| G4 | TC13P1_pool1_31 | GAGATCATGTCCTAACCCTGATCCTCTTGTCCCACAGATATCCAGAACCCTCTAAGAAGTTCCTGCTTATAAAACTCTGAGAATGTGGTTAGAGACATGACCCTGCCGTGTACCAGCTGAGAGACTCTAAATCCAGTGACAAGTCTG |
| H4 | TC13P1_pool1_32 | GAGATCATGTCCTAACCCTGATCCTCTTGTCCCACAGATATCCAGAACCCTCTAAGAAGTTCCTGCTTTAAATACTCTTTGAATGTGGTTAGAGACATGACCCTGCCGTGTACCAGCTGAGAGACTCTAAATCCAGTGACAAGTCTG |
| A5 | TC13P1_pool1_33 | GAGATCATGTCCTAACCCTGATCCTCTTGTCCCACAGATATCCAGAACCCTCTAAGAAGTTCCTGCTAATATTAGTCAATGAATGTGGTTAGAGACATGACCCTGCCGTGTACCAGCTGAGAGACTCTAAATCCAGTGACAAGTCTG |
| B5 | TC13P1_pool1_34 | GAGATCATGTCCTAACCCTGATCCTCTTGTCCCACAGATATCCAGAACCCTCTAAGAAGTTCCTGCTTTATAAAGTCACTGAATGTGGTTAGAGACATGACCCTGCCGTGTACCAGCTGAGAGACTCTAAATCCAGTGACAAGTCTG |
|  |  |  |
| C5 | TC13P1_pool2_1 | GAGATCATGTCCTAACCCTGATCCTCTTGTCCCACAGATATCCAGAACCCTCTAAGAAGTTCCTGCTTTAATAAGTCAGAGAATGTGGTTAGAGACATGACCCTGCCGTGTACCAGCTGAGAGACTCTAAATCCAGTGACAAGTCTG |
| D5 | TC13P1_pool2_2 | GAGATCATGTCCTAACCCTGATCCTCTTGTCCCACAGATATCCAGAACCCTCTAAGAAGTTCCTGCTTAATTTAGTCATTGAATGTGGTTAGAGACATGACCCTGCCGTGTACCAGCTGAGAGACTCTAAATCCAGTGACAAGTCTG |
| E5 | TC13P1_pool2_3 | GAGATCATGTCCTAACCCTGATCCTCTTGTCCCACAGATATCCAGAACCCTCTAAGAAGTTCCTGCTAATTTTAGTCCAAGAATGTGGTTAGAGACATGACCCTGCCGTGTACCAGCTGAGAGACTCTAAATCCAGTGACAAGTCTG |
| F5 | TC13P1_pool2_4 | GAGATCATGTCCTAACCCTGATCCTCTTGTCCCACAGATATCCAGAACCCTCTAAGAAGTTCCTGCTTTATATAGTCCCAGAATGTGGTTAGAGACATGACCCTGCCGTGTACCAGCTGAGAGACTCTAAATCCAGTGACAAGTCTG |
| G5 | TC13P1_pool2_5 | GAGATCATGTCCTAACCCTGATCCTCTTGTCCCACAGATATCCAGAACCCTCTAAGAAGTTCCTGCTAATTATAGTCCGTGAATGTGGTTAGAGACATGACCCTGCCGTGTACCAGCTGAGAGACTCTAAATCCAGTGACAAGTCTG |
| H5 | TC13P1_pool2_6 | GAGATCATGTCCTAACCCTGATCCTCTTGTCCCACAGATATCCAGAACCCTCTAAGAAGTTCCTGCTTTTTAAAGTCCTAGAATGTGGTTAGAGACATGACCCTGCCGTGTACCAGCTGAGAGACTCTAAATCCAGTGACAAGTCTG |
| A6 | TC13P1_pool2_7 | GAGATCATGTCCTAACCCTGATCCTCTTGTCCCACAGATATCCAGAACCCTCTAAGAAGTTCCTGCTATTTATAGTCGAAGAATGTGGTTAGAGACATGACCCTGCCGTGTACCAGCTGAGAGACTCTAAATCCAGTGACAAGTCTG |
| B6 | TC13P1_pool2_8 | GAGATCATGTCCTAACCCTGATCCTCTTGTCCCACAGATATCCAGAACCCTCTAAGAAGTTCCTGCTAAATTAAGTCGCTGAATGTGGTTAGAGACATGACCCTGCCGTGTACCAGCTGAGAGACTCTAAATCCAGTGACAAGTCTG |
| C6 | TC13P1_pool2_9 | GAGATCATGTCCTAACCCTGATCCTCTTGTCCCACAGATATCCAGAACCCTCTAAGAAGTTCCTGCTATAAAAAGTCGGAGAATGTGGTTAGAGACATGACCCTGCCGTGTACCAGCTGAGAGACTCTAAATCCAGTGACAAGTCTG |
| D6 | TC13P1_pool2_10 | GAGATCATGTCCTAACCCTGATCCTCTTGTCCCACAGATATCCAGAACCCTCTAAGAAGTTCCTGCTTATAATAGTCGTTGAATGTGGTTAGAGACATGACCCTGCCGTGTACCAGCTGAGAGACTCTAAATCCAGTGACAAGTCTG |
| E6 | TC13P1_pool2_11 | GAGATCATGTCCTAACCCTGATCCTCTTGTCCCACAGATATCCAGAACCCTCTAAGAAGTTCCTGCTAAAAATAGTCTATGAATGTGGTTAGAGACATGACCCTGCCGTGTACCAGCTGAGAGACTCTAAATCCAGTGACAAGTCTG |
| F6 | TC13P1_pool2_12 | GAGATCATGTCCTAACCCTGATCCTCTTGTCCCACAGATATCCAGAACCCTCTAAGAAGTTCCTGCTTTTATTAGTCTCAGAATGTGGTTAGAGACATGACCCTGCCGTGTACCAGCTGAGAGACTCTAAATCCAGTGACAAGTCTG |
| G6 | TC13P1_pool2_13 | GAGATCATGTCCTAACCCTGATCCTCTTGTCCCACAGATATCCAGAACCCTCTAAGAAGTTCCTGCTAATAAAAGTCTGTGAATGTGGTTAGAGACATGACCCTGCCGTGTACCAGCTGAGAGACTCTAAATCCAGTGACAAGTCTG |
| H6 | TC13P1_pool2_14 | GAGATCATGTCCTAACCCTGATCCTCTTGTCCCACAGATATCCAGAACCCTCTAAGAAGTTCCTGCTTATTTAAGTCTTAGAATGTGGTTAGAGACATGACCCTGCCGTGTACCAGCTGAGAGACTCTAAATCCAGTGACAAGTCTG |
| A7 | TC13P1_pool2_15 | GAGATCATGTCCTAACCCTGATCCTCTTGTCCCACAGATATCCAGAACCCTCTAAGAAGTTCCTGCTTATTAAATTCAATGAATGTGGTTAGAGACATGACCCTGCCGTGTACCAGCTGAGAGACTCTAAATCCAGTGACAAGTCTG |
| B7 | TC13P1_pool2_16 | GAGATCATGTCCTAACCCTGATCCTCTTGTCCCACAGATATCCAGAACCCTCTAAGAAGTTCCTGCTTTTATAATTCACTGAATGTGGTTAGAGACATGACCCTGCCGTGTACCAGCTGAGAGACTCTAAATCCAGTGACAAGTCTG |
| C7 | TC13P1_pool2_17 | GAGATCATGTCCTAACCCTGATCCTCTTGTCCCACAGATATCCAGAACCCTCTAAGAAGTTCCTGCTTATTATATTCAGAGAATGTGGTTAGAGACATGACCCTGCCGTGTACCAGCTGAGAGACTCTAAATCCAGTGACAAGTCTG |
| D7 | TC13P1_pool2_18 | GAGATCATGTCCTAACCCTGATCCTCTTGTCCCACAGATATCCAGAACCCTCTAAGAAGTTCCTGCTATATATATTCATTGAATGTGGTTAGAGACATGACCCTGCCGTGTACCAGCTGAGAGACTCTAAATCCAGTGACAAGTCTG |
| E7 | TC13P1_pool2_19 | GAGATCATGTCCTAACCCTGATCCTCTTGTCCCACAGATATCCAGAACCCTCTAAGAAGTTCCTGCTATATTTATTCCAAGAATGTGGTTAGAGACATGACCCTGCCGTGTACCAGCTGAGAGACTCTAAATCCAGTGACAAGTCTG |
| F7 | TC13P1_pool2_20 | GAGATCATGTCCTAACCCTGATCCTCTTGTCCCACAGATATCCAGAACCCTCTAAGAAGTTCCTGCTATTATAATTCCCAGAATGTGGTTAGAGACATGACCCTGCCGTGTACCAGCTGAGAGACTCTAAATCCAGTGACAAGTCTG |
| G7 | TC13P1_pool2_21 | GAGATCATGTCCTAACCCTGATCCTCTTGTCCCACAGATATCCAGAACCCTCTAAGAAGTTCCTGCTAATAATATTCCGAGAATGTGGTTAGAGACATGACCCTGCCGTGTACCAGCTGAGAGACTCTAAATCCAGTGACAAGTCTG |
| H7 | TC13P1_pool2_22 | GAGATCATGTCCTAACCCTGATCCTCTTGTCCCACAGATATCCAGAACCCTCTAAGAAGTTCCTGCTATAATTATTCCTTGAATGTGGTTAGAGACATGACCCTGCCGTGTACCAGCTGAGAGACTCTAAATCCAGTGACAAGTCTG |
| A8 | TC13P1_pool2_23 | GAGATCATGTCCTAACCCTGATCCTCTTGTCCCACAGATATCCAGAACCCTCTAAGAAGTTCCTGCTAAATATATTCGAAGAATGTGGTTAGAGACATGACCCTGCCGTGTACCAGCTGAGAGACTCTAAATCCAGTGACAAGTCTG |
| B8 | TC13P1_pool2_24 | GAGATCATGTCCTAACCCTGATCCTCTTGTCCCACAGATATCCAGAACCCTCTAAGAAGTTCCTGCTAAAATAATTCGCAGAATGTGGTTAGAGACATGACCCTGCCGTGTACCAGCTGAGAGACTCTAAATCCAGTGACAAGTCTG |
| C8 | TC13P1_pool2_25 | GAGATCATGTCCTAACCCTGATCCTCTTGTCCCACAGATATCCAGAACCCTCTAAGAAGTTCCTGCTATTTTAATTCGGTGAATGTGGTTAGAGACATGACCCTGCCGTGTACCAGCTGAGAGACTCTAAATCCAGTGACAAGTCTG |
| D8 | TC13P1_pool2_26 | GAGATCATGTCCTAACCCTGATCCTCTTGTCCCACAGATATCCAGAACCCTCTAAGAAGTTCCTGCTTTTTATATTCGTTGAATGTGGTTAGAGACATGACCCTGCCGTGTACCAGCTGAGAGACTCTAAATCCAGTGACAAGTCTG |
| E8 | TC13P1_pool2_27 | GAGATCATGTCCTAACCCTGATCCTCTTGTCCCACAGATATCCAGAACCCTCTAAGAAGTTCCTGCTTAAAATATTCTAAGAATGTGGTTAGAGACATGACCCTGCCGTGTACCAGCTGAGAGACTCTAAATCCAGTGACAAGTCTG |
| F8 | TC13P1_pool2_28 | GAGATCATGTCCTAACCCTGATCCTCTTGTCCCACAGATATCCAGAACCCTCTAAGAAGTTCCTGCTATTAATATTCTCTGAATGTGGTTAGAGACATGACCCTGCCGTGTACCAGCTGAGAGACTCTAAATCCAGTGACAAGTCTG |
| G8 | TC13P1_pool2_29 | GAGATCATGTCCTAACCCTGATCCTCTTGTCCCACAGATATCCAGAACCCTCTAAGAAGTTCCTGCTTAATAAATTCTGAGAATGTGGTTAGAGACATGACCCTGCCGTGTACCAGCTGAGAGACTCTAAATCCAGTGACAAGTCTG |
| H8 | TC13P1_pool2_30 | GAGATCATGTCCTAACCCTGATCCTCTTGTCCCACAGATATCCAGAACCCTCTAAGAAGTTCCTGCTTAAATAATTCTTTGAATGTGGTTAGAGACATGACCCTGCCGTGTACCAGCTGAGAGACTCTAAATCCAGTGACAAGTCTG |
| A9 | TC13P1_pool2_31 | GAGATCATGTCCTAACCCTGATCCTCTTGTCCCACAGATATCCAGAACCCTCTAAGAAGTTCCTGCTTATAAACATCAAAGAATGTGGTTAGAGACATGACCCTGCCGTGTACCAGCTGAGAGACTCTAAATCCAGTGACAAGTCTG |
| B9 | TC13P1_pool2_32 | GAGATCATGTCCTAACCCTGATCCTCTTGTCCCACAGATATCCAGAACCCTCTAAGAAGTTCCTGCTTTAAATCATCACTGAATGTGGTTAGAGACATGACCCTGCCGTGTACCAGCTGAGAGACTCTAAATCCAGTGACAAGTCTG |
| C9 | TC13P1_pool2_33 | GAGATCATGTCCTAACCCTGATCCTCTTGTCCCACAGATATCCAGAACCCTCTAAGAAGTTCCTGCTAATATTCATCAGTGAATGTGGTTAGAGACATGACCCTGCCGTGTACCAGCTGAGAGACTCTAAATCCAGTGACAAGTCTG |
| D9 | TC13P1_pool2_34 | GAGATCATGTCCTAACCCTGATCCTCTTGTCCCACAGATATCCAGAACCCTCTAAGAAGTTCCTGCTTTATAACATCATTGAATGTGGTTAGAGACATGACCCTGCCGTGTACCAGCTGAGAGACTCTAAATCCAGTGACAAGTCTG |
|  |  |  |
| A1 | TC13P2_pool3_1 | GAGATCATGTCCTAACCCTGATCCTCTTGTCCCACAGATATCCAGAACCCTCTAAGAAGTTCCTGCTTTAATACATCCAAGAATGTGGTTAGAGACATGACCCTGCCGTGTACCAGCTGAGAGACTCTAAATCCAGTGACAAGTCTG |
| B1 | TC13P2_pool3_2 | GAGATCATGTCCTAACCCTGATCCTCTTGTCCCACAGATATCCAGAACCCTCTAAGAAGTTCCTGCTTAATTTCATCCCTGAATGTGGTTAGAGACATGACCCTGCCGTGTACCAGCTGAGAGACTCTAAATCCAGTGACAAGTCTG |
| C1 | TC13P2_pool3_3 | GAGATCATGTCCTAACCCTGATCCTCTTGTCCCACAGATATCCAGAACCCTCTAAGAAGTTCCTGCTAATTTTCATCCGAGAATGTGGTTAGAGACATGACCCTGCCGTGTACCAGCTGAGAGACTCTAAATCCAGTGACAAGTCTG |
| D1 | TC13P2_pool3_4 | GAGATCATGTCCTAACCCTGATCCTCTTGTCCCACAGATATCCAGAACCCTCTAAGAAGTTCCTGCTTTATATCATCCTAGAATGTGGTTAGAGACATGACCCTGCCGTGTACCAGCTGAGAGACTCTAAATCCAGTGACAAGTCTG |
| E1 | TC13P2_pool3_5 | GAGATCATGTCCTAACCCTGATCCTCTTGTCCCACAGATATCCAGAACCCTCTAAGAAGTTCCTGCTAATTATCATCGATGAATGTGGTTAGAGACATGACCCTGCCGTGTACCAGCTGAGAGACTCTAAATCCAGTGACAAGTCTG |
| F1 | TC13P2_pool3_6 | GAGATCATGTCCTAACCCTGATCCTCTTGTCCCACAGATATCCAGAACCCTCTAAGAAGTTCCTGCTTTTTAACATCGCAGAATGTGGTTAGAGACATGACCCTGCCGTGTACCAGCTGAGAGACTCTAAATCCAGTGACAAGTCTG |
| G1 | TC13P2_pool3_7 | GAGATCATGTCCTAACCCTGATCCTCTTGTCCCACAGATATCCAGAACCCTCTAAGAAGTTCCTGCTATTTATCATCGGAGAATGTGGTTAGAGACATGACCCTGCCGTGTACCAGCTGAGAGACTCTAAATCCAGTGACAAGTCTG |
| H1 | TC13P2_pool3_8 | GAGATCATGTCCTAACCCTGATCCTCTTGTCCCACAGATATCCAGAACCCTCTAAGAAGTTCCTGCTAAATTACATCGTTGAATGTGGTTAGAGACATGACCCTGCCGTGTACCAGCTGAGAGACTCTAAATCCAGTGACAAGTCTG |
| A2 | TC13P2_pool3_9 | GAGATCATGTCCTAACCCTGATCCTCTTGTCCCACAGATATCCAGAACCCTCTAAGAAGTTCCTGCTATAAAACATCTAAGAATGTGGTTAGAGACATGACCCTGCCGTGTACCAGCTGAGAGACTCTAAATCCAGTGACAAGTCTG |
| B2 | TC13P2_pool3_10 | GAGATCATGTCCTAACCCTGATCCTCTTGTCCCACAGATATCCAGAACCCTCTAAGAAGTTCCTGCTTATAATCATCTCTGAATGTGGTTAGAGACATGACCCTGCCGTGTACCAGCTGAGAGACTCTAAATCCAGTGACAAGTCTG |
| C2 | TC13P2_pool3_11 | GAGATCATGTCCTAACCCTGATCCTCTTGTCCCACAGATATCCAGAACCCTCTAAGAAGTTCCTGCTAAAAATCATCTGTGAATGTGGTTAGAGACATGACCCTGCCGTGTACCAGCTGAGAGACTCTAAATCCAGTGACAAGTCTG |
| D2 | TC13P2_pool3_12 | GAGATCATGTCCTAACCCTGATCCTCTTGTCCCACAGATATCCAGAACCCTCTAAGAAGTTCCTGCTTTTATTCATCTTAGAATGTGGTTAGAGACATGACCCTGCCGTGTACCAGCTGAGAGACTCTAAATCCAGTGACAAGTCTG |
| E2 | TC13P2_pool3_13 | GAGATCATGTCCTAACCCTGATCCTCTTGTCCCACAGATATCCAGAACCCTCTAAGAAGTTCCTGCTAATAAACCTCAATGAATGTGGTTAGAGACATGACCCTGCCGTGTACCAGCTGAGAGACTCTAAATCCAGTGACAAGTCTG |
| F2 | TC13P2_pool3_14 | GAGATCATGTCCTAACCCTGATCCTCTTGTCCCACAGATATCCAGAACCCTCTAAGAAGTTCCTGCTTATTTACCTCACAGAATGTGGTTAGAGACATGACCCTGCCGTGTACCAGCTGAGAGACTCTAAATCCAGTGACAAGTCTG |
| G2 | TC13P2_pool3_15 | GAGATCATGTCCTAACCCTGATCCTCTTGTCCCACAGATATCCAGAACCCTCTAAGAAGTTCCTGCTTATTAACCTCAGTGAATGTGGTTAGAGACATGACCCTGCCGTGTACCAGCTGAGAGACTCTAAATCCAGTGACAAGTCTG |
| H2 | TC13P2_pool3_16 | GAGATCATGTCCTAACCCTGATCCTCTTGTCCCACAGATATCCAGAACCCTCTAAGAAGTTCCTGCTTTTATACCTCATTGAATGTGGTTAGAGACATGACCCTGCCGTGTACCAGCTGAGAGACTCTAAATCCAGTGACAAGTCTG |
| A3 | TC13P2_pool3_17 | GAGATCATGTCCTAACCCTGATCCTCTTGTCCCACAGATATCCAGAACCCTCTAAGAAGTTCCTGCTTATTATCCTCCAAGAATGTGGTTAGAGACATGACCCTGCCGTGTACCAGCTGAGAGACTCTAAATCCAGTGACAAGTCTG |
| B3 | TC13P2_pool3_18 | GAGATCATGTCCTAACCCTGATCCTCTTGTCCCACAGATATCCAGAACCCTCTAAGAAGTTCCTGCTATATATCCTCCCTGAATGTGGTTAGAGACATGACCCTGCCGTGTACCAGCTGAGAGACTCTAAATCCAGTGACAAGTCTG |
| C3 | TC13P2_pool3_19 | GAGATCATGTCCTAACCCTGATCCTCTTGTCCCACAGATATCCAGAACCCTCTAAGAAGTTCCTGCTATATTTCCTCCGAGAATGTGGTTAGAGACATGACCCTGCCGTGTACCAGCTGAGAGACTCTAAATCCAGTGACAAGTCTG |
| D3 | TC13P2_pool3_20 | GAGATCATGTCCTAACCCTGATCCTCTTGTCCCACAGATATCCAGAACCCTCTAAGAAGTTCCTGCTATTATACCTCCTAGAATGTGGTTAGAGACATGACCCTGCCGTGTACCAGCTGAGAGACTCTAAATCCAGTGACAAGTCTG |
| E3 | TC13P2_pool3_21 | GAGATCATGTCCTAACCCTGATCCTCTTGTCCCACAGATATCCAGAACCCTCTAAGAAGTTCCTGCTAATAATCCTCGAAGAATGTGGTTAGAGACATGACCCTGCCGTGTACCAGCTGAGAGACTCTAAATCCAGTGACAAGTCTG |
| F3 | TC13P2_pool3_22 | GAGATCATGTCCTAACCCTGATCCTCTTGTCCCACAGATATCCAGAACCCTCTAAGAAGTTCCTGCTATAATTCCTCGCTGAATGTGGTTAGAGACATGACCCTGCCGTGTACCAGCTGAGAGACTCTAAATCCAGTGACAAGTCTG |
| G3 | TC13P2_pool3_23 | GAGATCATGTCCTAACCCTGATCCTCTTGTCCCACAGATATCCAGAACCCTCTAAGAAGTTCCTGCTAAATATCCTCGGAGAATGTGGTTAGAGACATGACCCTGCCGTGTACCAGCTGAGAGACTCTAAATCCAGTGACAAGTCTG |
| H3 | TC13P2_pool3_24 | GAGATCATGTCCTAACCCTGATCCTCTTGTCCCACAGATATCCAGAACCCTCTAAGAAGTTCCTGCTAAAATACCTCGTAGAATGTGGTTAGAGACATGACCCTGCCGTGTACCAGCTGAGAGACTCTAAATCCAGTGACAAGTCTG |
| A4 | TC13P2_pool3_25 | GAGATCATGTCCTAACCCTGATCCTCTTGTCCCACAGATATCCAGAACCCTCTAAGAAGTTCCTGCTATTTTACCTCTATGAATGTGGTTAGAGACATGACCCTGCCGTGTACCAGCTGAGAGACTCTAAATCCAGTGACAAGTCTG |
| B4 | TC13P2_pool3_26 | GAGATCATGTCCTAACCCTGATCCTCTTGTCCCACAGATATCCAGAACCCTCTAAGAAGTTCCTGCTTTTTATCCTCTCTGAATGTGGTTAGAGACATGACCCTGCCGTGTACCAGCTGAGAGACTCTAAATCCAGTGACAAGTCTG |
| C4 | TC13P2_pool3_27 | GAGATCATGTCCTAACCCTGATCCTCTTGTCCCACAGATATCCAGAACCCTCTAAGAAGTTCCTGCTTAAAATCCTCTGAGAATGTGGTTAGAGACATGACCCTGCCGTGTACCAGCTGAGAGACTCTAAATCCAGTGACAAGTCTG |
| D4 | TC13P2_pool3_28 | GAGATCATGTCCTAACCCTGATCCTCTTGTCCCACAGATATCCAGAACCCTCTAAGAAGTTCCTGCTATTAATCCTCTTTGAATGTGGTTAGAGACATGACCCTGCCGTGTACCAGCTGAGAGACTCTAAATCCAGTGACAAGTCTG |
| E4 | TC13P2_pool3_29 | GAGATCATGTCCTAACCCTGATCCTCTTGTCCCACAGATATCCAGAACCCTCTAAGAAGTTCCTGCTTAATAACGTCAAAGAATGTGGTTAGAGACATGACCCTGCCGTGTACCAGCTGAGAGACTCTAAATCCAGTGACAAGTCTG |
| F4 | TC13P2_pool3_30 | GAGATCATGTCCTAACCCTGATCCTCTTGTCCCACAGATATCCAGAACCCTCTAAGAAGTTCCTGCTTAAATACGTCACTGAATGTGGTTAGAGACATGACCCTGCCGTGTACCAGCTGAGAGACTCTAAATCCAGTGACAAGTCTG |
| G4 | TC13P2_pool3_31 | GAGATCATGTCCTAACCCTGATCCTCTTGTCCCACAGATATCCAGAACCCTCTAAGAAGTTCCTGCTTATAAACGTCAGAGAATGTGGTTAGAGACATGACCCTGCCGTGTACCAGCTGAGAGACTCTAAATCCAGTGACAAGTCTG |
| H4 | TC13P2_pool3_32 | GAGATCATGTCCTAACCCTGATCCTCTTGTCCCACAGATATCCAGAACCCTCTAAGAAGTTCCTGCTTTAAATCGTCATTGAATGTGGTTAGAGACATGACCCTGCCGTGTACCAGCTGAGAGACTCTAAATCCAGTGACAAGTCTG |
| A5 | TC13P2_pool3_33 | GAGATCATGTCCTAACCCTGATCCTCTTGTCCCACAGATATCCAGAACCCTCTAAGAAGTTCCTGCTAATATTCGTCCATGAATGTGGTTAGAGACATGACCCTGCCGTGTACCAGCTGAGAGACTCTAAATCCAGTGACAAGTCTG |
| B5 | TC13P2_pool3_34 | GAGATCATGTCCTAACCCTGATCCTCTTGTCCCACAGATATCCAGAACCCTCTAAGAAGTTCCTGCTTTATAACGTCCCTGAATGTGGTTAGAGACATGACCCTGCCGTGTACCAGCTGAGAGACTCTAAATCCAGTGACAAGTCTG |
|  |  |  |
| C5 | TC13P2_pool4_1 | GAGATCATGTCCTAACCCTGATCCTCTTGTCCCACAGATATCCAGAACCCTCTAAGAAGTTCCTGCTTTAATACGTCCGAGAATGTGGTTAGAGACATGACCCTGCCGTGTACCAGCTGAGAGACTCTAAATCCAGTGACAAGTCTG |
| D5 | TC13P2_pool4_2 | GAGATCATGTCCTAACCCTGATCCTCTTGTCCCACAGATATCCAGAACCCTCTAAGAAGTTCCTGCTTAATTTCGTCCTTGAATGTGGTTAGAGACATGACCCTGCCGTGTACCAGCTGAGAGACTCTAAATCCAGTGACAAGTCTG |
| E5 | TC13P2_pool4_3 | GAGATCATGTCCTAACCCTGATCCTCTTGTCCCACAGATATCCAGAACCCTCTAAGAAGTTCCTGCTAATTTTCGTCGAAGAATGTGGTTAGAGACATGACCCTGCCGTGTACCAGCTGAGAGACTCTAAATCCAGTGACAAGTCTG |
| F5 | TC13P2_pool4_4 | GAGATCATGTCCTAACCCTGATCCTCTTGTCCCACAGATATCCAGAACCCTCTAAGAAGTTCCTGCTTTATATCGTCGCAGAATGTGGTTAGAGACATGACCCTGCCGTGTACCAGCTGAGAGACTCTAAATCCAGTGACAAGTCTG |
| G5 | TC13P2_pool4_5 | GAGATCATGTCCTAACCCTGATCCTCTTGTCCCACAGATATCCAGAACCCTCTAAGAAGTTCCTGCTAATTATCGTCGGTGAATGTGGTTAGAGACATGACCCTGCCGTGTACCAGCTGAGAGACTCTAAATCCAGTGACAAGTCTG |
| H5 | TC13P2_pool4_6 | GAGATCATGTCCTAACCCTGATCCTCTTGTCCCACAGATATCCAGAACCCTCTAAGAAGTTCCTGCTTTTTAACGTCGTAGAATGTGGTTAGAGACATGACCCTGCCGTGTACCAGCTGAGAGACTCTAAATCCAGTGACAAGTCTG |
| A6 | TC13P2_pool4_7 | GAGATCATGTCCTAACCCTGATCCTCTTGTCCCACAGATATCCAGAACCCTCTAAGAAGTTCCTGCTATTTATCGTCTAAGAATGTGGTTAGAGACATGACCCTGCCGTGTACCAGCTGAGAGACTCTAAATCCAGTGACAAGTCTG |
| B6 | TC13P2_pool4_8 | GAGATCATGTCCTAACCCTGATCCTCTTGTCCCACAGATATCCAGAACCCTCTAAGAAGTTCCTGCTAAATTACGTCTCTGAATGTGGTTAGAGACATGACCCTGCCGTGTACCAGCTGAGAGACTCTAAATCCAGTGACAAGTCTG |
| C6 | TC13P2_pool4_9 | GAGATCATGTCCTAACCCTGATCCTCTTGTCCCACAGATATCCAGAACCCTCTAAGAAGTTCCTGCTATAAAACGTCTGAGAATGTGGTTAGAGACATGACCCTGCCGTGTACCAGCTGAGAGACTCTAAATCCAGTGACAAGTCTG |
| D6 | TC13P2_pool4_10 | GAGATCATGTCCTAACCCTGATCCTCTTGTCCCACAGATATCCAGAACCCTCTAAGAAGTTCCTGCTTATAATCGTCTTTGAATGTGGTTAGAGACATGACCCTGCCGTGTACCAGCTGAGAGACTCTAAATCCAGTGACAAGTCTG |
| E6 | TC13P2_pool4_11 | GAGATCATGTCCTAACCCTGATCCTCTTGTCCCACAGATATCCAGAACCCTCTAAGAAGTTCCTGCTAAAAATCTTCAATGAATGTGGTTAGAGACATGACCCTGCCGTGTACCAGCTGAGAGACTCTAAATCCAGTGACAAGTCTG |
| F6 | TC13P2_pool4_12 | GAGATCATGTCCTAACCCTGATCCTCTTGTCCCACAGATATCCAGAACCCTCTAAGAAGTTCCTGCTTTTATTCTTCACAGAATGTGGTTAGAGACATGACCCTGCCGTGTACCAGCTGAGAGACTCTAAATCCAGTGACAAGTCTG |
| G6 | TC13P2_pool4_13 | GAGATCATGTCCTAACCCTGATCCTCTTGTCCCACAGATATCCAGAACCCTCTAAGAAGTTCCTGCTAATAAACTTCAGTGAATGTGGTTAGAGACATGACCCTGCCGTGTACCAGCTGAGAGACTCTAAATCCAGTGACAAGTCTG |
| H6 | TC13P2_pool4_14 | GAGATCATGTCCTAACCCTGATCCTCTTGTCCCACAGATATCCAGAACCCTCTAAGAAGTTCCTGCTTATTTACTTCATAGAATGTGGTTAGAGACATGACCCTGCCGTGTACCAGCTGAGAGACTCTAAATCCAGTGACAAGTCTG |
| A7 | TC13P2_pool4_15 | GAGATCATGTCCTAACCCTGATCCTCTTGTCCCACAGATATCCAGAACCCTCTAAGAAGTTCCTGCTTATTAACTTCCATGAATGTGGTTAGAGACATGACCCTGCCGTGTACCAGCTGAGAGACTCTAAATCCAGTGACAAGTCTG |
| B7 | TC13P2_pool4_16 | GAGATCATGTCCTAACCCTGATCCTCTTGTCCCACAGATATCCAGAACCCTCTAAGAAGTTCCTGCTTTTATACTTCCCTGAATGTGGTTAGAGACATGACCCTGCCGTGTACCAGCTGAGAGACTCTAAATCCAGTGACAAGTCTG |
| C7 | TC13P2_pool4_17 | GAGATCATGTCCTAACCCTGATCCTCTTGTCCCACAGATATCCAGAACCCTCTAAGAAGTTCCTGCTTATTATCTTCCGAGAATGTGGTTAGAGACATGACCCTGCCGTGTACCAGCTGAGAGACTCTAAATCCAGTGACAAGTCTG |
| D7 | TC13P2_pool4_18 | GAGATCATGTCCTAACCCTGATCCTCTTGTCCCACAGATATCCAGAACCCTCTAAGAAGTTCCTGCTATATATCTTCCTTGAATGTGGTTAGAGACATGACCCTGCCGTGTACCAGCTGAGAGACTCTAAATCCAGTGACAAGTCTG |
| E7 | TC13P2_pool4_19 | GAGATCATGTCCTAACCCTGATCCTCTTGTCCCACAGATATCCAGAACCCTCTAAGAAGTTCCTGCTATATTTCTTCGAAGAATGTGGTTAGAGACATGACCCTGCCGTGTACCAGCTGAGAGACTCTAAATCCAGTGACAAGTCTG |
| F7 | TC13P2_pool4_20 | GAGATCATGTCCTAACCCTGATCCTCTTGTCCCACAGATATCCAGAACCCTCTAAGAAGTTCCTGCTATTATACTTCGCAGAATGTGGTTAGAGACATGACCCTGCCGTGTACCAGCTGAGAGACTCTAAATCCAGTGACAAGTCTG |
| G7 | TC13P2_pool4_21 | GAGATCATGTCCTAACCCTGATCCTCTTGTCCCACAGATATCCAGAACCCTCTAAGAAGTTCCTGCTAATAATCTTCGGAGAATGTGGTTAGAGACATGACCCTGCCGTGTACCAGCTGAGAGACTCTAAATCCAGTGACAAGTCTG |
| H7 | TC13P2_pool4_22 | GAGATCATGTCCTAACCCTGATCCTCTTGTCCCACAGATATCCAGAACCCTCTAAGAAGTTCCTGCTATAATTCTTCGTTGAATGTGGTTAGAGACATGACCCTGCCGTGTACCAGCTGAGAGACTCTAAATCCAGTGACAAGTCTG |
| A8 | TC13P2_pool4_23 | GAGATCATGTCCTAACCCTGATCCTCTTGTCCCACAGATATCCAGAACCCTCTAAGAAGTTCCTGCTAAATATCTTCTAAGAATGTGGTTAGAGACATGACCCTGCCGTGTACCAGCTGAGAGACTCTAAATCCAGTGACAAGTCTG |
| B8 | TC13P2_pool4_24 | GAGATCATGTCCTAACCCTGATCCTCTTGTCCCACAGATATCCAGAACCCTCTAAGAAGTTCCTGCTAAAATACTTCTCAGAATGTGGTTAGAGACATGACCCTGCCGTGTACCAGCTGAGAGACTCTAAATCCAGTGACAAGTCTG |
| C8 | TC13P2_pool4_25 | GAGATCATGTCCTAACCCTGATCCTCTTGTCCCACAGATATCCAGAACCCTCTAAGAAGTTCCTGCTATTTTACTTCTGTGAATGTGGTTAGAGACATGACCCTGCCGTGTACCAGCTGAGAGACTCTAAATCCAGTGACAAGTCTG |
| D8 | TC13P2_pool4_26 | GAGATCATGTCCTAACCCTGATCCTCTTGTCCCACAGATATCCAGAACCCTCTAAGAAGTTCCTGCTTTTTATCTTCTTTGAATGTGGTTAGAGACATGACCCTGCCGTGTACCAGCTGAGAGACTCTAAATCCAGTGACAAGTCTG |
| E8 | TC13P2_pool4_27 | GAGATCATGTCCTAACCCTGATCCTCTTGTCCCACAGATATCCAGAACCCTCTAAGAAGTTCCTGCTTAAAATGATCAAAGAATGTGGTTAGAGACATGACCCTGCCGTGTACCAGCTGAGAGACTCTAAATCCAGTGACAAGTCTG |
| F8 | TC13P2_pool4_28 | GAGATCATGTCCTAACCCTGATCCTCTTGTCCCACAGATATCCAGAACCCTCTAAGAAGTTCCTGCTATTAATGATCACTGAATGTGGTTAGAGACATGACCCTGCCGTGTACCAGCTGAGAGACTCTAAATCCAGTGACAAGTCTG |
| G8 | TC13P2_pool4_29 | GAGATCATGTCCTAACCCTGATCCTCTTGTCCCACAGATATCCAGAACCCTCTAAGAAGTTCCTGCTTAATAAGATCAGAGAATGTGGTTAGAGACATGACCCTGCCGTGTACCAGCTGAGAGACTCTAAATCCAGTGACAAGTCTG |
| H8 | TC13P2_pool4_30 | GAGATCATGTCCTAACCCTGATCCTCTTGTCCCACAGATATCCAGAACCCTCTAAGAAGTTCCTGCTTAAATAGATCATTGAATGTGGTTAGAGACATGACCCTGCCGTGTACCAGCTGAGAGACTCTAAATCCAGTGACAAGTCTG |
| A9 | TC13P2_pool4_31 | GAGATCATGTCCTAACCCTGATCCTCTTGTCCCACAGATATCCAGAACCCTCTAAGAAGTTCCTGCTTATAAAGATCCAAGAATGTGGTTAGAGACATGACCCTGCCGTGTACCAGCTGAGAGACTCTAAATCCAGTGACAAGTCTG |
| B9 | TC13P2_pool4_32 | GAGATCATGTCCTAACCCTGATCCTCTTGTCCCACAGATATCCAGAACCCTCTAAGAAGTTCCTGCTTTAAATGATCCCTGAATGTGGTTAGAGACATGACCCTGCCGTGTACCAGCTGAGAGACTCTAAATCCAGTGACAAGTCTG |
| C9 | TC13P2_pool4_33 | GAGATCATGTCCTAACCCTGATCCTCTTGTCCCACAGATATCCAGAACCCTCTAAGAAGTTCCTGCTAATATTGATCCGTGAATGTGGTTAGAGACATGACCCTGCCGTGTACCAGCTGAGAGACTCTAAATCCAGTGACAAGTCTG |
| D9 | TC13P2_pool4_34 | GAGATCATGTCCTAACCCTGATCCTCTTGTCCCACAGATATCCAGAACCCTCTAAGAAGTTCCTGCTTTATAAGATCCTTGAATGTGGTTAGAGACATGACCCTGCCGTGTACCAGCTGAGAGACTCTAAATCCAGTGACAAGTCTG |
|  |  |  |
| A1 | TC13P3_pool5_1 | GAGATCATGTCCTAACCCTGATCCTCTTGTCCCACAGATATCCAGAACCCTCTAAGAAGTTCCTGCTTTAATAGATCGAAGAATGTGGTTAGAGACATGACCCTGCCGTGTACCAGCTGAGAGACTCTAAATCCAGTGACAAGTCTG |
| B1 | TC13P3_pool5_2 | GAGATCATGTCCTAACCCTGATCCTCTTGTCCCACAGATATCCAGAACCCTCTAAGAAGTTCCTGCTTAATTTGATCGCTGAATGTGGTTAGAGACATGACCCTGCCGTGTACCAGCTGAGAGACTCTAAATCCAGTGACAAGTCTG |
| C1 | TC13P3_pool5_3 | GAGATCATGTCCTAACCCTGATCCTCTTGTCCCACAGATATCCAGAACCCTCTAAGAAGTTCCTGCTAATTTTGATCGGAGAATGTGGTTAGAGACATGACCCTGCCGTGTACCAGCTGAGAGACTCTAAATCCAGTGACAAGTCTG |
| D1 | TC13P3_pool5_4 | GAGATCATGTCCTAACCCTGATCCTCTTGTCCCACAGATATCCAGAACCCTCTAAGAAGTTCCTGCTTTATATGATCGTAGAATGTGGTTAGAGACATGACCCTGCCGTGTACCAGCTGAGAGACTCTAAATCCAGTGACAAGTCTG |
| E1 | TC13P3_pool5_5 | GAGATCATGTCCTAACCCTGATCCTCTTGTCCCACAGATATCCAGAACCCTCTAAGAAGTTCCTGCTAATTATGATCTATGAATGTGGTTAGAGACATGACCCTGCCGTGTACCAGCTGAGAGACTCTAAATCCAGTGACAAGTCTG |
| F1 | TC13P3_pool5_6 | GAGATCATGTCCTAACCCTGATCCTCTTGTCCCACAGATATCCAGAACCCTCTAAGAAGTTCCTGCTTTTTAAGATCTCAGAATGTGGTTAGAGACATGACCCTGCCGTGTACCAGCTGAGAGACTCTAAATCCAGTGACAAGTCTG |
| G1 | TC13P3_pool5_7 | GAGATCATGTCCTAACCCTGATCCTCTTGTCCCACAGATATCCAGAACCCTCTAAGAAGTTCCTGCTATTTATGATCTGAGAATGTGGTTAGAGACATGACCCTGCCGTGTACCAGCTGAGAGACTCTAAATCCAGTGACAAGTCTG |
| H1 | TC13P3_pool5_8 | GAGATCATGTCCTAACCCTGATCCTCTTGTCCCACAGATATCCAGAACCCTCTAAGAAGTTCCTGCTAAATTAGATCTTTGAATGTGGTTAGAGACATGACCCTGCCGTGTACCAGCTGAGAGACTCTAAATCCAGTGACAAGTCTG |
| A2 | TC13P3_pool5_9 | GAGATCATGTCCTAACCCTGATCCTCTTGTCCCACAGATATCCAGAACCCTCTAAGAAGTTCCTGCTATAAAAGCTCAAAGAATGTGGTTAGAGACATGACCCTGCCGTGTACCAGCTGAGAGACTCTAAATCCAGTGACAAGTCTG |
| B2 | TC13P3_pool5_10 | GAGATCATGTCCTAACCCTGATCCTCTTGTCCCACAGATATCCAGAACCCTCTAAGAAGTTCCTGCTTATAATGCTCACTGAATGTGGTTAGAGACATGACCCTGCCGTGTACCAGCTGAGAGACTCTAAATCCAGTGACAAGTCTG |
| C2 | TC13P3_pool5_11 | GAGATCATGTCCTAACCCTGATCCTCTTGTCCCACAGATATCCAGAACCCTCTAAGAAGTTCCTGCTAAAAATGCTCAGTGAATGTGGTTAGAGACATGACCCTGCCGTGTACCAGCTGAGAGACTCTAAATCCAGTGACAAGTCTG |
| D2 | TC13P3_pool5_12 | GAGATCATGTCCTAACCCTGATCCTCTTGTCCCACAGATATCCAGAACCCTCTAAGAAGTTCCTGCTTTTATTGCTCATAGAATGTGGTTAGAGACATGACCCTGCCGTGTACCAGCTGAGAGACTCTAAATCCAGTGACAAGTCTG |
| E2 | TC13P3_pool5_13 | GAGATCATGTCCTAACCCTGATCCTCTTGTCCCACAGATATCCAGAACCCTCTAAGAAGTTCCTGCTAATAAAGCTCCATGAATGTGGTTAGAGACATGACCCTGCCGTGTACCAGCTGAGAGACTCTAAATCCAGTGACAAGTCTG |
| F2 | TC13P3_pool5_14 | GAGATCATGTCCTAACCCTGATCCTCTTGTCCCACAGATATCCAGAACCCTCTAAGAAGTTCCTGCTTATTTAGCTCCCAGAATGTGGTTAGAGACATGACCCTGCCGTGTACCAGCTGAGAGACTCTAAATCCAGTGACAAGTCTG |
| G2 | TC13P3_pool5_15 | GAGATCATGTCCTAACCCTGATCCTCTTGTCCCACAGATATCCAGAACCCTCTAAGAAGTTCCTGCTTATTAAGCTCCGTGAATGTGGTTAGAGACATGACCCTGCCGTGTACCAGCTGAGAGACTCTAAATCCAGTGACAAGTCTG |
| H2 | TC13P3_pool5_16 | GAGATCATGTCCTAACCCTGATCCTCTTGTCCCACAGATATCCAGAACCCTCTAAGAAGTTCCTGCTTTTATAGCTCCTTGAATGTGGTTAGAGACATGACCCTGCCGTGTACCAGCTGAGAGACTCTAAATCCAGTGACAAGTCTG |
| A3 | TC13P3_pool5_17 | GAGATCATGTCCTAACCCTGATCCTCTTGTCCCACAGATATCCAGAACCCTCTAAGAAGTTCCTGCTTATTATGCTCGAAGAATGTGGTTAGAGACATGACCCTGCCGTGTACCAGCTGAGAGACTCTAAATCCAGTGACAAGTCTG |
| B3 | TC13P3_pool5_18 | GAGATCATGTCCTAACCCTGATCCTCTTGTCCCACAGATATCCAGAACCCTCTAAGAAGTTCCTGCTATATATGCTCGCTGAATGTGGTTAGAGACATGACCCTGCCGTGTACCAGCTGAGAGACTCTAAATCCAGTGACAAGTCTG |
| C3 | TC13P3_pool5_19 | GAGATCATGTCCTAACCCTGATCCTCTTGTCCCACAGATATCCAGAACCCTCTAAGAAGTTCCTGCTATATTTGCTCGGAGAATGTGGTTAGAGACATGACCCTGCCGTGTACCAGCTGAGAGACTCTAAATCCAGTGACAAGTCTG |
| D3 | TC13P3_pool5_20 | GAGATCATGTCCTAACCCTGATCCTCTTGTCCCACAGATATCCAGAACCCTCTAAGAAGTTCCTGCTATTATAGCTCGTAGAATGTGGTTAGAGACATGACCCTGCCGTGTACCAGCTGAGAGACTCTAAATCCAGTGACAAGTCTG |
| E3 | TC13P3_pool5_21 | GAGATCATGTCCTAACCCTGATCCTCTTGTCCCACAGATATCCAGAACCCTCTAAGAAGTTCCTGCTAATAATGCTCTAAGAATGTGGTTAGAGACATGACCCTGCCGTGTACCAGCTGAGAGACTCTAAATCCAGTGACAAGTCTG |
| F3 | TC13P3_pool5_22 | GAGATCATGTCCTAACCCTGATCCTCTTGTCCCACAGATATCCAGAACCCTCTAAGAAGTTCCTGCTATAATTGCTCTCTGAATGTGGTTAGAGACATGACCCTGCCGTGTACCAGCTGAGAGACTCTAAATCCAGTGACAAGTCTG |
| G3 | TC13P3_pool5_23 | GAGATCATGTCCTAACCCTGATCCTCTTGTCCCACAGATATCCAGAACCCTCTAAGAAGTTCCTGCTAAATATGCTCTGAGAATGTGGTTAGAGACATGACCCTGCCGTGTACCAGCTGAGAGACTCTAAATCCAGTGACAAGTCTG |
| H3 | TC13P3_pool5_24 | GAGATCATGTCCTAACCCTGATCCTCTTGTCCCACAGATATCCAGAACCCTCTAAGAAGTTCCTGCTAAAATAGCTCTTAGAATGTGGTTAGAGACATGACCCTGCCGTGTACCAGCTGAGAGACTCTAAATCCAGTGACAAGTCTG |
| A4 | TC13P3_pool5_25 | GAGATCATGTCCTAACCCTGATCCTCTTGTCCCACAGATATCCAGAACCCTCTAAGAAGTTCCTGCTATTTTAGGTCAATGAATGTGGTTAGAGACATGACCCTGCCGTGTACCAGCTGAGAGACTCTAAATCCAGTGACAAGTCTG |
| B4 | TC13P3_pool5_26 | GAGATCATGTCCTAACCCTGATCCTCTTGTCCCACAGATATCCAGAACCCTCTAAGAAGTTCCTGCTTTTTATGGTCACTGAATGTGGTTAGAGACATGACCCTGCCGTGTACCAGCTGAGAGACTCTAAATCCAGTGACAAGTCTG |
| C4 | TC13P3_pool5_27 | GAGATCATGTCCTAACCCTGATCCTCTTGTCCCACAGATATCCAGAACCCTCTAAGAAGTTCCTGCTTAAAATGGTCAGAGAATGTGGTTAGAGACATGACCCTGCCGTGTACCAGCTGAGAGACTCTAAATCCAGTGACAAGTCTG |
| D4 | TC13P3_pool5_28 | GAGATCATGTCCTAACCCTGATCCTCTTGTCCCACAGATATCCAGAACCCTCTAAGAAGTTCCTGCTATTAATGGTCATTGAATGTGGTTAGAGACATGACCCTGCCGTGTACCAGCTGAGAGACTCTAAATCCAGTGACAAGTCTG |
| E4 | TC13P3_pool5_29 | GAGATCATGTCCTAACCCTGATCCTCTTGTCCCACAGATATCCAGAACCCTCTAAGAAGTTCCTGCTTAATAAGGTCCAAGAATGTGGTTAGAGACATGACCCTGCCGTGTACCAGCTGAGAGACTCTAAATCCAGTGACAAGTCTG |
| F4 | TC13P3_pool5_30 | GAGATCATGTCCTAACCCTGATCCTCTTGTCCCACAGATATCCAGAACCCTCTAAGAAGTTCCTGCTTAAATAGGTCCCTGAATGTGGTTAGAGACATGACCCTGCCGTGTACCAGCTGAGAGACTCTAAATCCAGTGACAAGTCTG |
| G4 | TC13P3_pool5_31 | GAGATCATGTCCTAACCCTGATCCTCTTGTCCCACAGATATCCAGAACCCTCTAAGAAGTTCCTGCTTATAAAGGTCCGAGAATGTGGTTAGAGACATGACCCTGCCGTGTACCAGCTGAGAGACTCTAAATCCAGTGACAAGTCTG |
| H4 | TC13P3_pool5_32 | GAGATCATGTCCTAACCCTGATCCTCTTGTCCCACAGATATCCAGAACCCTCTAAGAAGTTCCTGCTTTAAATGGTCCTTGAATGTGGTTAGAGACATGACCCTGCCGTGTACCAGCTGAGAGACTCTAAATCCAGTGACAAGTCTG |
| A5 | TC13P3_pool5_33 | GAGATCATGTCCTAACCCTGATCCTCTTGTCCCACAGATATCCAGAACCCTCTAAGAAGTTCCTGCTAATATTGGTCGATGAATGTGGTTAGAGACATGACCCTGCCGTGTACCAGCTGAGAGACTCTAAATCCAGTGACAAGTCTG |
| B5 | TC13P3_pool5_34 | GAGATCATGTCCTAACCCTGATCCTCTTGTCCCACAGATATCCAGAACCCTCTAAGAAGTTCCTGCTTTATAAGGTCGCTGAATGTGGTTAGAGACATGACCCTGCCGTGTACCAGCTGAGAGACTCTAAATCCAGTGACAAGTCTG |
|  |  |  |
| C5 | TC13P3_pool6_1 | GAGATCATGTCCTAACCCTGATCCTCTTGTCCCACAGATATCCAGAACCCTCTAAGAAGTTCCTGCTTTAATAGGTCGGAGAATGTGGTTAGAGACATGACCCTGCCGTGTACCAGCTGAGAGACTCTAAATCCAGTGACAAGTCTG |
| D5 | TC13P3_pool6_2 | GAGATCATGTCCTAACCCTGATCCTCTTGTCCCACAGATATCCAGAACCCTCTAAGAAGTTCCTGCTTAATTTGGTCGTTGAATGTGGTTAGAGACATGACCCTGCCGTGTACCAGCTGAGAGACTCTAAATCCAGTGACAAGTCTG |
| E5 | TC13P3_pool6_3 | GAGATCATGTCCTAACCCTGATCCTCTTGTCCCACAGATATCCAGAACCCTCTAAGAAGTTCCTGCTAATTTTGGTCTAAGAATGTGGTTAGAGACATGACCCTGCCGTGTACCAGCTGAGAGACTCTAAATCCAGTGACAAGTCTG |
| F5 | TC13P3_pool6_4 | GAGATCATGTCCTAACCCTGATCCTCTTGTCCCACAGATATCCAGAACCCTCTAAGAAGTTCCTGCTTTATATGGTCTCAGAATGTGGTTAGAGACATGACCCTGCCGTGTACCAGCTGAGAGACTCTAAATCCAGTGACAAGTCTG |
| G5 | TC13P3_pool6_5 | GAGATCATGTCCTAACCCTGATCCTCTTGTCCCACAGATATCCAGAACCCTCTAAGAAGTTCCTGCTAATTATGGTCTGTGAATGTGGTTAGAGACATGACCCTGCCGTGTACCAGCTGAGAGACTCTAAATCCAGTGACAAGTCTG |
| H5 | TC13P3_pool6_6 | GAGATCATGTCCTAACCCTGATCCTCTTGTCCCACAGATATCCAGAACCCTCTAAGAAGTTCCTGCTTTTTAAGGTCTTAGAATGTGGTTAGAGACATGACCCTGCCGTGTACCAGCTGAGAGACTCTAAATCCAGTGACAAGTCTG |
| A6 | TC13P3_pool6_7 | GAGATCATGTCCTAACCCTGATCCTCTTGTCCCACAGATATCCAGAACCCTCTAAGAAGTTCCTGCTATTTATGTTCAAAGAATGTGGTTAGAGACATGACCCTGCCGTGTACCAGCTGAGAGACTCTAAATCCAGTGACAAGTCTG |
| B6 | TC13P3_pool6_8 | GAGATCATGTCCTAACCCTGATCCTCTTGTCCCACAGATATCCAGAACCCTCTAAGAAGTTCCTGCTAAATTAGTTCACTGAATGTGGTTAGAGACATGACCCTGCCGTGTACCAGCTGAGAGACTCTAAATCCAGTGACAAGTCTG |
| C6 | TC13P3_pool6_9 | GAGATCATGTCCTAACCCTGATCCTCTTGTCCCACAGATATCCAGAACCCTCTAAGAAGTTCCTGCTATAAAAGTTCAGAGAATGTGGTTAGAGACATGACCCTGCCGTGTACCAGCTGAGAGACTCTAAATCCAGTGACAAGTCTG |
| D6 | TC13P3_pool6_10 | GAGATCATGTCCTAACCCTGATCCTCTTGTCCCACAGATATCCAGAACCCTCTAAGAAGTTCCTGCTTATAATGTTCATTGAATGTGGTTAGAGACATGACCCTGCCGTGTACCAGCTGAGAGACTCTAAATCCAGTGACAAGTCTG |
| E6 | TC13P3_pool6_11 | GAGATCATGTCCTAACCCTGATCCTCTTGTCCCACAGATATCCAGAACCCTCTAAGAAGTTCCTGCTAAAAATGTTCCATGAATGTGGTTAGAGACATGACCCTGCCGTGTACCAGCTGAGAGACTCTAAATCCAGTGACAAGTCTG |
| F6 | TC13P3_pool6_12 | GAGATCATGTCCTAACCCTGATCCTCTTGTCCCACAGATATCCAGAACCCTCTAAGAAGTTCCTGCTTTTATTGTTCCCAGAATGTGGTTAGAGACATGACCCTGCCGTGTACCAGCTGAGAGACTCTAAATCCAGTGACAAGTCTG |
| G6 | TC13P3_pool6_13 | GAGATCATGTCCTAACCCTGATCCTCTTGTCCCACAGATATCCAGAACCCTCTAAGAAGTTCCTGCTAATAAAGTTCCGTGAATGTGGTTAGAGACATGACCCTGCCGTGTACCAGCTGAGAGACTCTAAATCCAGTGACAAGTCTG |
| H6 | TC13P3_pool6_14 | GAGATCATGTCCTAACCCTGATCCTCTTGTCCCACAGATATCCAGAACCCTCTAAGAAGTTCCTGCTTATTTAGTTCCTAGAATGTGGTTAGAGACATGACCCTGCCGTGTACCAGCTGAGAGACTCTAAATCCAGTGACAAGTCTG |
| A7 | TC13P3_pool6_15 | GAGATCATGTCCTAACCCTGATCCTCTTGTCCCACAGATATCCAGAACCCTCTAAGAAGTTCCTGCTTATTAAGTTCGATGAATGTGGTTAGAGACATGACCCTGCCGTGTACCAGCTGAGAGACTCTAAATCCAGTGACAAGTCTG |
| B7 | TC13P3_pool6_16 | GAGATCATGTCCTAACCCTGATCCTCTTGTCCCACAGATATCCAGAACCCTCTAAGAAGTTCCTGCTTTTATAGTTCGCTGAATGTGGTTAGAGACATGACCCTGCCGTGTACCAGCTGAGAGACTCTAAATCCAGTGACAAGTCTG |
| C7 | TC13P3_pool6_17 | GAGATCATGTCCTAACCCTGATCCTCTTGTCCCACAGATATCCAGAACCCTCTAAGAAGTTCCTGCTTATTATGTTCGGAGAATGTGGTTAGAGACATGACCCTGCCGTGTACCAGCTGAGAGACTCTAAATCCAGTGACAAGTCTG |
| D7 | TC13P3_pool6_18 | GAGATCATGTCCTAACCCTGATCCTCTTGTCCCACAGATATCCAGAACCCTCTAAGAAGTTCCTGCTATATATGTTCGTTGAATGTGGTTAGAGACATGACCCTGCCGTGTACCAGCTGAGAGACTCTAAATCCAGTGACAAGTCTG |
| E7 | TC13P3_pool6_19 | GAGATCATGTCCTAACCCTGATCCTCTTGTCCCACAGATATCCAGAACCCTCTAAGAAGTTCCTGCTATATTTGTTCTAAGAATGTGGTTAGAGACATGACCCTGCCGTGTACCAGCTGAGAGACTCTAAATCCAGTGACAAGTCTG |
| F7 | TC13P3_pool6_20 | GAGATCATGTCCTAACCCTGATCCTCTTGTCCCACAGATATCCAGAACCCTCTAAGAAGTTCCTGCTATTATAGTTCTCAGAATGTGGTTAGAGACATGACCCTGCCGTGTACCAGCTGAGAGACTCTAAATCCAGTGACAAGTCTG |
| G7 | TC13P3_pool6_21 | GAGATCATGTCCTAACCCTGATCCTCTTGTCCCACAGATATCCAGAACCCTCTAAGAAGTTCCTGCTAATAATGTTCTGAGAATGTGGTTAGAGACATGACCCTGCCGTGTACCAGCTGAGAGACTCTAAATCCAGTGACAAGTCTG |
| H7 | TC13P3_pool6_22 | GAGATCATGTCCTAACCCTGATCCTCTTGTCCCACAGATATCCAGAACCCTCTAAGAAGTTCCTGCTATAATTGTTCTTTGAATGTGGTTAGAGACATGACCCTGCCGTGTACCAGCTGAGAGACTCTAAATCCAGTGACAAGTCTG |
| A8 | TC13P3_pool6_23 | GAGATCATGTCCTAACCCTGATCCTCTTGTCCCACAGATATCCAGAACCCTCTAAGAAGTTCCTGCTAAATATTATCAAAGAATGTGGTTAGAGACATGACCCTGCCGTGTACCAGCTGAGAGACTCTAAATCCAGTGACAAGTCTG |
| B8 | TC13P3_pool6_24 | GAGATCATGTCCTAACCCTGATCCTCTTGTCCCACAGATATCCAGAACCCTCTAAGAAGTTCCTGCTAAAATATATCACAGAATGTGGTTAGAGACATGACCCTGCCGTGTACCAGCTGAGAGACTCTAAATCCAGTGACAAGTCTG |
| C8 | TC13P3_pool6_25 | GAGATCATGTCCTAACCCTGATCCTCTTGTCCCACAGATATCCAGAACCCTCTAAGAAGTTCCTGCTATTTTATATCAGTGAATGTGGTTAGAGACATGACCCTGCCGTGTACCAGCTGAGAGACTCTAAATCCAGTGACAAGTCTG |
| D8 | TC13P3_pool6_26 | GAGATCATGTCCTAACCCTGATCCTCTTGTCCCACAGATATCCAGAACCCTCTAAGAAGTTCCTGCTTTTTATTATCATTGAATGTGGTTAGAGACATGACCCTGCCGTGTACCAGCTGAGAGACTCTAAATCCAGTGACAAGTCTG |
| E8 | TC13P3_pool6_27 | GAGATCATGTCCTAACCCTGATCCTCTTGTCCCACAGATATCCAGAACCCTCTAAGAAGTTCCTGCTTAAAATTATCCAAGAATGTGGTTAGAGACATGACCCTGCCGTGTACCAGCTGAGAGACTCTAAATCCAGTGACAAGTCTG |
| F8 | TC13P3_pool6_28 | GAGATCATGTCCTAACCCTGATCCTCTTGTCCCACAGATATCCAGAACCCTCTAAGAAGTTCCTGCTATTAATTATCCCTGAATGTGGTTAGAGACATGACCCTGCCGTGTACCAGCTGAGAGACTCTAAATCCAGTGACAAGTCTG |
| G8 | TC13P3_pool6_29 | GAGATCATGTCCTAACCCTGATCCTCTTGTCCCACAGATATCCAGAACCCTCTAAGAAGTTCCTGCTTAATAATATCCGAGAATGTGGTTAGAGACATGACCCTGCCGTGTACCAGCTGAGAGACTCTAAATCCAGTGACAAGTCTG |
| H8 | TC13P3_pool6_30 | GAGATCATGTCCTAACCCTGATCCTCTTGTCCCACAGATATCCAGAACCCTCTAAGAAGTTCCTGCTTAAATATATCCTTGAATGTGGTTAGAGACATGACCCTGCCGTGTACCAGCTGAGAGACTCTAAATCCAGTGACAAGTCTG |
| A9 | TC13P3_pool6_31 | GAGATCATGTCCTAACCCTGATCCTCTTGTCCCACAGATATCCAGAACCCTCTAAGAAGTTCCTGCTTATAAATATCGAAGAATGTGGTTAGAGACATGACCCTGCCGTGTACCAGCTGAGAGACTCTAAATCCAGTGACAAGTCTG |
| B9 | TC13P3_pool6_32 | GAGATCATGTCCTAACCCTGATCCTCTTGTCCCACAGATATCCAGAACCCTCTAAGAAGTTCCTGCTTTAAATTATCGCTGAATGTGGTTAGAGACATGACCCTGCCGTGTACCAGCTGAGAGACTCTAAATCCAGTGACAAGTCTG |
| C9 | TC13P3_pool6_33 | GAGATCATGTCCTAACCCTGATCCTCTTGTCCCACAGATATCCAGAACCCTCTAAGAAGTTCCTGCTAATATTTATCGGTGAATGTGGTTAGAGACATGACCCTGCCGTGTACCAGCTGAGAGACTCTAAATCCAGTGACAAGTCTG |
| D9 | TC13P3_pool6_34 | GAGATCATGTCCTAACCCTGATCCTCTTGTCCCACAGATATCCAGAACCCTCTAAGAAGTTCCTGCTTTATAATATCGTTGAATGTGGTTAGAGACATGACCCTGCCGTGTACCAGCTGAGAGACTCTAAATCCAGTGACAAGTCTG |
|  |  |  |
| A1 | TC13P4_pool7_1 | GAGATCATGTCCTAACCCTGATCCTCTTGTCCCACAGATATCCAGAACCCTCTAAGAAGTTCCTGCTTTAATATATCTAAGAATGTGGTTAGAGACATGACCCTGCCGTGTACCAGCTGAGAGACTCTAAATCCAGTGACAAGTCTG |
| B1 | TC13P4_pool7_2 | GAGATCATGTCCTAACCCTGATCCTCTTGTCCCACAGATATCCAGAACCCTCTAAGAAGTTCCTGCTTAATTTTATCTCTGAATGTGGTTAGAGACATGACCCTGCCGTGTACCAGCTGAGAGACTCTAAATCCAGTGACAAGTCTG |
| C1 | TC13P4_pool7_3 | GAGATCATGTCCTAACCCTGATCCTCTTGTCCCACAGATATCCAGAACCCTCTAAGAAGTTCCTGCTAATTTTTATCTGAGAATGTGGTTAGAGACATGACCCTGCCGTGTACCAGCTGAGAGACTCTAAATCCAGTGACAAGTCTG |
| D1 | TC13P4_pool7_4 | GAGATCATGTCCTAACCCTGATCCTCTTGTCCCACAGATATCCAGAACCCTCTAAGAAGTTCCTGCTTTATATTATCTTAGAATGTGGTTAGAGACATGACCCTGCCGTGTACCAGCTGAGAGACTCTAAATCCAGTGACAAGTCTG |
| E1 | TC13P4_pool7_5 | GAGATCATGTCCTAACCCTGATCCTCTTGTCCCACAGATATCCAGAACCCTCTAAGAAGTTCCTGCTAATTATTCTCAATGAATGTGGTTAGAGACATGACCCTGCCGTGTACCAGCTGAGAGACTCTAAATCCAGTGACAAGTCTG |
| F1 | TC13P4_pool7_6 | GAGATCATGTCCTAACCCTGATCCTCTTGTCCCACAGATATCCAGAACCCTCTAAGAAGTTCCTGCTTTTTAATCTCACAGAATGTGGTTAGAGACATGACCCTGCCGTGTACCAGCTGAGAGACTCTAAATCCAGTGACAAGTCTG |
| G1 | TC13P4_pool7_7 | GAGATCATGTCCTAACCCTGATCCTCTTGTCCCACAGATATCCAGAACCCTCTAAGAAGTTCCTGCTATTTATTCTCAGAGAATGTGGTTAGAGACATGACCCTGCCGTGTACCAGCTGAGAGACTCTAAATCCAGTGACAAGTCTG |
| H1 | TC13P4_pool7_8 | GAGATCATGTCCTAACCCTGATCCTCTTGTCCCACAGATATCCAGAACCCTCTAAGAAGTTCCTGCTAAATTATCTCATTGAATGTGGTTAGAGACATGACCCTGCCGTGTACCAGCTGAGAGACTCTAAATCCAGTGACAAGTCTG |
| A2 | TC13P4_pool7_9 | GAGATCATGTCCTAACCCTGATCCTCTTGTCCCACAGATATCCAGAACCCTCTAAGAAGTTCCTGCTATAAAATCTCCAAGAATGTGGTTAGAGACATGACCCTGCCGTGTACCAGCTGAGAGACTCTAAATCCAGTGACAAGTCTG |
| B2 | TC13P4_pool7_10 | GAGATCATGTCCTAACCCTGATCCTCTTGTCCCACAGATATCCAGAACCCTCTAAGAAGTTCCTGCTTATAATTCTCCCTGAATGTGGTTAGAGACATGACCCTGCCGTGTACCAGCTGAGAGACTCTAAATCCAGTGACAAGTCTG |
| C2 | TC13P4_pool7_11 | GAGATCATGTCCTAACCCTGATCCTCTTGTCCCACAGATATCCAGAACCCTCTAAGAAGTTCCTGCTAAAAATTCTCCGTGAATGTGGTTAGAGACATGACCCTGCCGTGTACCAGCTGAGAGACTCTAAATCCAGTGACAAGTCTG |
| D2 | TC13P4_pool7_12 | GAGATCATGTCCTAACCCTGATCCTCTTGTCCCACAGATATCCAGAACCCTCTAAGAAGTTCCTGCTTTTATTTCTCCTAGAATGTGGTTAGAGACATGACCCTGCCGTGTACCAGCTGAGAGACTCTAAATCCAGTGACAAGTCTG |
| E2 | TC13P4_pool7_13 | GAGATCATGTCCTAACCCTGATCCTCTTGTCCCACAGATATCCAGAACCCTCTAAGAAGTTCCTGCTAATAAATCTCGATGAATGTGGTTAGAGACATGACCCTGCCGTGTACCAGCTGAGAGACTCTAAATCCAGTGACAAGTCTG |
| F2 | TC13P4_pool7_14 | GAGATCATGTCCTAACCCTGATCCTCTTGTCCCACAGATATCCAGAACCCTCTAAGAAGTTCCTGCTTATTTATCTCGCAGAATGTGGTTAGAGACATGACCCTGCCGTGTACCAGCTGAGAGACTCTAAATCCAGTGACAAGTCTG |
| G2 | TC13P4_pool7_15 | GAGATCATGTCCTAACCCTGATCCTCTTGTCCCACAGATATCCAGAACCCTCTAAGAAGTTCCTGCTTATTAATCTCGGTGAATGTGGTTAGAGACATGACCCTGCCGTGTACCAGCTGAGAGACTCTAAATCCAGTGACAAGTCTG |
| H2 | TC13P4_pool7_16 | GAGATCATGTCCTAACCCTGATCCTCTTGTCCCACAGATATCCAGAACCCTCTAAGAAGTTCCTGCTTTTATATCTCGTTGAATGTGGTTAGAGACATGACCCTGCCGTGTACCAGCTGAGAGACTCTAAATCCAGTGACAAGTCTG |
| A3 | TC13P4_pool7_17 | GAGATCATGTCCTAACCCTGATCCTCTTGTCCCACAGATATCCAGAACCCTCTAAGAAGTTCCTGCTTATTATTCTCTAAGAATGTGGTTAGAGACATGACCCTGCCGTGTACCAGCTGAGAGACTCTAAATCCAGTGACAAGTCTG |
| B3 | TC13P4_pool7_18 | GAGATCATGTCCTAACCCTGATCCTCTTGTCCCACAGATATCCAGAACCCTCTAAGAAGTTCCTGCTATATATTCTCTCTGAATGTGGTTAGAGACATGACCCTGCCGTGTACCAGCTGAGAGACTCTAAATCCAGTGACAAGTCTG |
| C3 | TC13P4_pool7_19 | GAGATCATGTCCTAACCCTGATCCTCTTGTCCCACAGATATCCAGAACCCTCTAAGAAGTTCCTGCTATATTTTCTCTGAGAATGTGGTTAGAGACATGACCCTGCCGTGTACCAGCTGAGAGACTCTAAATCCAGTGACAAGTCTG |
| D3 | TC13P4_pool7_20 | GAGATCATGTCCTAACCCTGATCCTCTTGTCCCACAGATATCCAGAACCCTCTAAGAAGTTCCTGCTATTATATCTCTTAGAATGTGGTTAGAGACATGACCCTGCCGTGTACCAGCTGAGAGACTCTAAATCCAGTGACAAGTCTG |
| E3 | TC13P4_pool7_21 | GAGATCATGTCCTAACCCTGATCCTCTTGTCCCACAGATATCCAGAACCCTCTAAGAAGTTCCTGCTAATAATTGTCAAAGAATGTGGTTAGAGACATGACCCTGCCGTGTACCAGCTGAGAGACTCTAAATCCAGTGACAAGTCTG |
| F3 | TC13P4_pool7_22 | GAGATCATGTCCTAACCCTGATCCTCTTGTCCCACAGATATCCAGAACCCTCTAAGAAGTTCCTGCTATAATTTGTCACTGAATGTGGTTAGAGACATGACCCTGCCGTGTACCAGCTGAGAGACTCTAAATCCAGTGACAAGTCTG |
| G3 | TC13P4_pool7_23 | GAGATCATGTCCTAACCCTGATCCTCTTGTCCCACAGATATCCAGAACCCTCTAAGAAGTTCCTGCTAAATATTGTCAGAGAATGTGGTTAGAGACATGACCCTGCCGTGTACCAGCTGAGAGACTCTAAATCCAGTGACAAGTCTG |
| H3 | TC13P4_pool7_24 | GAGATCATGTCCTAACCCTGATCCTCTTGTCCCACAGATATCCAGAACCCTCTAAGAAGTTCCTGCTAAAATATGTCATAGAATGTGGTTAGAGACATGACCCTGCCGTGTACCAGCTGAGAGACTCTAAATCCAGTGACAAGTCTG |
| A4 | TC13P4_pool7_25 | GAGATCATGTCCTAACCCTGATCCTCTTGTCCCACAGATATCCAGAACCCTCTAAGAAGTTCCTGCTATTTTATGTCCATGAATGTGGTTAGAGACATGACCCTGCCGTGTACCAGCTGAGAGACTCTAAATCCAGTGACAAGTCTG |
| B4 | TC13P4_pool7_26 | GAGATCATGTCCTAACCCTGATCCTCTTGTCCCACAGATATCCAGAACCCTCTAAGAAGTTCCTGCTTTTTATTGTCCCTGAATGTGGTTAGAGACATGACCCTGCCGTGTACCAGCTGAGAGACTCTAAATCCAGTGACAAGTCTG |
| C4 | TC13P4_pool7_27 | GAGATCATGTCCTAACCCTGATCCTCTTGTCCCACAGATATCCAGAACCCTCTAAGAAGTTCCTGCTTAAAATTGTCCGAGAATGTGGTTAGAGACATGACCCTGCCGTGTACCAGCTGAGAGACTCTAAATCCAGTGACAAGTCTG |
| D4 | TC13P4_pool7_28 | GAGATCATGTCCTAACCCTGATCCTCTTGTCCCACAGATATCCAGAACCCTCTAAGAAGTTCCTGCTATTAATTGTCCTTGAATGTGGTTAGAGACATGACCCTGCCGTGTACCAGCTGAGAGACTCTAAATCCAGTGACAAGTCTG |
| E4 | TC13P4_pool7_29 | GAGATCATGTCCTAACCCTGATCCTCTTGTCCCACAGATATCCAGAACCCTCTAAGAAGTTCCTGCTTAATAATGTCGAAGAATGTGGTTAGAGACATGACCCTGCCGTGTACCAGCTGAGAGACTCTAAATCCAGTGACAAGTCTG |
| F4 | TC13P4_pool7_30 | GAGATCATGTCCTAACCCTGATCCTCTTGTCCCACAGATATCCAGAACCCTCTAAGAAGTTCCTGCTTAAATATGTCGCTGAATGTGGTTAGAGACATGACCCTGCCGTGTACCAGCTGAGAGACTCTAAATCCAGTGACAAGTCTG |
| G4 | TC13P4_pool7_31 | GAGATCATGTCCTAACCCTGATCCTCTTGTCCCACAGATATCCAGAACCCTCTAAGAAGTTCCTGCTTATAAATGTCGGAGAATGTGGTTAGAGACATGACCCTGCCGTGTACCAGCTGAGAGACTCTAAATCCAGTGACAAGTCTG |
| H4 | TC13P4_pool7_32 | GAGATCATGTCCTAACCCTGATCCTCTTGTCCCACAGATATCCAGAACCCTCTAAGAAGTTCCTGCTTTAAATTGTCGTTGAATGTGGTTAGAGACATGACCCTGCCGTGTACCAGCTGAGAGACTCTAAATCCAGTGACAAGTCTG |
| A5 | TC13P4_pool7_33 | GAGATCATGTCCTAACCCTGATCCTCTTGTCCCACAGATATCCAGAACCCTCTAAGAAGTTCCTGCTAATATTTGTCTATGAATGTGGTTAGAGACATGACCCTGCCGTGTACCAGCTGAGAGACTCTAAATCCAGTGACAAGTCTG |
| B5 | TC13P4_pool7_34 | GAGATCATGTCCTAACCCTGATCCTCTTGTCCCACAGATATCCAGAACCCTCTAAGAAGTTCCTGCTTTATAATGTCTCTGAATGTGGTTAGAGACATGACCCTGCCGTGTACCAGCTGAGAGACTCTAAATCCAGTGACAAGTCTG |
|  |  |  |
| C5 | TC13P4_pool8_1 | GAGATCATGTCCTAACCCTGATCCTCTTGTCCCACAGATATCCAGAACCCTCTAAGAAGTTCCTGCTTTAATATGTCTGAGAATGTGGTTAGAGACATGACCCTGCCGTGTACCAGCTGAGAGACTCTAAATCCAGTGACAAGTCTG |
| D5 | TC13P4_pool8_2 | GAGATCATGTCCTAACCCTGATCCTCTTGTCCCACAGATATCCAGAACCCTCTAAGAAGTTCCTGCTTAATTTTGTCTTTGAATGTGGTTAGAGACATGACCCTGCCGTGTACCAGCTGAGAGACTCTAAATCCAGTGACAAGTCTG |
| E5 | TC13P4_pool8_3 | GAGATCATGTCCTAACCCTGATCCTCTTGTCCCACAGATATCCAGAACCCTCTAAGAAGTTCCTGCTAATTTTTTTCAAAGAATGTGGTTAGAGACATGACCCTGCCGTGTACCAGCTGAGAGACTCTAAATCCAGTGACAAGTCTG |
| F5 | TC13P4_pool8_4 | GAGATCATGTCCTAACCCTGATCCTCTTGTCCCACAGATATCCAGAACCCTCTAAGAAGTTCCTGCTTTATATTTTCACAGAATGTGGTTAGAGACATGACCCTGCCGTGTACCAGCTGAGAGACTCTAAATCCAGTGACAAGTCTG |
| G5 | TC13P4_pool8_5 | GAGATCATGTCCTAACCCTGATCCTCTTGTCCCACAGATATCCAGAACCCTCTAAGAAGTTCCTGCTAATTATTTTCAGTGAATGTGGTTAGAGACATGACCCTGCCGTGTACCAGCTGAGAGACTCTAAATCCAGTGACAAGTCTG |
| H5 | TC13P4_pool8_6 | GAGATCATGTCCTAACCCTGATCCTCTTGTCCCACAGATATCCAGAACCCTCTAAGAAGTTCCTGCTTTTTAATTTCATAGAATGTGGTTAGAGACATGACCCTGCCGTGTACCAGCTGAGAGACTCTAAATCCAGTGACAAGTCTG |
| A6 | TC13P4_pool8_7 | GAGATCATGTCCTAACCCTGATCCTCTTGTCCCACAGATATCCAGAACCCTCTAAGAAGTTCCTGCTATTTATTTTCCAAGAATGTGGTTAGAGACATGACCCTGCCGTGTACCAGCTGAGAGACTCTAAATCCAGTGACAAGTCTG |
| B6 | TC13P4_pool8_8 | GAGATCATGTCCTAACCCTGATCCTCTTGTCCCACAGATATCCAGAACCCTCTAAGAAGTTCCTGCTAAATTATTTCCCTGAATGTGGTTAGAGACATGACCCTGCCGTGTACCAGCTGAGAGACTCTAAATCCAGTGACAAGTCTG |
| C6 | TC13P4_pool8_9 | GAGATCATGTCCTAACCCTGATCCTCTTGTCCCACAGATATCCAGAACCCTCTAAGAAGTTCCTGCTATAAAATTTCCGAGAATGTGGTTAGAGACATGACCCTGCCGTGTACCAGCTGAGAGACTCTAAATCCAGTGACAAGTCTG |
| D6 | TC13P4_pool8_10 | GAGATCATGTCCTAACCCTGATCCTCTTGTCCCACAGATATCCAGAACCCTCTAAGAAGTTCCTGCTTATAATTTTCCTTGAATGTGGTTAGAGACATGACCCTGCCGTGTACCAGCTGAGAGACTCTAAATCCAGTGACAAGTCTG |
| E6 | TC13P4_pool8_11 | GAGATCATGTCCTAACCCTGATCCTCTTGTCCCACAGATATCCAGAACCCTCTAAGAAGTTCCTGCTAAAAATTTTCGATGAATGTGGTTAGAGACATGACCCTGCCGTGTACCAGCTGAGAGACTCTAAATCCAGTGACAAGTCTG |
| F6 | TC13P4_pool8_12 | GAGATCATGTCCTAACCCTGATCCTCTTGTCCCACAGATATCCAGAACCCTCTAAGAAGTTCCTGCTTTTATTTTTCGCAGAATGTGGTTAGAGACATGACCCTGCCGTGTACCAGCTGAGAGACTCTAAATCCAGTGACAAGTCTG |
| G6 | TC13P4_pool8_13 | GAGATCATGTCCTAACCCTGATCCTCTTGTCCCACAGATATCCAGAACCCTCTAAGAAGTTCCTGCTAATAAATTTCGGTGAATGTGGTTAGAGACATGACCCTGCCGTGTACCAGCTGAGAGACTCTAAATCCAGTGACAAGTCTG |
| H6 | TC13P4_pool8_14 | GAGATCATGTCCTAACCCTGATCCTCTTGTCCCACAGATATCCAGAACCCTCTAAGAAGTTCCTGCTTATTTATTTCGTAGAATGTGGTTAGAGACATGACCCTGCCGTGTACCAGCTGAGAGACTCTAAATCCAGTGACAAGTCTG |
| A7 | TC13P4_pool8_15 | GAGATCATGTCCTAACCCTGATCCTCTTGTCCCACAGATATCCAGAACCCTCTAAGAAGTTCCTGCTTATTAATTTCTATGAATGTGGTTAGAGACATGACCCTGCCGTGTACCAGCTGAGAGACTCTAAATCCAGTGACAAGTCTG |
| B7 | TC13P4_pool8_16 | GAGATCATGTCCTAACCCTGATCCTCTTGTCCCACAGATATCCAGAACCCTCTAAGAAGTTCCTGCTTTTATATTTCTCTGAATGTGGTTAGAGACATGACCCTGCCGTGTACCAGCTGAGAGACTCTAAATCCAGTGACAAGTCTG |
| C7 | TC13P4_pool8_17 | GAGATCATGTCCTAACCCTGATCCTCTTGTCCCACAGATATCCAGAACCCTCTAAGAAGTTCCTGCTTATTATTTTCTGAGAATGTGGTTAGAGACATGACCCTGCCGTGTACCAGCTGAGAGACTCTAAATCCAGTGACAAGTCTG |
| D7 | TC13P4_pool8_18 | GAGATCATGTCCTAACCCTGATCCTCTTGTCCCACAGATATCCAGAACCCTCTAAGAAGTTCCTGCTATATATTTTCTTTGAATGTGGTTAGAGACATGACCCTGCCGTGTACCAGCTGAGAGACTCTAAATCCAGTGACAAGTCTG |

**Supplementary Table 4. Nuclear and protein sequences of the different TALEB architectures. pCLS36448, pCLS37610, pCLS37657 (from left to right: black= TAL array; pink= 3aa linker; red= G1397 DddAtox-N-terminal; orange= 4aa liker; purple= 1X UGI). pCLS36495, pCLS37633, pCLS37680 (from left to right: black= TAL array; pink= 3aa linker; blue= G1397 DddAtox-C-terminal; orange= 4aa liker; purple= 1X UGI)**

| Plasmid | Nucl. Seq. | Prot. Seq. |
| --- | --- | --- |
| pCLS36448 | ATGGGCGATCCTAAAAAGAAACGTAAGGTCATCGATATCGCCGATCTACGCACGCTCGGCTACAGCCAGCAGCAACAGGAGAAGATCAAACCGAAGGTTCGTTCGACAGTGGCGCAGCACCACGAGGCACTGGTCGGCCACGGGTTTACACACGCGCACATCGTTGCGTTAAGCCAACACCCGGCAGCGTTAGGGACCGTCGCTGTCAAGTATCAGGACATGATCGCAGCGTTGCCAGAGGCGACACACGAAGCGATCGTTGGCGTCGGCAAACAGTGGTCCGGCGCACGCGCTCTGGAGGCCTTGCTCACGGTGGCGGGAGAGTTGAGAGGTCCACCGTTACAGTTGGACACAGGCCAACTTCTCAAGATTGCAAAACGTGGCGGCGTGACCGCAGTGGAGGCAGTGCATGCATGGCGCAATGCACTGACGGGTGCCCCGCTCAACTTGACCCCGGAGCAGGTGGTGGCCATCGCCAGCCACGATGGCGGCAAGCAGGCGCTGGAGACGGTCCAGCGGCTGTTGCCGGTGCTGTGCCAGGCCCACGGCTTGACCCCCCAGCAGGTGGTGGCCATCGCCAGCAATGGCGGTGGCAAGCAGGCGCTGGAGACGGTCCAGCGGCTGTTGCCGGTGCTGTGCCAGGCCCACGGCTTGACCCCGGAGCAGGTGGTGGCCATCGCCAGCAATATTGGTGGCAAGCAGGCGCTGGAGACGGTGCAGGCGCTGTTGCCGGTGCTGTGCCAGGCCCACGGCTTGACCCCGGAGCAGGTGGTGGCCATCGCCAGCAATATTGGTGGCAAGCAGGCGCTGGAGACGGTGCAGGCGCTGTTGCCGGTGCTGTGCCAGGCCCACGGCTTGACCCCCCAGCAGGTGGTGGCCATCGCCAGCAATAATGGTGGCAAGCAGGCGCTGGAGACGGTCCAGCGGCTGTTGCCGGTGCTGTGCCAGGCCCACGGCTTGACCCCGGAGCAGGTGGTGGCCATCGCCAGCAATATTGGTGGCAAGCAGGCGCTGGAGACGGTGCAGGCGCTGTTGCCGGTGCTGTGCCAGGCCCACGGCTTGACCCCGGAGCAGGTGGTGGCCATCGCCAGCAATATTGGTGGCAAGCAGGCGCTGGAGACGGTGCAGGCGCTGTTGCCGGTGCTGTGCCAGGCCCACGGCTTGACCCCCCAGCAGGTGGTGGCCATCGCCAGCAATAATGGTGGCAAGCAGGCGCTGGAGACGGTCCAGCGGCTGTTGCCGGTGCTGTGCCAGGCCCACGGCTTGACCCCCCAGCAGGTGGTGGCCATCGCCAGCAATGGCGGTGGCAAGCAGGCGCTGGAGACGGTCCAGCGGCTGTTGCCGGTGCTGTGCCAGGCCCACGGCTTGACCCCCCAGCAGGTGGTGGCCATCGCCAGCAATGGCGGTGGCAAGCAGGCGCTGGAGACGGTCCAGCGGCTGTTGCCGGTGCTGTGCCAGGCCCACGGCTTGACCCCGGAGCAGGTGGTGGCCATCGCCAGCCACGATGGCGGCAAGCAGGCGCTGGAGACGGTCCAGCGGCTGTTGCCGGTGCTGTGCCAGGCCCACGGCTTGACCCCGGAGCAGGTGGTGGCCATCGCCAGCCACGATGGCGGCAAGCAGGCGCTGGAGACGGTCCAGCGGCTGTTGCCGGTGCTGTGCCAGGCCCACGGCTTGACCCCCCAGCAGGTGGTGGCCATCGCCAGCAATGGCGGTGGCAAGCAGGCGCTGGAGACGGTCCAGCGGCTGTTGCCGGTGCTGTGCCAGGCCCACGGCTTGACCCCCCAGCAGGTGGTGGCCATCGCCAGCAATAATGGTGGCAAGCAGGCGCTGGAGACGGTCCAGCGGCTGTTGCCGGTGCTGTGCCAGGCCCACGGCTTGACCCCGGAGCAGGTGGTGGCCATCGCCAGCCACGATGGCGGCAAGCAGGCGCTGGAGACGGTCCAGCGGCTGTTGCCGGTGCTGTGCCAGGCCCACGGCTTGACCCCTCAGCAGGTGGTGGCCATCGCCAGCAATGGCGGCGGCAGGCCGGCGCTGGAGAGCATTGTTGCCCAGTTATCTCGCCCTGATCCGGCGTTGGCCGCGTTGACCAACGACCACCTCGTCGCCTTGGCCTGCCTCGGCGGGCGTCCTGCGCTGGATGCAGTGAAAAAGGGATTGGGGGGCTCCGGTAGCTACGCGCTGGGACCCTATCAAATTTCGGCCCCCCAGCTGCCCGCCTACAACGGACAGACGGTGGGCACGTTCTACTACGTGAACGACGCAGGTGGCCTGGAGTCCAAGGTGTTTTCTTCTGGTGGCCCTACCCCCTATCCCAACTACGCCAACGCGGGCCACGTGGAGGGCCAGTCCGCCCTGTTCATGCGCGACAACGGCATCTCTGAGGGCCTGGTGTTCCACAACAACCCAGAGGGCACCTGCGGGTTCTGCGTGAATATGACCGAGACTCTGCTTCCTGAAAATGCTAAGATGACAGTCGTTCCGCCTGAAGGGTCCGGAGGCTCGACCAACCTGTCCGACATCATCGAGAAGGAGACTGGCAAACAGTTGGTGATCCAGGAGAGCATCCTGATGCTGCCGGAGGAGGTGGAGGAGGTAATCGGCAACAAGCCCGAGAGTGACATATTGGTCCATACTGCGTACGACGAATCCACCGACGAGAACGTGATGCTGCTGACCAGCGATGCTCCAGAGTACAAGCCGTGGGCCCTCGTCATCCAGGACTCAAATGGGGAGAACAAGATCAAAATGCTATGATAG | MGDPKKKRKVIDIADLRTLGYSQQQQEKIKPKVRSTVAQHHEALVGHGFTHAHIVALSQHPAALGTVAVKYQDMIAALPEATHEAIVGVGKQWSGARALEALLTVAGELRGPPLQLDTGQLLKIAKRGGVTAVEAVHAWRNALTGAPLNLTPEQVVAIASHDGGKQALETVQRLLPVLCQAHGLTPQQVVAIASNGGGKQALETVQRLLPVLCQAHGLTPEQVVAIASNIGGKQALETVQALLPVLCQAHGLTPEQVVAIASNIGGKQALETVQALLPVLCQAHGLTPQQVVAIASNNGGKQALETVQRLLPVLCQAHGLTPEQVVAIASNIGGKQALETVQALLPVLCQAHGLTPEQVVAIASNIGGKQALETVQALLPVLCQAHGLTPQQVVAIASNNGGKQALETVQRLLPVLCQAHGLTPQQVVAIASNGGGKQALETVQRLLPVLCQAHGLTPQQVVAIASNGGGKQALETVQRLLPVLCQAHGLTPEQVVAIASHDGGKQALETVQRLLPVLCQAHGLTPEQVVAIASHDGGKQALETVQRLLPVLCQAHGLTPQQVVAIASNGGGKQALETVQRLLPVLCQAHGLTPQQVVAIASNNGGKQALETVQRLLPVLCQAHGLTPEQVVAIASHDGGKQALETVQRLLPVLCQAHGLTPQQVVAIASNGGGRPALESIVAQLSRPDPALAALTNDHLVALACLGGRPALDAVKKGLGGSGSYALGPYQISAPQLPAYNGQTVGTFYYVNDAGGLESKVFSSGGPTPYPNYANAGHVEGQSALFMRDNGISEGLVFHNNPEGTCGFCVNMTETLLPENAKMTVVPPEGSGGSTNLSDIIEKETGKQLVIQESILMLPEEVEEVIGNKPESDILVHTAYDESTDENVMLLTSDAPEYKPWALVIQDSNGENKIKML |
| pCLS37610 | ATGGGCGATCCTAAAAAGAAACGTAAGGTCATCGATATCGCCGATCTACGCACGCTCGGCTACAGCCAGCAGCAACAGGAGAAGATCAAACCGAAGGTTCGTTCGACAGTGGCGCAGCACCACGAGGCACTGGTCGGCCACGGGTTTACACACGCGCACATCGTTGCGTTAAGCCAACACCCGGCAGCGTTAGGGACCGTCGCTGTCAAGTATCAGGACATGATCGCAGCGTTGCCAGAGGCGACACACGAAGCGATCGTTGGCGTCGGCAAACAGTGGTCCGGCGCACGCGCTCTGGAGGCCTTGCTCACGGTGGCGGGAGAGTTGAGAGGTCCACCGTTACAGTTGGACACAGGCCAACTTCTCAAGATTGCAAAACGTGGCGGCGTGACCGCAGTGGAGGCAGTGCATGCATGGCGCAATGCACTGACGGGTGCCCCGCTCAACTTGACCCCGGAGCAGGTGGTGGCCATCGCCAGCCACGATGGCGGCAAGCAGGCGCTGGAGACGGTCCAGCGGCTGTTGCCGGTGCTGTGCCAGGCCCACGGCTTGACCCCCCAGCAGGTGGTGGCCATCGCCAGCAATGGCGGTGGCAAGCAGGCGCTGGAGACGGTCCAGCGGCTGTTGCCGGTGCTGTGCCAGGCCCACGGCTTGACCCCGGAGCAGGTGGTGGCCATCGCCAGCAATATTGGTGGCAAGCAGGCGCTGGAGACGGTGCAGGCGCTGTTGCCGGTGCTGTGCCAGGCCCACGGCTTGACCCCGGAGCAGGTGGTGGCCATCGCCAGCAATATTGGTGGCAAGCAGGCGCTGGAGACGGTGCAGGCGCTGTTGCCGGTGCTGTGCCAGGCCCACGGCTTGACCCCCCAGCAGGTGGTGGCCATCGCCAGCAATAATGGTGGCAAGCAGGCGCTGGAGACGGTCCAGCGGCTGTTGCCGGTGCTGTGCCAGGCCCACGGCTTGACCCCGGAGCAGGTGGTGGCCATCGCCAGCAATATTGGTGGCAAGCAGGCGCTGGAGACGGTGCAGGCGCTGTTGCCGGTGCTGTGCCAGGCCCACGGCTTGACCCCGGAGCAGGTGGTGGCCATCGCCAGCAATATTGGTGGCAAGCAGGCGCTGGAGACGGTGCAGGCGCTGTTGCCGGTGCTGTGCCAGGCCCACGGCTTGACCCCCCAGCAGGTGGTGGCCATCGCCAGCAATAATGGTGGCAAGCAGGCGCTGGAGACGGTCCAGCGGCTGTTGCCGGTGCTGTGCCAGGCCCACGGCTTGACCCCCCAGCAGGTGGTGGCCATCGCCAGCAATGGCGGTGGCAAGCAGGCGCTGGAGACGGTCCAGCGGCTGTTGCCGGTGCTGTGCCAGGCCCACGGCTTGACCCCCCAGCAGGTGGTGGCCATCGCCAGCAATGGCGGTGGCAAGCAGGCGCTGGAGACGGTCCAGCGGCTGTTGCCGGTGCTGTGCCAGGCCCACGGCTTGACCCCGGAGCAGGTGGTGGCCATCGCCAGCCACGATGGCGGCAAGCAGGCGCTGGAGACGGTCCAGCGGCTGTTGCCGGTGCTGTGCCAGGCCCACGGCTTGACCCCGGAGCAGGTGGTGGCCATCGCCAGCCACGATGGCGGCAAGCAGGCGCTGGAGACGGTCCAGCGGCTGTTGCCGGTGCTGTGCCAGGCCCACGGCTTGACCCCCCAGCAGGTGGTGGCCATCGCCAGCAATGGCGGTGGCAAGCAGGCGCTGGAGACGGTCCAGCGGCTGTTGCCGGTGCTGTGCCAGGCCCACGGCTTGACCCCCCAGCAGGTGGTGGCCATCGCCAGCAATAATGGTGGCAAGCAGGCGCTGGAGACGGTCCAGCGGCTGTTGCCGGTGCTGTGCCAGGCCCACGGCTTGACCCCGGAGCAGGTGGTGGCCATCGCCAGCCACGATGGCGGCAAGCAGGCGCTGGAGACGGTCCAGCGGCTGTTGCCGGTGCTGTGCCAGGCCCACGGCTTGACCCCTCAGCAGGTGGTGGCCATCGCCAGCAATGGCGGCGGCAGGCCGGCGCTGGAGGGGGGCTCCGGTAGCTACGCGCTGGGACCCTATCAAATTTCGGCCCCCCAGCTGCCCGCCTACAACGGACAGACGGTGGGCACGTTCTACTACGTGAACGACGCAGGTGGCCTGGAGTCCAAGGTGTTTTCTTCTGGTGGCCCTACCCCCTATCCCAACTACGCCAACGCGGGCCACGTGGAGGGCCAGTCCGCCCTGTTCATGCGCGACAACGGCATCTCTGAGGGCCTGGTGTTCCACAACAACCCAGAGGGCACCTGCGGGTTCTGCGTGAATATGACCGAGACTCTGCTTCCTGAAAATGCTAAGATGACAGTCGTTCCGCCTGAAGGGTCCGGAGGCTCGACCAACCTGTCCGACATCATCGAGAAGGAGACTGGCAAACAGTTGGTGATCCAGGAGAGCATCCTGATGCTGCCGGAGGAGGTGGAGGAGGTAATCGGCAACAAGCCCGAGAGTGACATATTGGTCCATACTGCGTACGACGAATCCACCGACGAGAACGTGATGCTGCTGACCAGCGATGCTCCAGAGTACAAGCCGTGGGCCCTCGTCATCCAGGACTCAAATGGGGAGAACAAGATCAAAATGCTATGATAG | MGDPKKKRKVIDIADLRTLGYSQQQQEKIKPKVRSTVAQHHEALVGHGFTHAHIVALSQHPAALGTVAVKYQDMIAALPEATHEAIVGVGKQWSGARALEALLTVAGELRGPPLQLDTGQLLKIAKRGGVTAVEAVHAWRNALTGAPLNLTPEQVVAIASHDGGKQALETVQRLLPVLCQAHGLTPQQVVAIASNGGGKQALETVQRLLPVLCQAHGLTPEQVVAIASNIGGKQALETVQALLPVLCQAHGLTPEQVVAIASNIGGKQALETVQALLPVLCQAHGLTPQQVVAIASNNGGKQALETVQRLLPVLCQAHGLTPEQVVAIASNIGGKQALETVQALLPVLCQAHGLTPEQVVAIASNIGGKQALETVQALLPVLCQAHGLTPQQVVAIASNNGGKQALETVQRLLPVLCQAHGLTPQQVVAIASNGGGKQALETVQRLLPVLCQAHGLTPQQVVAIASNGGGKQALETVQRLLPVLCQAHGLTPEQVVAIASHDGGKQALETVQRLLPVLCQAHGLTPEQVVAIASHDGGKQALETVQRLLPVLCQAHGLTPQQVVAIASNGGGKQALETVQRLLPVLCQAHGLTPQQVVAIASNNGGKQALETVQRLLPVLCQAHGLTPEQVVAIASHDGGKQALETVQRLLPVLCQAHGLTPQQVVAIASNGGGRPALEGGSGSYALGPYQISAPQLPAYNGQTVGTFYYVNDAGGLESKVFSSGGPTPYPNYANAGHVEGQSALFMRDNGISEGLVFHNNPEGTCGFCVNMTETLLPENAKMTVVPPEGSGGSTNLSDIIEKETGKQLVIQESILMLPEEVEEVIGNKPESDILVHTAYDESTDENVMLLTSDAPEYKPWALVIQDSNGENKIKML |
| pCLS37657 | ATGGGCGATCCTAAAAAGAAACGTAAGGTCATCGATATCGCCGATCTACGCACGCTCGGCTACAGCCAGCAGCAACAGGAGAAGATCAAACCGAAGGTTCGTTCGACAGTGGCGCAGCACCACGAGGCACTGGTCGGCCACGGGTTTACACACGCGCACATCGTTGCGTTAAGCCAACACCCGGCAGCGTTAGGGACCGTCGCTGTCAAGTATCAGGACATGATCGCAGCGTTGCCAGAGGCGACACACGAAGCGATCGTTGGCGTCGGCAAACAGTGGTCCGGCGCACGCGCTCTGGAGGCCTTGCTCACGGTGGCGGGAGAGTTGAGAGGTCCACCGTTACAGTTGGACACAGGCCAACTTCTCAAGATTGCAAAACGTGGCGGCGTGACCGCAGTGGAGGCAGTGCATGCATGGCGCAATGCACTGACGGGTGCCCCGCTCAACTTGACCCCGGAGCAGGTGGTGGCCATCGCCAGCCACGATGGCGGCAAGCAGGCGCTGGAGACGGTCCAGCGGCTGTTGCCGGTGCTGTGCCAGGCCCACGGCTTGACCCCCCAGCAGGTGGTGGCCATCGCCAGCAATGGCGGTGGCAAGCAGGCGCTGGAGACGGTCCAGCGGCTGTTGCCGGTGCTGTGCCAGGCCCACGGCTTGACCCCGGAGCAGGTGGTGGCCATCGCCAGCAATATTGGTGGCAAGCAGGCGCTGGAGACGGTGCAGGCGCTGTTGCCGGTGCTGTGCCAGGCCCACGGCTTGACCCCGGAGCAGGTGGTGGCCATCGCCAGCAATATTGGTGGCAAGCAGGCGCTGGAGACGGTGCAGGCGCTGTTGCCGGTGCTGTGCCAGGCCCACGGCTTGACCCCCCAGCAGGTGGTGGCCATCGCCAGCAATAATGGTGGCAAGCAGGCGCTGGAGACGGTCCAGCGGCTGTTGCCGGTGCTGTGCCAGGCCCACGGCTTGACCCCGGAGCAGGTGGTGGCCATCGCCAGCAATATTGGTGGCAAGCAGGCGCTGGAGACGGTGCAGGCGCTGTTGCCGGTGCTGTGCCAGGCCCACGGCTTGACCCCGGAGCAGGTGGTGGCCATCGCCAGCAATATTGGTGGCAAGCAGGCGCTGGAGACGGTGCAGGCGCTGTTGCCGGTGCTGTGCCAGGCCCACGGCTTGACCCCCCAGCAGGTGGTGGCCATCGCCAGCAATAATGGTGGCAAGCAGGCGCTGGAGACGGTCCAGCGGCTGTTGCCGGTGCTGTGCCAGGCCCACGGCTTGACCCCCCAGCAGGTGGTGGCCATCGCCAGCAATGGCGGTGGCAAGCAGGCGCTGGAGACGGTCCAGCGGCTGTTGCCGGTGCTGTGCCAGGCCCACGGCTTGACCCCCCAGCAGGTGGTGGCCATCGCCAGCAATGGCGGTGGCAAGCAGGCGCTGGAGACGGTCCAGCGGCTGTTGCCGGTGCTGTGCCAGGCCCACGGCTTGACCCCGGAGCAGGTGGTGGCCATCGCCAGCCACGATGGCGGCAAGCAGGCGCTGGAGACGGTCCAGCGGCTGTTGCCGGTGCTGTGCCAGGCCCACGGCTTGACCCCGGAGCAGGTGGTGGCCATCGCCAGCCACGATGGCGGCAAGCAGGCGCTGGAGACGGTCCAGCGGCTGTTGCCGGTGCTGTGCCAGGCCCACGGCTTGACCCCCCAGCAGGTGGTGGCCATCGCCAGCAATGGCGGTGGCAAGCAGGCGCTGGAGACGGTCCAGCGGCTGTTGCCGGTGCTGTGCCAGGCCCACGGCTTGACCCCCCAGCAGGTGGTGGCCATCGCCAGCAATAATGGTGGCAAGCAGGCGCTGGAGACGGTCCAGCGGCTGTTGCCGGTGCTGTGCCAGGCCCACGGCTTGACCCCGGAGCAGGTGGTGGCCATCGCCAGCCACGATGGCGGCAAGCAGGCGCTGGAGACGGTCCAGCGGCTGTTGCCGGTGCTGTGCCAGGCCCACGGCTTGACCCCTCAGCAGGTGGTGGCCATCGCCAGCAATGGCGGCGGCAGGCCGGCGCTGGAGAGCATTGTTGCCCAGTTATCTCGCCCTGATCCGAGTGGCAGCGGAAGTGGCGGGGGCTCCGGTAGCTACGCGCTGGGACCCTATCAAATTTCGGCCCCCCAGCTGCCCGCCTACAACGGACAGACGGTGGGCACGTTCTACTACGTGAACGACGCAGGTGGCCTGGAGTCCAAGGTGTTTTCTTCTGGTGGCCCTACCCCCTATCCCAACTACGCCAACGCGGGCCACGTGGAGGGCCAGTCCGCCCTGTTCATGCGCGACAACGGCATCTCTGAGGGCCTGGTGTTCCACAACAACCCAGAGGGCACCTGCGGGTTCTGCGTGAATATGACCGAGACTCTGCTTCCTGAAAATGCTAAGATGACAGTCGTTCCGCCTGAAGGGTCCGGAGGCTCGACCAACCTGTCCGACATCATCGAGAAGGAGACTGGCAAACAGTTGGTGATCCAGGAGAGCATCCTGATGCTGCCGGAGGAGGTGGAGGAGGTAATCGGCAACAAGCCCGAGAGTGACATATTGGTCCATACTGCGTACGACGAATCCACCGACGAGAACGTGATGCTGCTGACCAGCGATGCTCCAGAGTACAAGCCGTGGGCCCTCGTCATCCAGGACTCAAATGGGGAGAACAAGATCAAAATGCTATGATAG | MGDPKKKRKVIDIADLRTLGYSQQQQEKIKPKVRSTVAQHHEALVGHGFTHAHIVALSQHPAALGTVAVKYQDMIAALPEATHEAIVGVGKQWSGARALEALLTVAGELRGPPLQLDTGQLLKIAKRGGVTAVEAVHAWRNALTGAPLNLTPEQVVAIASHDGGKQALETVQRLLPVLCQAHGLTPQQVVAIASNGGGKQALETVQRLLPVLCQAHGLTPEQVVAIASNIGGKQALETVQALLPVLCQAHGLTPEQVVAIASNIGGKQALETVQALLPVLCQAHGLTPQQVVAIASNNGGKQALETVQRLLPVLCQAHGLTPEQVVAIASNIGGKQALETVQALLPVLCQAHGLTPEQVVAIASNIGGKQALETVQALLPVLCQAHGLTPQQVVAIASNNGGKQALETVQRLLPVLCQAHGLTPQQVVAIASNGGGKQALETVQRLLPVLCQAHGLTPQQVVAIASNGGGKQALETVQRLLPVLCQAHGLTPEQVVAIASHDGGKQALETVQRLLPVLCQAHGLTPEQVVAIASHDGGKQALETVQRLLPVLCQAHGLTPQQVVAIASNGGGKQALETVQRLLPVLCQAHGLTPQQVVAIASNNGGKQALETVQRLLPVLCQAHGLTPEQVVAIASHDGGKQALETVQRLLPVLCQAHGLTPQQVVAIASNGGGRPALESIVAQLSRPDPSGSGSGGGSGSYALGPYQISAPQLPAYNGQTVGTFYYVNDAGGLESKVFSSGGPTPYPNYANAGHVEGQSALFMRDNGISEGLVFHNNPEGTCGFCVNMTETLLPENAKMTVVPPEGSGGSTNLSDIIEKETGKQLVIQESILMLPEEVEEVIGNKPESDILVHTAYDESTDENVMLLTSDAPEYKPWALVIQDSNGENKIKML |
| pCLS36495 | ATGGGCGATCCTAAAAAGAAACGTAAGGTCATCGATATCGCCGATCTACGCACGCTCGGCTACAGCCAGCAGCAACAGGAGAAGATCAAACCGAAGGTTCGTTCGACAGTGGCGCAGCACCACGAGGCACTGGTCGGCCACGGGTTTACACACGCGCACATCGTTGCGTTAAGCCAACACCCGGCAGCGTTAGGGACCGTCGCTGTCAAGTATCAGGACATGATCGCAGCGTTGCCAGAGGCGACACACGAAGCGATCGTTGGCGTCGGCAAACAGTGGTCCGGCGCACGCGCTCTGGAGGCCTTGCTCACGGTGGCGGGAGAGTTGAGAGGTCCACCGTTACAGTTGGACACAGGCCAACTTCTCAAGATTGCAAAACGTGGCGGCGTGACCGCAGTGGAGGCAGTGCATGCATGGCGCAATGCACTGACGGGTGCCCCGCTCAACTTGACCCCCCAGCAGGTGGTGGCCATCGCCAGCAATAATGGTGGCAAGCAGGCGCTGGAGACGGTCCAGCGGCTGTTGCCGGTGCTGTGCCAGGCCCACGGCTTGACCCCCCAGCAGGTGGTGGCCATCGCCAGCAATGGCGGTGGCAAGCAGGCGCTGGAGACGGTCCAGCGGCTGTTGCCGGTGCTGTGCCAGGCCCACGGCTTGACCCCGGAGCAGGTGGTGGCCATCGCCAGCCACGATGGCGGCAAGCAGGCGCTGGAGACGGTCCAGCGGCTGTTGCCGGTGCTGTGCCAGGCCCACGGCTTGACCCCCCAGCAGGTGGTGGCCATCGCCAGCAATGGCGGTGGCAAGCAGGCGCTGGAGACGGTCCAGCGGCTGTTGCCGGTGCTGTGCCAGGCCCACGGCTTGACCCCGGAGCAGGTGGTGGCCATCGCCAGCCACGATGGCGGCAAGCAGGCGCTGGAGACGGTCCAGCGGCTGTTGCCGGTGCTGTGCCAGGCCCACGGCTTGACCCCCCAGCAGGTGGTGGCCATCGCCAGCAATGGCGGTGGCAAGCAGGCGCTGGAGACGGTCCAGCGGCTGTTGCCGGTGCTGTGCCAGGCCCACGGCTTGACCCCGGAGCAGGTGGTGGCCATCGCCAGCAATATTGGTGGCAAGCAGGCGCTGGAGACGGTGCAGGCGCTGTTGCCGGTGCTGTGCCAGGCCCACGGCTTGACCCCGGAGCAGGTGGTGGCCATCGCCAGCAATATTGGTGGCAAGCAGGCGCTGGAGACGGTGCAGGCGCTGTTGCCGGTGCTGTGCCAGGCCCACGGCTTGACCCCGGAGCAGGTGGTGGCCATCGCCAGCCACGATGGCGGCAAGCAGGCGCTGGAGACGGTCCAGCGGCTGTTGCCGGTGCTGTGCCAGGCCCACGGCTTGACCCCGGAGCAGGTGGTGGCCATCGCCAGCCACGATGGCGGCAAGCAGGCGCTGGAGACGGTCCAGCGGCTGTTGCCGGTGCTGTGCCAGGCCCACGGCTTGACCCCGGAGCAGGTGGTGGCCATCGCCAGCAATATTGGTGGCAAGCAGGCGCTGGAGACGGTGCAGGCGCTGTTGCCGGTGCTGTGCCAGGCCCACGGCTTGACCCCGGAGCAGGTGGTGGCCATCGCCAGCCACGATGGCGGCAAGCAGGCGCTGGAGACGGTCCAGCGGCTGTTGCCGGTGCTGTGCCAGGCCCACGGCTTGACCCCGGAGCAGGTGGTGGCCATCGCCAGCAATATTGGTGGCAAGCAGGCGCTGGAGACGGTGCAGGCGCTGTTGCCGGTGCTGTGCCAGGCCCACGGCTTGACCCCCCAGCAGGTGGTGGCCATCGCCAGCAATGGCGGTGGCAAGCAGGCGCTGGAGACGGTCCAGCGGCTGTTGCCGGTGCTGTGCCAGGCCCACGGCTTGACCCCCCAGCAGGTGGTGGCCATCGCCAGCAATGGCGGTGGCAAGCAGGCGCTGGAGACGGTCCAGCGGCTGTTGCCGGTGCTGTGCCAGGCCCACGGCTTGACCCCTCAGCAGGTGGTGGCCATCGCCAGCAATGGCGGCGGCAGGCCGGCGCTGGAGAGCATTGTTGCCCAGTTATCTCGCCCTGATCCGGCGTTGGCCGCGTTGACCAACGACCACCTCGTCGCCTTGGCCTGCCTCGGCGGGCGTCCTGCGCTGGATGCAGTGAAAAAGGGATTGGGGGGATCTGCCATCCCCGTGAAGCGCGGTGCTACGGGCGAGACTAAGGTGTTCACCGGCAACAGCAACTCCCCCAAATCCCCAACTAAAGGCGGGTGCTCTGGCGGCTCCACCAACCTGAGCGATATTATTGAGAAGGAGACTGGCAAGCAGCTGGTGATCCAGGAGAGCATCCTGATGCTACCCGAGGAGGTGGAGGAGGTTATCGGTAACAAGCCTGAAAGTGACATCTTGGTCCACACAGCCTACGATGAATCGACCGACGAGAACGTGATGCTGCTGACCTCCGACGCACCTGAGTACAAGCCGTGGGCGCTCGTCATCCAGGACTCAAATGGGGAGAACAAGATCAAGATGCTTTGATAG | MGDPKKKRKVIDIADLRTLGYSQQQQEKIKPKVRSTVAQHHEALVGHGFTHAHIVALSQHPAALGTVAVKYQDMIAALPEATHEAIVGVGKQWSGARALEALLTVAGELRGPPLQLDTGQLLKIAKRGGVTAVEAVHAWRNALTGAPLNLTPQQVVAIASNNGGKQALETVQRLLPVLCQAHGLTPQQVVAIASNGGGKQALETVQRLLPVLCQAHGLTPEQVVAIASHDGGKQALETVQRLLPVLCQAHGLTPQQVVAIASNGGGKQALETVQRLLPVLCQAHGLTPEQVVAIASHDGGKQALETVQRLLPVLCQAHGLTPQQVVAIASNGGGKQALETVQRLLPVLCQAHGLTPEQVVAIASNIGGKQALETVQALLPVLCQAHGLTPEQVVAIASNIGGKQALETVQALLPVLCQAHGLTPEQVVAIASHDGGKQALETVQRLLPVLCQAHGLTPEQVVAIASHDGGKQALETVQRLLPVLCQAHGLTPEQVVAIASNIGGKQALETVQALLPVLCQAHGLTPEQVVAIASHDGGKQALETVQRLLPVLCQAHGLTPEQVVAIASNIGGKQALETVQALLPVLCQAHGLTPQQVVAIASNGGGKQALETVQRLLPVLCQAHGLTPQQVVAIASNGGGKQALETVQRLLPVLCQAHGLTPQQVVAIASNGGGRPALESIVAQLSRPDPALAALTNDHLVALACLGGRPALDAVKKGLGGSAIPVKRGATGETKVFTGNSNSPKSPTKGGCSGGSTNLSDIIEKETGKQLVIQESILMLPEEVEEVIGNKPESDILVHTAYDESTDENVMLLTSDAPEYKPWALVIQDSNGENKIKML |
| pCLS37633 | ATGGGCGATCCTAAAAAGAAACGTAAGGTCATCGATATCGCCGATCTACGCACGCTCGGCTACAGCCAGCAGCAACAGGAGAAGATCAAACCGAAGGTTCGTTCGACAGTGGCGCAGCACCACGAGGCACTGGTCGGCCACGGGTTTACACACGCGCACATCGTTGCGTTAAGCCAACACCCGGCAGCGTTAGGGACCGTCGCTGTCAAGTATCAGGACATGATCGCAGCGTTGCCAGAGGCGACACACGAAGCGATCGTTGGCGTCGGCAAACAGTGGTCCGGCGCACGCGCTCTGGAGGCCTTGCTCACGGTGGCGGGAGAGTTGAGAGGTCCACCGTTACAGTTGGACACAGGCCAACTTCTCAAGATTGCAAAACGTGGCGGCGTGACCGCAGTGGAGGCAGTGCATGCATGGCGCAATGCACTGACGGGTGCCCCGCTCAACTTGACCCCCCAGCAGGTGGTGGCCATCGCCAGCAATAATGGTGGCAAGCAGGCGCTGGAGACGGTCCAGCGGCTGTTGCCGGTGCTGTGCCAGGCCCACGGCTTGACCCCCCAGCAGGTGGTGGCCATCGCCAGCAATGGCGGTGGCAAGCAGGCGCTGGAGACGGTCCAGCGGCTGTTGCCGGTGCTGTGCCAGGCCCACGGCTTGACCCCGGAGCAGGTGGTGGCCATCGCCAGCCACGATGGCGGCAAGCAGGCGCTGGAGACGGTCCAGCGGCTGTTGCCGGTGCTGTGCCAGGCCCACGGCTTGACCCCCCAGCAGGTGGTGGCCATCGCCAGCAATGGCGGTGGCAAGCAGGCGCTGGAGACGGTCCAGCGGCTGTTGCCGGTGCTGTGCCAGGCCCACGGCTTGACCCCGGAGCAGGTGGTGGCCATCGCCAGCCACGATGGCGGCAAGCAGGCGCTGGAGACGGTCCAGCGGCTGTTGCCGGTGCTGTGCCAGGCCCACGGCTTGACCCCCCAGCAGGTGGTGGCCATCGCCAGCAATGGCGGTGGCAAGCAGGCGCTGGAGACGGTCCAGCGGCTGTTGCCGGTGCTGTGCCAGGCCCACGGCTTGACCCCGGAGCAGGTGGTGGCCATCGCCAGCAATATTGGTGGCAAGCAGGCGCTGGAGACGGTGCAGGCGCTGTTGCCGGTGCTGTGCCAGGCCCACGGCTTGACCCCGGAGCAGGTGGTGGCCATCGCCAGCAATATTGGTGGCAAGCAGGCGCTGGAGACGGTGCAGGCGCTGTTGCCGGTGCTGTGCCAGGCCCACGGCTTGACCCCGGAGCAGGTGGTGGCCATCGCCAGCCACGATGGCGGCAAGCAGGCGCTGGAGACGGTCCAGCGGCTGTTGCCGGTGCTGTGCCAGGCCCACGGCTTGACCCCGGAGCAGGTGGTGGCCATCGCCAGCCACGATGGCGGCAAGCAGGCGCTGGAGACGGTCCAGCGGCTGTTGCCGGTGCTGTGCCAGGCCCACGGCTTGACCCCGGAGCAGGTGGTGGCCATCGCCAGCAATATTGGTGGCAAGCAGGCGCTGGAGACGGTGCAGGCGCTGTTGCCGGTGCTGTGCCAGGCCCACGGCTTGACCCCGGAGCAGGTGGTGGCCATCGCCAGCCACGATGGCGGCAAGCAGGCGCTGGAGACGGTCCAGCGGCTGTTGCCGGTGCTGTGCCAGGCCCACGGCTTGACCCCGGAGCAGGTGGTGGCCATCGCCAGCAATATTGGTGGCAAGCAGGCGCTGGAGACGGTGCAGGCGCTGTTGCCGGTGCTGTGCCAGGCCCACGGCTTGACCCCCCAGCAGGTGGTGGCCATCGCCAGCAATGGCGGTGGCAAGCAGGCGCTGGAGACGGTCCAGCGGCTGTTGCCGGTGCTGTGCCAGGCCCACGGCTTGACCCCCCAGCAGGTGGTGGCCATCGCCAGCAATGGCGGTGGCAAGCAGGCGCTGGAGACGGTCCAGCGGCTGTTGCCGGTGCTGTGCCAGGCCCACGGCTTGACCCCTCAGCAGGTGGTGGCCATCGCCAGCAATGGCGGCGGCAGGCCGGCGCTGGAGGGGGGATCTGCCATCCCCGTGAAGCGCGGTGCTACGGGCGAGACTAAGGTGTTCACCGGCAACAGCAACTCCCCCAAATCCCCAACTAAAGGCGGGTGCTCTGGCGGCTCCACCAACCTGAGCGATATTATTGAGAAGGAGACTGGCAAGCAGCTGGTGATCCAGGAGAGCATCCTGATGCTACCCGAGGAGGTGGAGGAGGTTATCGGTAACAAGCCTGAAAGTGACATCTTGGTCCACACAGCCTACGATGAATCGACCGACGAGAACGTGATGCTGCTGACCTCCGACGCACCTGAGTACAAGCCGTGGGCGCTCGTCATCCAGGACTCAAATGGGGAGAACAAGATCAAGATGCTTTGATAG | MGDPKKKRKVIDIADLRTLGYSQQQQEKIKPKVRSTVAQHHEALVGHGFTHAHIVALSQHPAALGTVAVKYQDMIAALPEATHEAIVGVGKQWSGARALEALLTVAGELRGPPLQLDTGQLLKIAKRGGVTAVEAVHAWRNALTGAPLNLTPQQVVAIASNNGGKQALETVQRLLPVLCQAHGLTPQQVVAIASNGGGKQALETVQRLLPVLCQAHGLTPEQVVAIASHDGGKQALETVQRLLPVLCQAHGLTPQQVVAIASNGGGKQALETVQRLLPVLCQAHGLTPEQVVAIASHDGGKQALETVQRLLPVLCQAHGLTPQQVVAIASNGGGKQALETVQRLLPVLCQAHGLTPEQVVAIASNIGGKQALETVQALLPVLCQAHGLTPEQVVAIASNIGGKQALETVQALLPVLCQAHGLTPEQVVAIASHDGGKQALETVQRLLPVLCQAHGLTPEQVVAIASHDGGKQALETVQRLLPVLCQAHGLTPEQVVAIASNIGGKQALETVQALLPVLCQAHGLTPEQVVAIASHDGGKQALETVQRLLPVLCQAHGLTPEQVVAIASNIGGKQALETVQALLPVLCQAHGLTPQQVVAIASNGGGKQALETVQRLLPVLCQAHGLTPQQVVAIASNGGGKQALETVQRLLPVLCQAHGLTPQQVVAIASNGGGRPALEGGSAIPVKRGATGETKVFTGNSNSPKSPTKGGCSGGSTNLSDIIEKETGKQLVIQESILMLPEEVEEVIGNKPESDILVHTAYDESTDENVMLLTSDAPEYKPWALVIQDSNGENKIKML |
| pCLS37680 | ATGGGCGATCCTAAAAAGAAACGTAAGGTCATCGATATCGCCGATCTACGCACGCTCGGCTACAGCCAGCAGCAACAGGAGAAGATCAAACCGAAGGTTCGTTCGACAGTGGCGCAGCACCACGAGGCACTGGTCGGCCACGGGTTTACACACGCGCACATCGTTGCGTTAAGCCAACACCCGGCAGCGTTAGGGACCGTCGCTGTCAAGTATCAGGACATGATCGCAGCGTTGCCAGAGGCGACACACGAAGCGATCGTTGGCGTCGGCAAACAGTGGTCCGGCGCACGCGCTCTGGAGGCCTTGCTCACGGTGGCGGGAGAGTTGAGAGGTCCACCGTTACAGTTGGACACAGGCCAACTTCTCAAGATTGCAAAACGTGGCGGCGTGACCGCAGTGGAGGCAGTGCATGCATGGCGCAATGCACTGACGGGTGCCCCGCTCAACTTGACCCCCCAGCAGGTGGTGGCCATCGCCAGCAATAATGGTGGCAAGCAGGCGCTGGAGACGGTCCAGCGGCTGTTGCCGGTGCTGTGCCAGGCCCACGGCTTGACCCCCCAGCAGGTGGTGGCCATCGCCAGCAATGGCGGTGGCAAGCAGGCGCTGGAGACGGTCCAGCGGCTGTTGCCGGTGCTGTGCCAGGCCCACGGCTTGACCCCGGAGCAGGTGGTGGCCATCGCCAGCCACGATGGCGGCAAGCAGGCGCTGGAGACGGTCCAGCGGCTGTTGCCGGTGCTGTGCCAGGCCCACGGCTTGACCCCCCAGCAGGTGGTGGCCATCGCCAGCAATGGCGGTGGCAAGCAGGCGCTGGAGACGGTCCAGCGGCTGTTGCCGGTGCTGTGCCAGGCCCACGGCTTGACCCCGGAGCAGGTGGTGGCCATCGCCAGCCACGATGGCGGCAAGCAGGCGCTGGAGACGGTCCAGCGGCTGTTGCCGGTGCTGTGCCAGGCCCACGGCTTGACCCCCCAGCAGGTGGTGGCCATCGCCAGCAATGGCGGTGGCAAGCAGGCGCTGGAGACGGTCCAGCGGCTGTTGCCGGTGCTGTGCCAGGCCCACGGCTTGACCCCGGAGCAGGTGGTGGCCATCGCCAGCAATATTGGTGGCAAGCAGGCGCTGGAGACGGTGCAGGCGCTGTTGCCGGTGCTGTGCCAGGCCCACGGCTTGACCCCGGAGCAGGTGGTGGCCATCGCCAGCAATATTGGTGGCAAGCAGGCGCTGGAGACGGTGCAGGCGCTGTTGCCGGTGCTGTGCCAGGCCCACGGCTTGACCCCGGAGCAGGTGGTGGCCATCGCCAGCCACGATGGCGGCAAGCAGGCGCTGGAGACGGTCCAGCGGCTGTTGCCGGTGCTGTGCCAGGCCCACGGCTTGACCCCGGAGCAGGTGGTGGCCATCGCCAGCCACGATGGCGGCAAGCAGGCGCTGGAGACGGTCCAGCGGCTGTTGCCGGTGCTGTGCCAGGCCCACGGCTTGACCCCGGAGCAGGTGGTGGCCATCGCCAGCAATATTGGTGGCAAGCAGGCGCTGGAGACGGTGCAGGCGCTGTTGCCGGTGCTGTGCCAGGCCCACGGCTTGACCCCGGAGCAGGTGGTGGCCATCGCCAGCCACGATGGCGGCAAGCAGGCGCTGGAGACGGTCCAGCGGCTGTTGCCGGTGCTGTGCCAGGCCCACGGCTTGACCCCGGAGCAGGTGGTGGCCATCGCCAGCAATATTGGTGGCAAGCAGGCGCTGGAGACGGTGCAGGCGCTGTTGCCGGTGCTGTGCCAGGCCCACGGCTTGACCCCCCAGCAGGTGGTGGCCATCGCCAGCAATGGCGGTGGCAAGCAGGCGCTGGAGACGGTCCAGCGGCTGTTGCCGGTGCTGTGCCAGGCCCACGGCTTGACCCCCCAGCAGGTGGTGGCCATCGCCAGCAATGGCGGTGGCAAGCAGGCGCTGGAGACGGTCCAGCGGCTGTTGCCGGTGCTGTGCCAGGCCCACGGCTTGACCCCTCAGCAGGTGGTGGCCATCGCCAGCAATGGCGGCGGCAGGCCGGCGCTGGAGAGCATTGTTGCCCAGTTATCTCGCCCTGATCCGAGTGGCAGCGGAAGTGGCGGGGGATCTGCCATCCCCGTGAAGCGCGGTGCTACGGGCGAGACTAAGGTGTTCACCGGCAACAGCAACTCCCCCAAATCCCCAACTAAAGGCGGGTGCTCTGGCGGCTCCACCAACCTGAGCGATATTATTGAGAAGGAGACTGGCAAGCAGCTGGTGATCCAGGAGAGCATCCTGATGCTACCCGAGGAGGTGGAGGAGGTTATCGGTAACAAGCCTGAAAGTGACATCTTGGTCCACACAGCCTACGATGAATCGACCGACGAGAACGTGATGCTGCTGACCTCCGACGCACCTGAGTACAAGCCGTGGGCGCTCGTCATCCAGGACTCAAATGGGGAGAACAAGATCAAGATGCTTTGATAG | MGDPKKKRKVIDIADLRTLGYSQQQQEKIKPKVRSTVAQHHEALVGHGFTHAHIVALSQHPAALGTVAVKYQDMIAALPEATHEAIVGVGKQWSGARALEALLTVAGELRGPPLQLDTGQLLKIAKRGGVTAVEAVHAWRNALTGAPLNLTPQQVVAIASNNGGKQALETVQRLLPVLCQAHGLTPQQVVAIASNGGGKQALETVQRLLPVLCQAHGLTPEQVVAIASHDGGKQALETVQRLLPVLCQAHGLTPQQVVAIASNGGGKQALETVQRLLPVLCQAHGLTPEQVVAIASHDGGKQALETVQRLLPVLCQAHGLTPQQVVAIASNGGGKQALETVQRLLPVLCQAHGLTPEQVVAIASNIGGKQALETVQALLPVLCQAHGLTPEQVVAIASNIGGKQALETVQALLPVLCQAHGLTPEQVVAIASHDGGKQALETVQRLLPVLCQAHGLTPEQVVAIASHDGGKQALETVQRLLPVLCQAHGLTPEQVVAIASNIGGKQALETVQALLPVLCQAHGLTPEQVVAIASHDGGKQALETVQRLLPVLCQAHGLTPEQVVAIASNIGGKQALETVQALLPVLCQAHGLTPQQVVAIASNGGGKQALETVQRLLPVLCQAHGLTPQQVVAIASNGGGKQALETVQRLLPVLCQAHGLTPQQVVAIASNGGGRPALESIVAQLSRPDPSGSGSGGGSAIPVKRGATGETKVFTGNSNSPKSPTKGGCSGGSTNLSDIIEKETGKQLVIQESILMLPEEVEEVIGNKPESDILVHTAYDESTDENVMLLTSDAPEYKPWALVIQDSNGENKIKML |

**Supplementary Table 5. Nuclear and protein sequences of TRAC TALEN and TRAC target sequence (from left to right: black= TAL array; pink= linker; red= FokI).**

|  | Plasmid | Nucl. Seq. | Prot. Seq. |
| --- | --- | --- | --- |
| TRAC | pCLS24046 | ATGGGCGATCCTAAAAAGAAACGTAAGGTCATCGATATCGCCGATCTACGCACGCTCGGCTACAGCCAGCAGCAACAGGAGAAGATCAAACCGAAGGTTCGTTCGACAGTGGCGCAGCACCACGAGGCACTGGTCGGCCACGGGTTTACACACGCGCACATCGTTGCGTTAAGCCAACACCCGGCAGCGTTAGGGACCGTCGCTGTCAAGTATCAGGACATGATCGCAGCGTTGCCAGAGGCGACACACGAAGCGATCGTTGGCGTCGGCAAACAGTGGTCCGGCGCACGCGCTCTGGAGGCCTTGCTCACGGTGGCGGGAGAGTTGAGAGGTCCACCGTTACAGTTGGACACAGGCCAACTTCTCAAGATTGCAAAACGTGGCGGCGTGACCGCAGTGGAGGCAGTGCATGCATGGCGCAATGCACTGACGGGTGCCCCGCTCAACTTGACCCCCCAGCAGGTGGTGGCCATCGCCAGCAATGGCGGTGGCAAGCAGGCGCTGGAGACGGTCCAGCGGCTGTTGCCGGTGCTGTGCCAGGCCCACGGCTTGACCCCCCAGCAGGTGGTGGCCATCGCCAGCAATAATGGTGGCAAGCAGGCGCTGGAGACGGTCCAGCGGCTGTTGCCGGTGCTGTGCCAGGCCCACGGCTTGACCCCCCAGCAGGTGGTGGCCATCGCCAGCAATGGCGGTGGCAAGCAGGCGCTGGAGACGGTCCAGCGGCTGTTGCCGGTGCTGTGCCAGGCCCACGGCTTGACCCCGGAGCAGGTGGTGGCCATCGCCAGCCACGATGGCGGCAAGCAGGCGCTGGAGACGGTCCAGCGGCTGTTGCCGGTGCTGTGCCAGGCCCACGGCTTGACCCCGGAGCAGGTGGTGGCCATCGCCAGCCACGATGGCGGCAAGCAGGCGCTGGAGACGGTCCAGCGGCTGTTGCCGGTGCTGTGCCAGGCCCACGGCTTGACCCCGGAGCAGGTGGTGGCCATCGCCAGCCACGATGGCGGCAAGCAGGCGCTGGAGACGGTCCAGCGGCTGTTGCCGGTGCTGTGCCAGGCCCACGGCTTGACCCCGGAGCAGGTGGTGGCCATCGCCAGCAATATTGGTGGCAAGCAGGCGCTGGAGACGGTGCAGGCGCTGTTGCCGGTGCTGTGCCAGGCCCACGGCTTGACCCCGGAGCAGGTGGTGGCCATCGCCAGCCACGATGGCGGCAAGCAGGCGCTGGAGACGGTCCAGCGGCTGTTGCCGGTGCTGTGCCAGGCCCACGGCTTGACCCCGGAGCAGGTGGTGGCCATCGCCAGCAATATTGGTGGCAAGCAGGCGCTGGAGACGGTGCAGGCGCTGTTGCCGGTGCTGTGCCAGGCCCACGGCTTGACCCCCCAGCAGGTGGTGGCCATCGCCAGCAATAATGGTGGCAAGCAGGCGCTGGAGACGGTCCAGCGGCTGTTGCCGGTGCTGTGCCAGGCCCACGGCTTGACCCCGGAGCAGGTGGTGGCCATCGCCAGCAATATTGGTGGCAAGCAGGCGCTGGAGACGGTGCAGGCGCTGTTGCCGGTGCTGTGCCAGGCCCACGGCTTGACCCCCCAGCAGGTGGTGGCCATCGCCAGCAATGGCGGTGGCAAGCAGGCGCTGGAGACGGTCCAGCGGCTGTTGCCGGTGCTGTGCCAGGCCCACGGCTTGACCCCGGAGCAGGTGGTGGCCATCGCCAGCAATATTGGTGGCAAGCAGGCGCTGGAGACGGTGCAGGCGCTGTTGCCGGTGCTGTGCCAGGCCCACGGCTTGACCCCCCAGCAGGTGGTGGCCATCGCCAGCAATGGCGGTGGCAAGCAGGCGCTGGAGACGGTCCAGCGGCTGTTGCCGGTGCTGTGCCAGGCCCACGGCTTGACCCCGGAGCAGGTGGTGGCCATCGCCAGCCACGATGGCGGCAAGCAGGCGCTGGAGACGGTCCAGCGGCTGTTGCCGGTGCTGTGCCAGGCCCACGGCTTGACCCCTCAGCAGGTGGTGGCCATCGCCAGCAATGGCGGCGGCAGGCCGGCGCTGGAGAGCATTGTTGCCCAGTTATCTCGCCCTGATCCGGCGTTGGCCGCGTTGACCAACGACCACCTCGTCGCCTTGGCCTGCCTCGGCGGGCGTCCTGCGCTGGATGCAGTGAAAAAGGGATTGGGGGATCCTATCAGCCGTTCCCAGCTGGTGAAGTCCGAGCTGGAGGAGAAGAAATCCGAGTTGAGGCACAAGCTGAAGTACGTGCCCCACGAGTACATCGAGCTGATCGAGATCGCCCGGAACAGCACCCAGGACCGTATCCTGGAGATGAAGGTGATGGAGTTCTTCATGAAGGTGTACGGCTACAGGGGCAAGCACCTGGGCGGCTCCAGGAAGCCCGACGGCGCCATCTACACCGTGGGCTCCCCCATCGACTACGGCGTGATCGTGGACACCAAGGCCTACTCCGGCGGCTACAACCTGCCCATCGGCCAGGCCGACGAAATGCAGAGGTACGTGGAGGAGAACCAGACCAGGAACAAGCACATCAACCCCAACGAGTGGTGGAAGGTGTACCCCTCCAGCGTGACCGAGTTCAAGTTCCTGTTCGTGTCCGGCCACTTCAAGGGCAACTACAAGGCCCAGCTGACCAGGCTGAACCACATCACCAACTGCAACGGCGCCGTGCTGTCCGTGGAGGAGCTCCTGATCGGCGGCGAGATGATCAAGGCCGGCACCCTGACCCTGGAGGAGGTGAGGAGGAAGTTCAACAACGGCGAGATCAACTTCGCGGCCGACTGATAA | MGDPKKKRKVIDIADLRTLGYSQQQQEKIKPKVRSTVAQHHEALVGHGFTHAHIVALSQHPAALGTVAVKYQDMIAALPEATHEAIVGVGKQWSGARALEALLTVAGELRGPPLQLDTGQLLKIAKRGGVTAVEAVHAWRNALTGAPLNLTPQQVVAIASNGGGKQALETVQRLLPVLCQAHGLTPQQVVAIASNNGGKQALETVQRLLPVLCQAHGLTPQQVVAIASNGGGKQALETVQRLLPVLCQAHGLTPEQVVAIASHDGGKQALETVQRLLPVLCQAHGLTPEQVVAIASHDGGKQALETVQRLLPVLCQAHGLTPEQVVAIASHDGGKQALETVQRLLPVLCQAHGLTPEQVVAIASNIGGKQALETVQALLPVLCQAHGLTPEQVVAIASHDGGKQALETVQRLLPVLCQAHGLTPEQVVAIASNIGGKQALETVQALLPVLCQAHGLTPQQVVAIASNNGGKQALETVQRLLPVLCQAHGLTPEQVVAIASNIGGKQALETVQALLPVLCQAHGLTPQQVVAIASNGGGKQALETVQRLLPVLCQAHGLTPEQVVAIASNIGGKQALETVQALLPVLCQAHGLTPQQVVAIASNGGGKQALETVQRLLPVLCQAHGLTPEQVVAIASHDGGKQALETVQRLLPVLCQAHGLTPQQVVAIASNGGGRPALESIVAQLSRPDPALAALTNDHLVALACLGGRPALDAVKKGLGDPISRSQLVKSELEEKKSELRHKLKYVPHEYIELIEIARNSTQDRILEMKVMEFFMKVYGYRGKHLGGSRKPDGAIYTVGSPIDYGVIVDTKAYSGGYNLPIGQADEMQRYVEENQTRNKHINPNEWWKVYPSSVTEFKFLFVSGHFKGNYKAQLTRLNHITNCNGAVLSVEELLIGGEMIKAGTLTLEEVRRKFNNGEINFAAD |
|  | pCLS24048 | ATGGGCGATCCTAAAAAGAAACGTAAGGTCATCGATATCGCCGATCTACGCACGCTCGGCTACAGCCAGCAGCAACAGGAGAAGATCAAACCGAAGGTTCGTTCGACAGTGGCGCAGCACCACGAGGCACTGGTCGGCCACGGGTTTACACACGCGCACATCGTTGCGTTAAGCCAACACCCGGCAGCGTTAGGGACCGTCGCTGTCAAGTATCAGGACATGATCGCAGCGTTGCCAGAGGCGACACACGAAGCGATCGTTGGCGTCGGCAAACAGTGGTCCGGCGCACGCGCTCTGGAGGCCTTGCTCACGGTGGCGGGAGAGTTGAGAGGTCCACCGTTACAGTTGGACACAGGCCAACTTCTCAAGATTGCAAAACGTGGCGGCGTGACCGCAGTGGAGGCAGTGCATGCATGGCGCAATGCACTGACGGGTGCCCCGCTCAACTTGACCCCGGAGCAGGTGGTGGCCATCGCCAGCCACGATGGCGGCAAGCAGGCGCTGGAGACGGTCCAGCGGCTGTTGCCGGTGCTGTGCCAGGCCCACGGCTTGACCCCCCAGCAGGTGGTGGCCATCGCCAGCAATGGCGGTGGCAAGCAGGCGCTGGAGACGGTCCAGCGGCTGTTGCCGGTGCTGTGCCAGGCCCACGGCTTGACCCCGGAGCAGGTGGTGGCCATCGCCAGCCACGATGGCGGCAAGCAGGCGCTGGAGACGGTCCAGCGGCTGTTGCCGGTGCTGTGCCAGGCCCACGGCTTGACCCCGGAGCAGGTGGTGGCCATCGCCAGCAATATTGGTGGCAAGCAGGCGCTGGAGACGGTGCAGGCGCTGTTGCCGGTGCTGTGCCAGGCCCACGGCTTGACCCCCCAGCAGGTGGTGGCCATCGCCAGCAATAATGGTGGCAAGCAGGCGCTGGAGACGGTCCAGCGGCTGTTGCCGGTGCTGTGCCAGGCCCACGGCTTGACCCCGGAGCAGGTGGTGGCCATCGCCAGCCACGATGGCGGCAAGCAGGCGCTGGAGACGGTCCAGCGGCTGTTGCCGGTGCTGTGCCAGGCCCACGGCTTGACCCCCCAGCAGGTGGTGGCCATCGCCAGCAATGGCGGTGGCAAGCAGGCGCTGGAGACGGTCCAGCGGCTGTTGCCGGTGCTGTGCCAGGCCCACGGCTTGACCCCCCAGCAGGTGGTGGCCATCGCCAGCAATAATGGTGGCAAGCAGGCGCTGGAGACGGTCCAGCGGCTGTTGCCGGTGCTGTGCCAGGCCCACGGCTTGACCCCCCAGCAGGTGGTGGCCATCGCCAGCAATAATGGTGGCAAGCAGGCGCTGGAGACGGTCCAGCGGCTGTTGCCGGTGCTGTGCCAGGCCCACGGCTTGACCCCCCAGCAGGTGGTGGCCATCGCCAGCAATGGCGGTGGCAAGCAGGCGCTGGAGACGGTCCAGCGGCTGTTGCCGGTGCTGTGCCAGGCCCACGGCTTGACCCCGGAGCAGGTGGTGGCCATCGCCAGCAATATTGGTGGCAAGCAGGCGCTGGAGACGGTGCAGGCGCTGTTGCCGGTGCTGTGCCAGGCCCACGGCTTGACCCCGGAGCAGGTGGTGGCCATCGCCAGCCACGATGGCGGCAAGCAGGCGCTGGAGACGGTCCAGCGGCTGTTGCCGGTGCTGTGCCAGGCCCACGGCTTGACCCCGGAGCAGGTGGTGGCCATCGCCAGCAATATTGGTGGCAAGCAGGCGCTGGAGACGGTGCAGGCGCTGTTGCCGGTGCTGTGCCAGGCCCACGGCTTGACCCCGGAGCAGGTGGTGGCCATCGCCAGCCACGATGGCGGCAAGCAGGCGCTGGAGACGGTCCAGCGGCTGTTGCCGGTGCTGTGCCAGGCCCACGGCTTGACCCCCCAGCAGGTGGTGGCCATCGCCAGCAATAATGGTGGCAAGCAGGCGCTGGAGACGGTCCAGCGGCTGTTGCCGGTGCTGTGCCAGGCCCACGGCTTGACCCCTCAGCAGGTGGTGGCCATCGCCAGCAATGGCGGCGGCAGGCCGGCGCTGGAGAGCATTGTTGCCCAGTTATCTCGCCCTGATCCGGCGTTGGCCGCGTTGACCAACGACCACCTCGTCGCCTTGGCCTGCCTCGGCGGGCGTCCTGCGCTGGATGCAGTGAAAAAGGGATTGGGGGATCCTATCAGCCGTTCCCAGCTGGTGAAGTCCGAGCTGGAGGAGAAGAAATCCGAGTTGAGGCACAAGCTGAAGTACGTGCCCCACGAGTACATCGAGCTGATCGAGATCGCCCGGAACAGCACCCAGGACCGTATCCTGGAGATGAAGGTGATGGAGTTCTTCATGAAGGTGTACGGCTACAGGGGCAAGCACCTGGGCGGCTCCAGGAAGCCCGACGGCGCCATCTACACCGTGGGCTCCCCCATCGACTACGGCGTGATCGTGGACACCAAGGCCTACTCCGGCGGCTACAACCTGCCCATCGGCCAGGCCGACGAAATGCAGAGGTACGTGGAGGAGAACCAGACCAGGAACAAGCACATCAACCCCAACGAGTGGTGGAAGGTGTACCCCTCCAGCGTGACCGAGTTCAAGTTCCTGTTCGTGTCCGGCCACTTCAAGGGCAACTACAAGGCCCAGCTGACCAGGCTGAACCACATCACCAACTGCAACGGCGCCGTGCTGTCCGTGGAGGAGCTCCTGATCGGCGGCGAGATGATCAAGGCCGGCACCCTGACCCTGGAGGAGGTGAGGAGGAAGTTCAACAACGGCGAGATCAACTTCGCGGCCGACTGATAA | MGDPKKKRKVIDIADLRTLGYSQQQQEKIKPKVRSTVAQHHEALVGHGFTHAHIVALSQHPAALGTVAVKYQDMIAALPEATHEAIVGVGKQWSGARALEALLTVAGELRGPPLQLDTGQLLKIAKRGGVTAVEAVHAWRNALTGAPLNLTPEQVVAIASHDGGKQALETVQRLLPVLCQAHGLTPQQVVAIASNGGGKQALETVQRLLPVLCQAHGLTPEQVVAIASHDGGKQALETVQRLLPVLCQAHGLTPEQVVAIASNIGGKQALETVQALLPVLCQAHGLTPQQVVAIASNNGGKQALETVQRLLPVLCQAHGLTPEQVVAIASHDGGKQALETVQRLLPVLCQAHGLTPQQVVAIASNGGGKQALETVQRLLPVLCQAHGLTPQQVVAIASNNGGKQALETVQRLLPVLCQAHGLTPQQVVAIASNNGGKQALETVQRLLPVLCQAHGLTPQQVVAIASNGGGKQALETVQRLLPVLCQAHGLTPEQVVAIASNIGGKQALETVQALLPVLCQAHGLTPEQVVAIASHDGGKQALETVQRLLPVLCQAHGLTPEQVVAIASNIGGKQALETVQALLPVLCQAHGLTPEQVVAIASHDGGKQALETVQRLLPVLCQAHGLTPQQVVAIASNNGGKQALETVQRLLPVLCQAHGLTPQQVVAIASNGGGRPALESIVAQLSRPDPALAALTNDHLVALACLGGRPALDAVKKGLGDPISRSQLVKSELEEKKSELRHKLKYVPHEYIELIEIARNSTQDRILEMKVMEFFMKVYGYRGKHLGGSRKPDGAIYTVGSPIDYGVIVDTKAYSGGYNLPIGQADEMQRYVEENQTRNKHINPNEWWKVYPSSVTEFKFLFVSGHFKGNYKAQLTRLNHITNCNGAVLSVEELLIGGEMIKAGTLTLEEVRRKFNNGEINFAAD |
|  |  |  |  |
| TRAC target | | TGATCCTCTTGTCCCACAGATATCCagaaccctgaccctgCCGTGTACCAGCTGAGAGA | |

**Supplementary Table 6. Linear regression analysis for C40 and C11 scaffolds on 15 and 13bp spacers. From left to right: posM1[T.C] = degree of the variation from the A (reference) when a C is in position M1; coef =coefficient of the effect of the change from A (reference) to X (other nucleotide); t= value of the t-statistic (estimated value for a particular coefficient divided by the standard error); P>|t| =p-value of the test statistically significance; [0.025-0.975] lower and higher value for the 95% confidence interval.**

|  | coef | std err | t | P>\|t\| | [0.025 | 0.975] |
| --- | --- | --- | --- | --- | --- | --- |
| **C40-15bp** |  |  |  |  |  |  |
| Intercept | 44.9456 | 2.326 | 19.323 | 0 | 40.364 | 49.527 |
| posM1[T.C] | -8.2423 | 1.802 | -4.575 | 0 | -11.791 | -4.693 |
| posM1[T.G] | 16.2511 | 1.802 | 9.02 | 0 | 12.702 | 19.8 |
| posM1[T.T] | -10.5146 | 1.809 | -5.812 | 0 | -14.078 | -6.951 |
| posM2[T.C] | 20.4983 | 1.809 | 11.331 | 0 | 16.935 | 24.062 |
| posM2[T.G] | 13.1274 | 1.809 | 7.256 | 0 | 9.564 | 16.691 |
| posM2[T.T] | 1.0072 | 1.809 | 0.557 | 0.578 | -2.556 | 4.571 |
| pos1[T.C] | 13.6364 | 1.809 | 7.538 | 0 | 10.073 | 17.2 |
| pos1[T.G] | -5.0569 | 1.809 | -2.795 | 0.006 | -8.62 | -1.493 |
| pos1[T.T] | -14.9153 | 1.809 | -8.245 | 0 | -18.479 | -11.352 |
| pos2[T.C] | -5.9195 | 1.809 | -3.272 | 0.001 | -9.483 | -2.356 |
| pos2[T.G] | -1.543 | 1.809 | -0.853 | 0.395 | -5.107 | 2.021 |
| pos2[T.T] | -7.3443 | 1.809 | -4.06 | 0 | -10.908 | -3.781 |
|  |  |  |  |  |  |  |
|  |  |  |  |  |  |  |
| **C11-15bp** |  |  |  |  |  |  |
| Intercept | 30.0684 | 2.706 | 11.112 | 0 | 24.738 | 35.399 |
| posM1[T.C] | -9.9162 | 2.123 | -4.671 | 0 | -14.098 | -5.735 |
| posM1[T.G] | 24.2565 | 2.123 | 11.427 | 0 | 20.075 | 28.438 |
| posM1[T.T] | -12.1225 | 2.123 | -5.711 | 0 | -16.304 | -7.941 |
| posM2[T.C] | 23.8783 | 2.123 | 11.249 | 0 | 19.697 | 28.06 |
| posM2[T.G] | 12.8871 | 2.123 | 6.071 | 0 | 8.706 | 17.068 |
| posM2[T.T] | -1.5909 | 2.123 | -0.749 | 0.454 | -5.772 | 2.591 |
| pos1[T.C] | 15.5023 | 2.123 | 7.303 | 0 | 11.321 | 19.684 |
| pos1[T.G] | -9.8549 | 2.123 | -4.642 | 0 | -14.036 | -5.673 |
| pos1[T.T] | -14.0967 | 2.123 | -6.641 | 0 | -18.278 | -9.915 |
| pos2[T.C] | -5.8664 | 2.123 | -2.764 | 0.006 | -10.048 | -1.685 |
| pos2[T.G] | -1.9236 | 2.123 | -0.906 | 0.366 | -6.105 | 2.258 |
| pos2[T.T] | -8.2863 | 2.123 | -3.903 | 0 | -12.468 | -4.105 |
|  |  |  |  |  |  |  |
| **C40-13bp** |  |  |  |  |  |  |
| Intercept | 67.8601 | 2.033 | 33.372 | 0 | 63.855 | 71.865 |
| posM1[T.C] | -7.6506 | 1.595 | -4.796 | 0 | -10.793 | -4.509 |
| posM1[T.G] | 6.8841 | 1.595 | 4.316 | 0 | 3.742 | 10.026 |
| posM1[T.T] | -12.3052 | 1.595 | -7.714 | 0 | -15.447 | -9.163 |
| posM2[T.C] | 12.6119 | 1.595 | 7.906 | 0 | 9.47 | 15.754 |
| posM2[T.G] | 12.8566 | 1.595 | 8.06 | 0 | 9.715 | 15.999 |
| posM2[T.T] | -5.6163 | 1.595 | -3.521 | 0.001 | -8.758 | -2.474 |
| pos1[T.C] | 5.4272 | 1.595 | 3.402 | 0.001 | 2.285 | 8.569 |
| pos1[T.G] | -3.0078 | 1.595 | -1.886 | 0.061 | -6.15 | 0.134 |
| pos1[T.T] | -10.5141 | 1.595 | -6.591 | 0 | -13.656 | -7.372 |
| pos2[T.C] | -1.4694 | 1.595 | -0.921 | 0.358 | -4.611 | 1.673 |
| pos2[T.G] | 2.2578 | 1.595 | 1.415 | 0.158 | -0.884 | 5.4 |
| pos2[T.T] | -4.1093 | 1.595 | -2.576 | 0.011 | -7.251 | -0.967 |
|  |  |  |  |  |  |  |
| **C11-13bp** |  |  |  |  |  |  |
| Intercept | 77.7486 | 1.35 | 57.608 | 0 | 75.09 | 80.407 |
| posM1[T.C] | -2.1022 | 1.059 | -1.986 | 0.048 | -4.187 | -0.017 |
| posM1[T.G] | 4.1371 | 1.059 | 3.908 | 0 | 2.052 | 6.222 |
| posM1[T.T] | -6.6354 | 1.063 | -6.243 | 0 | -8.729 | -4.542 |
| posM2[T.C] | 8.7759 | 1.063 | 8.256 | 0 | 6.682 | 10.87 |
| posM2[T.G] | 4.3575 | 1.063 | 4.1 | 0 | 2.264 | 6.451 |
| posM2[T.T] | -2.8365 | 1.063 | -2.669 | 0.008 | -4.93 | -0.743 |
| pos1[T.C] | 2.8949 | 1.059 | 2.735 | 0.007 | 0.81 | 4.98 |
| pos1[T.G] | -2.9512 | 1.063 | -2.777 | 0.006 | -5.045 | -0.857 |
| pos1[T.T] | -6.2502 | 1.059 | -5.904 | 0 | -8.335 | -4.165 |
| pos2[T.C] | 0.3331 | 1.059 | 0.315 | 0.753 | -1.752 | 2.418 |
| pos2[T.G] | 2.3728 | 1.059 | 2.242 | 0.026 | 0.288 | 4.458 |
| pos2[T.T] | -2.3533 | 1.063 | -2.214 | 0.028 | -4.447 | -0.26 |
