# Supplementary material for: Comprehensive analysis of the editing window of C-to-T TALE base editors
Source: Sci Rep. 2024 Jun 4;14:12870. doi: 10.1038/s41598-024-63203-8 (PMC11150444; doi:10.1038/s41598-024-63203-8)
Supplement: Supplementary file 2 — Supplementary Figures. [file 41598_2024_63203_MOESM2_ESM.pptx]

## Slide 1
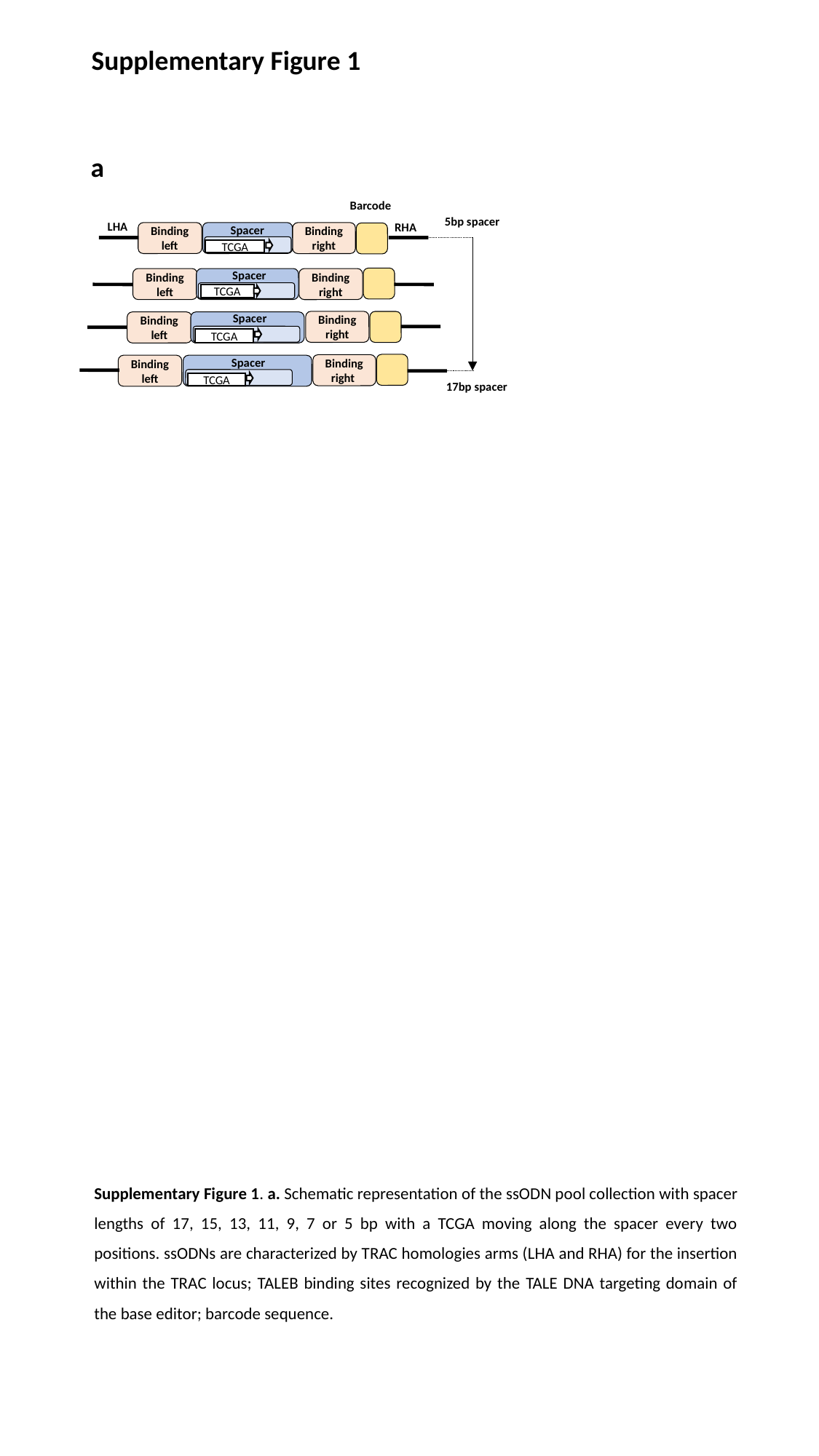

Supplementary Figure 1
a
Barcode
5bp spacer
LHA
RHA
Spacer
Binding left
Binding right
TCGA
Spacer
Binding left
Binding right
TCGA
Spacer
Binding right
Binding left
TCGA
Spacer
Binding right
Binding left
TCGA
17bp spacer
Supplementary Figure 1. a. Schematic representation of the ssODN pool collection with spacer lengths of 17, 15, 13, 11, 9, 7 or 5 bp with a TCGA moving along the spacer every two positions. ssODNs are characterized by TRAC homologies arms (LHA and RHA) for the insertion within the TRAC locus; TALEB binding sites recognized by the TALE DNA targeting domain of the base editor; barcode sequence.

## Slide 2
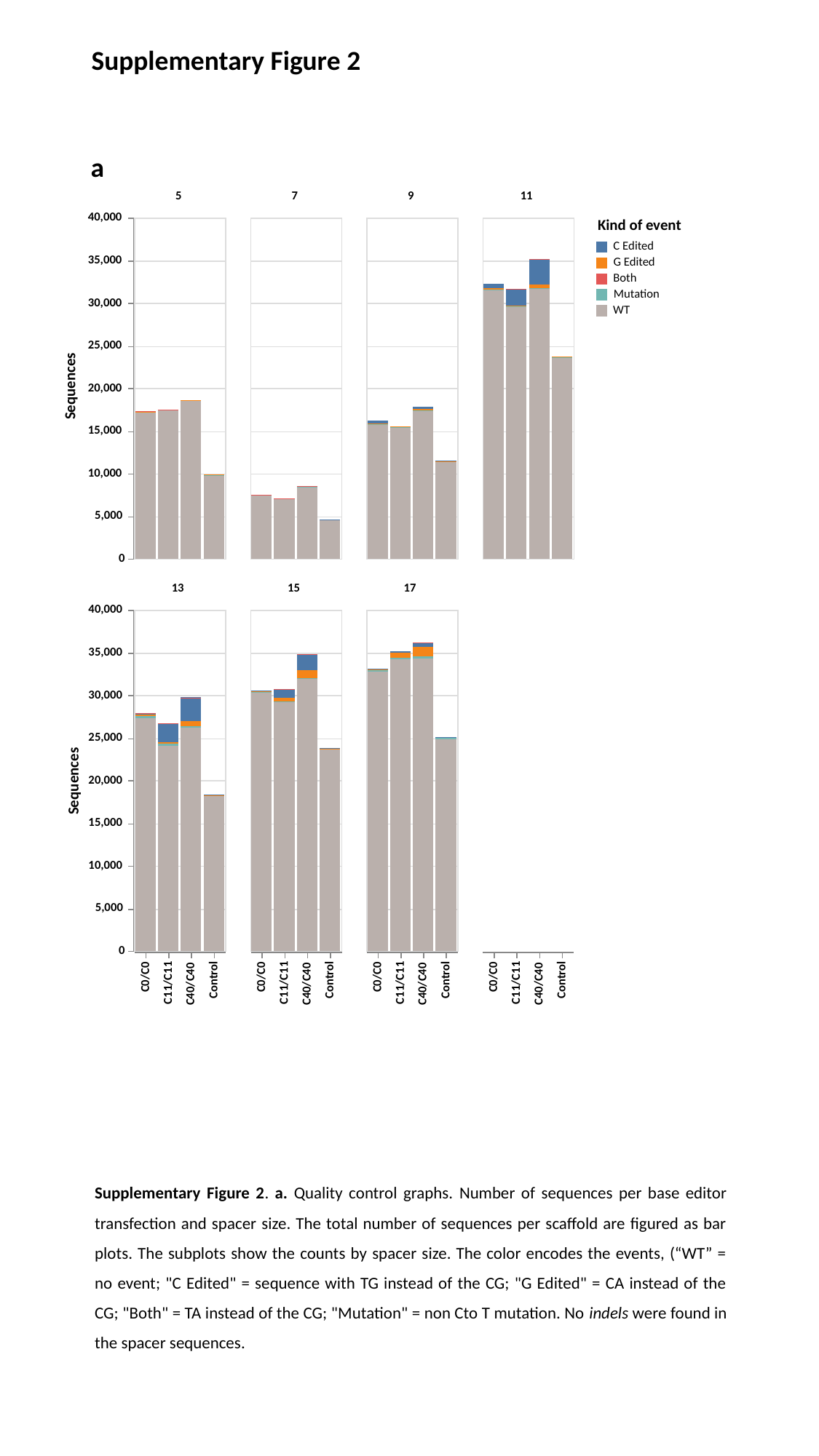

Supplementary Figure 2
a
5
7
9
11
13
15
17
40,000
35,000
30,000
25,000
20,000
15,000
10,000
5,000
0
Sequences
Kind of event
C Edited
G Edited
Both
Mutation
WT
40,000
35,000
30,000
25,000
20,000
15,000
10,000
5,000
0
Sequences
C0/C0
Control
C11/C11
C40/C40
C0/C0
Control
C11/C11
C40/C40
C0/C0
Control
C11/C11
C40/C40
C0/C0
Control
C11/C11
C40/C40
Supplementary Figure 2. a. Quality control graphs. Number of sequences per base editor transfection and spacer size. The total number of sequences per scaffold are figured as bar plots. The subplots show the counts by spacer size. The color encodes the events, (“WT” = no event; "C Edited" = sequence with TG instead of the CG; "G Edited" = CA instead of the CG; "Both" = TA instead of the CG; "Mutation" = non Cto T mutation. No indels were found in the spacer sequences.

## Slide 3
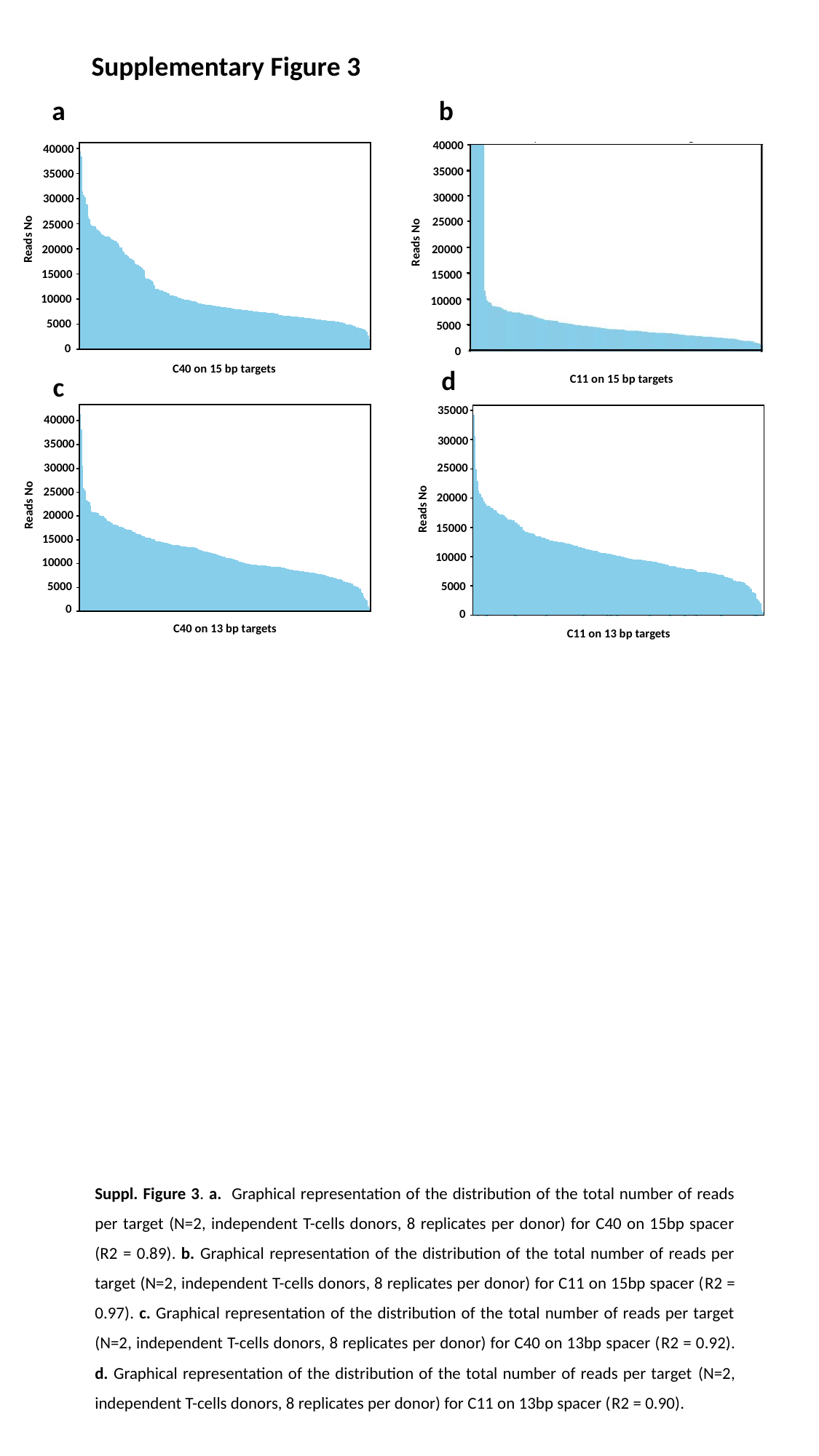

Supplementary Figure 3
a
b
40000
35000
30000
25000
20000
15000
10000
5000
0
Reads No
C11 on 15 bp targets
40000
35000
30000
25000
20000
15000
10000
5000
0
Reads No
C40 on 15 bp targets
d
c
40000
35000
30000
25000
20000
15000
10000
5000
0
Reads No
C40 on 13 bp targets
35000
30000
25000
20000
15000
10000
5000
0
Reads No
C11 on 13 bp targets
Suppl. Figure 3. a. Graphical representation of the distribution of the total number of reads per target (N=2, independent T-cells donors, 8 replicates per donor) for C40 on 15bp spacer (R2 = 0.89). b. Graphical representation of the distribution of the total number of reads per target (N=2, independent T-cells donors, 8 replicates per donor) for C11 on 15bp spacer (R2 = 0.97). c. Graphical representation of the distribution of the total number of reads per target (N=2, independent T-cells donors, 8 replicates per donor) for C40 on 13bp spacer (R2 = 0.92). d. Graphical representation of the distribution of the total number of reads per target (N=2, independent T-cells donors, 8 replicates per donor) for C11 on 13bp spacer (R2 = 0.90).

## Slide 4
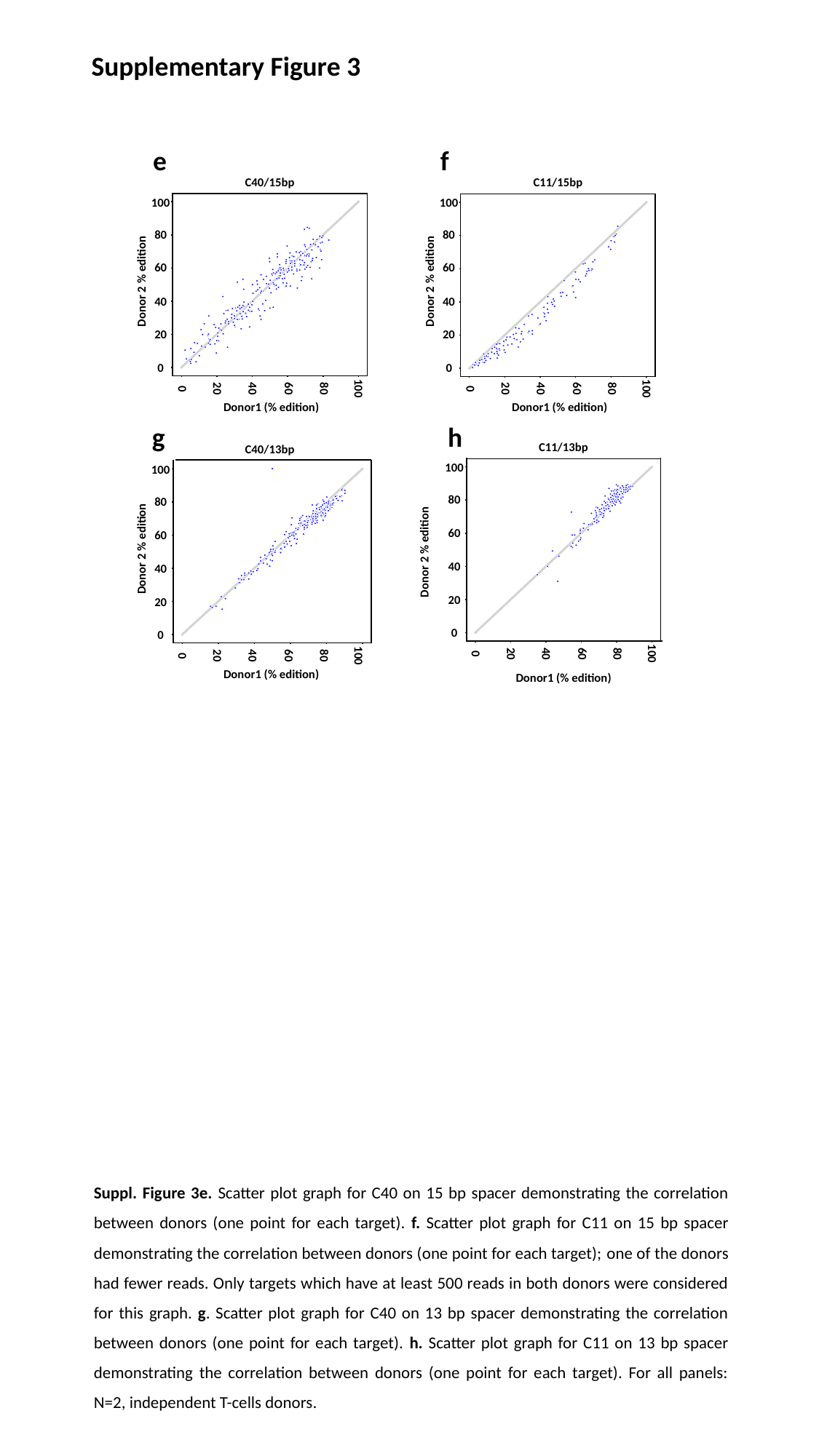

Supplementary Figure 3
e
f
C40/15bp
100
80
60
40
20
0
Donor 2 % edition
100
80
60
40
20
0
Donor1 (% edition)
C11/15bp
100
80
60
40
20
0
Donor 2 % edition
100
80
60
40
20
0
Donor1 (% edition)
g
h
C11/13bp
100
80
60
40
20
0
100
80
60
40
20
0
Donor 2 % edition
Donor1 (% edition)
C40/13bp
100
80
60
40
20
0
Donor 2 % edition
100
80
60
40
20
0
Donor1 (% edition)
Suppl. Figure 3e. Scatter plot graph for C40 on 15 bp spacer demonstrating the correlation between donors (one point for each target). f. Scatter plot graph for C11 on 15 bp spacer demonstrating the correlation between donors (one point for each target); one of the donors had fewer reads. Only targets which have at least 500 reads in both donors were considered for this graph. g. Scatter plot graph for C40 on 13 bp spacer demonstrating the correlation between donors (one point for each target). h. Scatter plot graph for C11 on 13 bp spacer demonstrating the correlation between donors (one point for each target). For all panels: N=2, independent T-cells donors.

## Slide 5
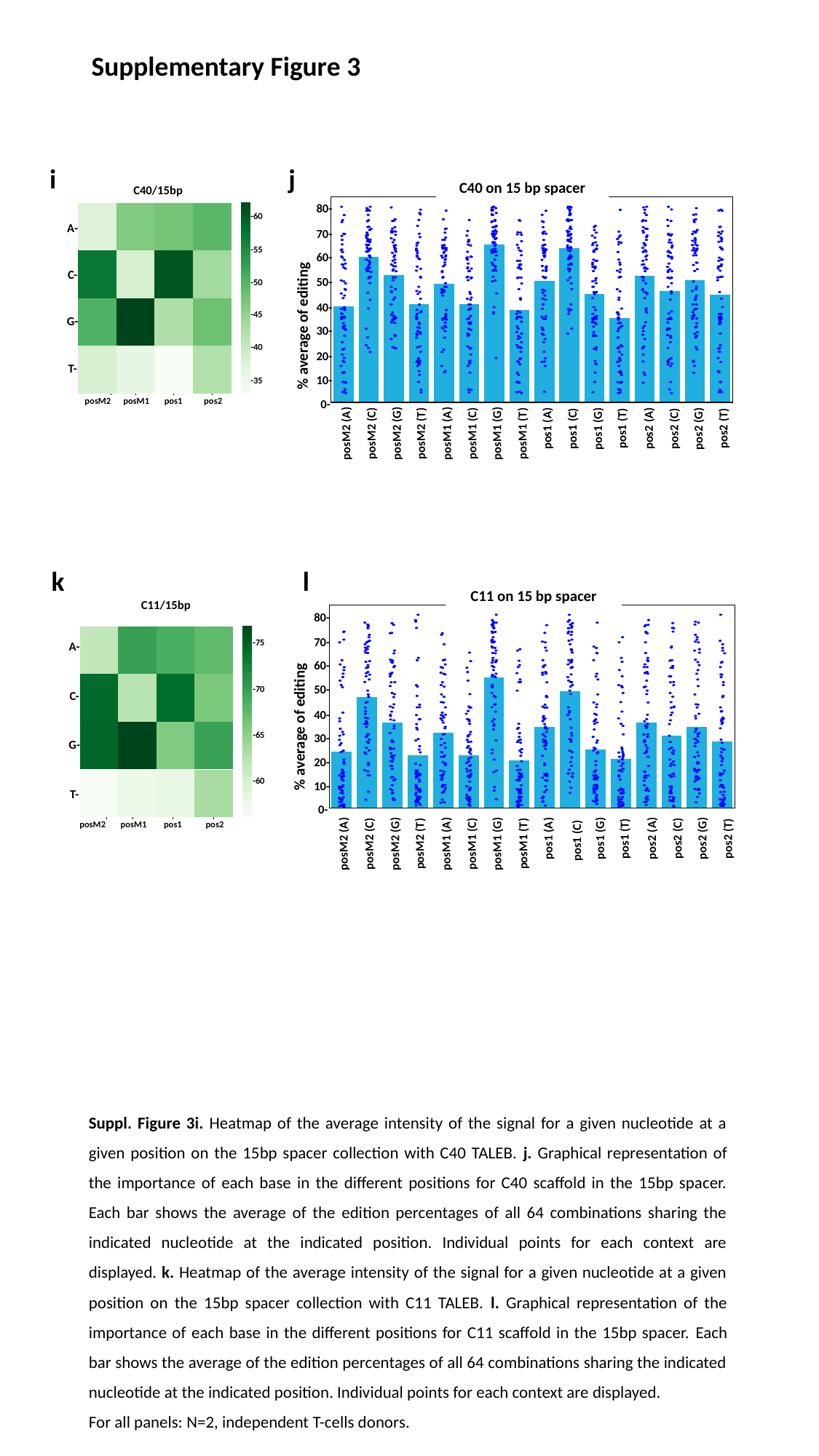

Supplementary Figure 3
j
i
C40 on 15 bp spacer
80-
70-
60-
50-
40-
30-
20-
10-
0-
% average of editing
pos1 (A)
pos1 (C)
pos1 (G)
pos1 (T)
pos2 (A)
pos2 (C)
pos2 (G)
pos2 (T)
posM2 (A)
posM2 (C)
posM2 (G)
posM2 (T)
posM1 (A)
posM1 (C)
posM1 (G)
posM1 (T)
C40/15bp
-60
-55
-50
-45
-40
-35
A-
C-
G-
T-
posM2
posM1
pos1
pos2
k
l
C11 on 15 bp spacer
80-
70-
60-
50-
40-
30-
20-
10-
0-
% average of editing
pos1 (A)
pos1 (G)
pos1 (T)
pos2 (A)
pos2 (C)
pos2 (G)
pos2 (T)
pos1 (C)
posM2 (A)
posM2 (C)
posM2 (G)
posM2 (T)
posM1 (A)
posM1 (C)
posM1 (G)
posM1 (T)
C11/15bp
-75
-70
-65
-60
A-
C-
G-
T-
posM2
posM1
pos1
pos2
Suppl. Figure 3i. Heatmap of the average intensity of the signal for a given nucleotide at a given position on the 15bp spacer collection with C40 TALEB. j. Graphical representation of the importance of each base in the different positions for C40 scaffold in the 15bp spacer. Each bar shows the average of the edition percentages of all 64 combinations sharing the indicated nucleotide at the indicated position. Individual points for each context are displayed. k. Heatmap of the average intensity of the signal for a given nucleotide at a given position on the 15bp spacer collection with C11 TALEB. l. Graphical representation of the importance of each base in the different positions for C11 scaffold in the 15bp spacer. Each bar shows the average of the edition percentages of all 64 combinations sharing the indicated nucleotide at the indicated position. Individual points for each context are displayed.
For all panels: N=2, independent T-cells donors.

## Slide 6
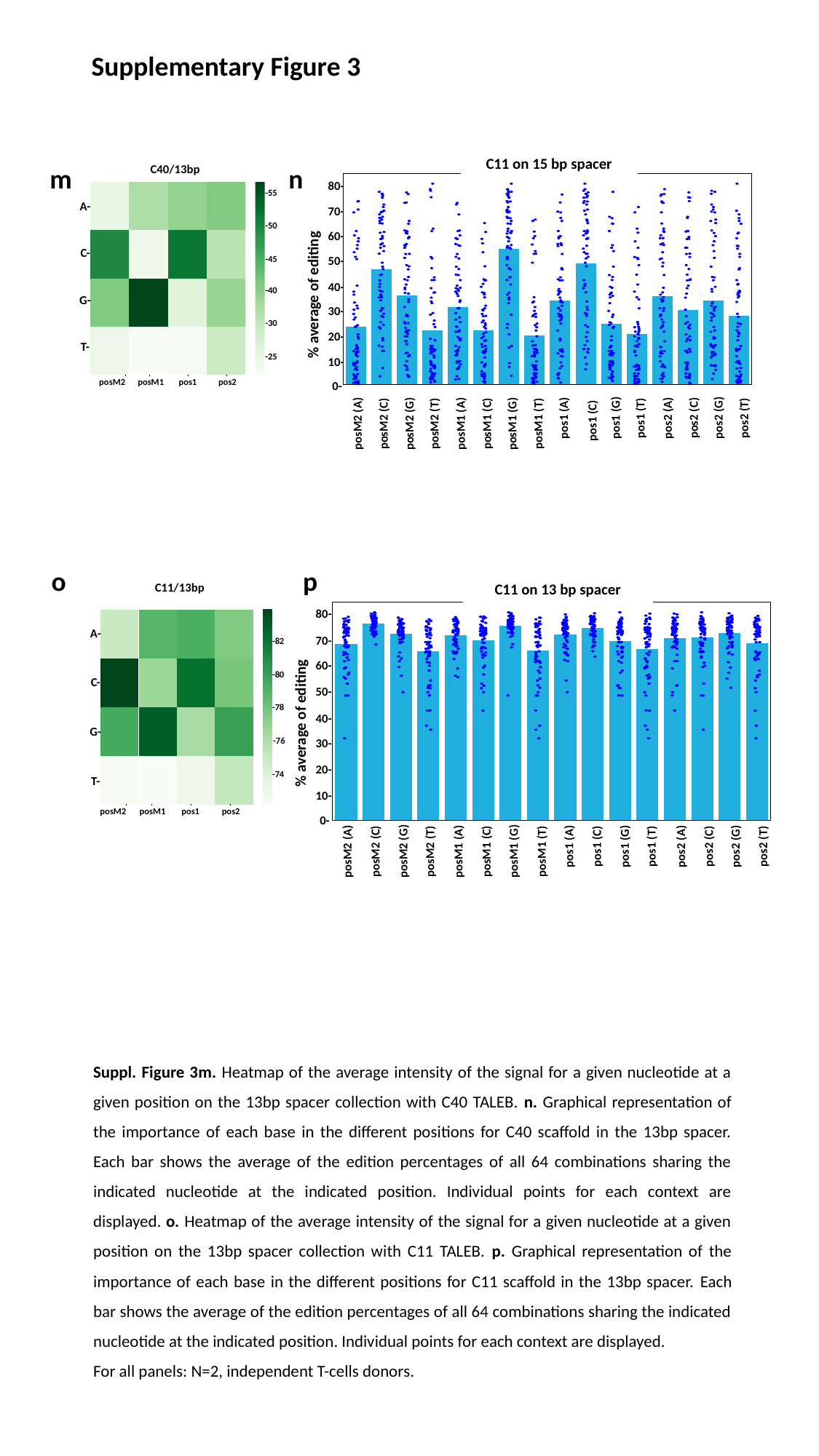

Supplementary Figure 3
C11 on 15 bp spacer
80-
70-
60-
50-
40-
30-
20-
10-
0-
% average of editing
pos1 (A)
pos1 (G)
pos1 (T)
pos2 (A)
pos2 (C)
pos2 (G)
pos2 (T)
pos1 (C)
posM2 (A)
posM2 (C)
posM2 (G)
posM2 (T)
posM1 (A)
posM1 (C)
posM1 (G)
posM1 (T)
n
m
C40/13bp
-55
-50
-45
-40
-30
-25
A-
C-
G-
T-
posM2
posM1
pos1
pos2
o
p
C11 on 13 bp spacer
80-
70-
60-
50-
40-
30-
20-
10-
0-
% average of editing
pos1 (A)
pos1 (C)
pos1 (G)
pos1 (T)
pos2 (A)
pos2 (C)
pos2 (G)
pos2 (T)
posM2 (A)
posM2 (C)
posM2 (G)
posM2 (T)
posM1 (A)
posM1 (C)
posM1 (G)
posM1 (T)
C11/13bp
A-
C-
G-
T-
-82
-80
-78
-74
-76
posM2
posM1
pos1
pos2
Suppl. Figure 3m. Heatmap of the average intensity of the signal for a given nucleotide at a given position on the 13bp spacer collection with C40 TALEB. n. Graphical representation of the importance of each base in the different positions for C40 scaffold in the 13bp spacer. Each bar shows the average of the edition percentages of all 64 combinations sharing the indicated nucleotide at the indicated position. Individual points for each context are displayed. o. Heatmap of the average intensity of the signal for a given nucleotide at a given position on the 13bp spacer collection with C11 TALEB. p. Graphical representation of the importance of each base in the different positions for C11 scaffold in the 13bp spacer. Each bar shows the average of the edition percentages of all 64 combinations sharing the indicated nucleotide at the indicated position. Individual points for each context are displayed.
For all panels: N=2, independent T-cells donors.

## Slide 7
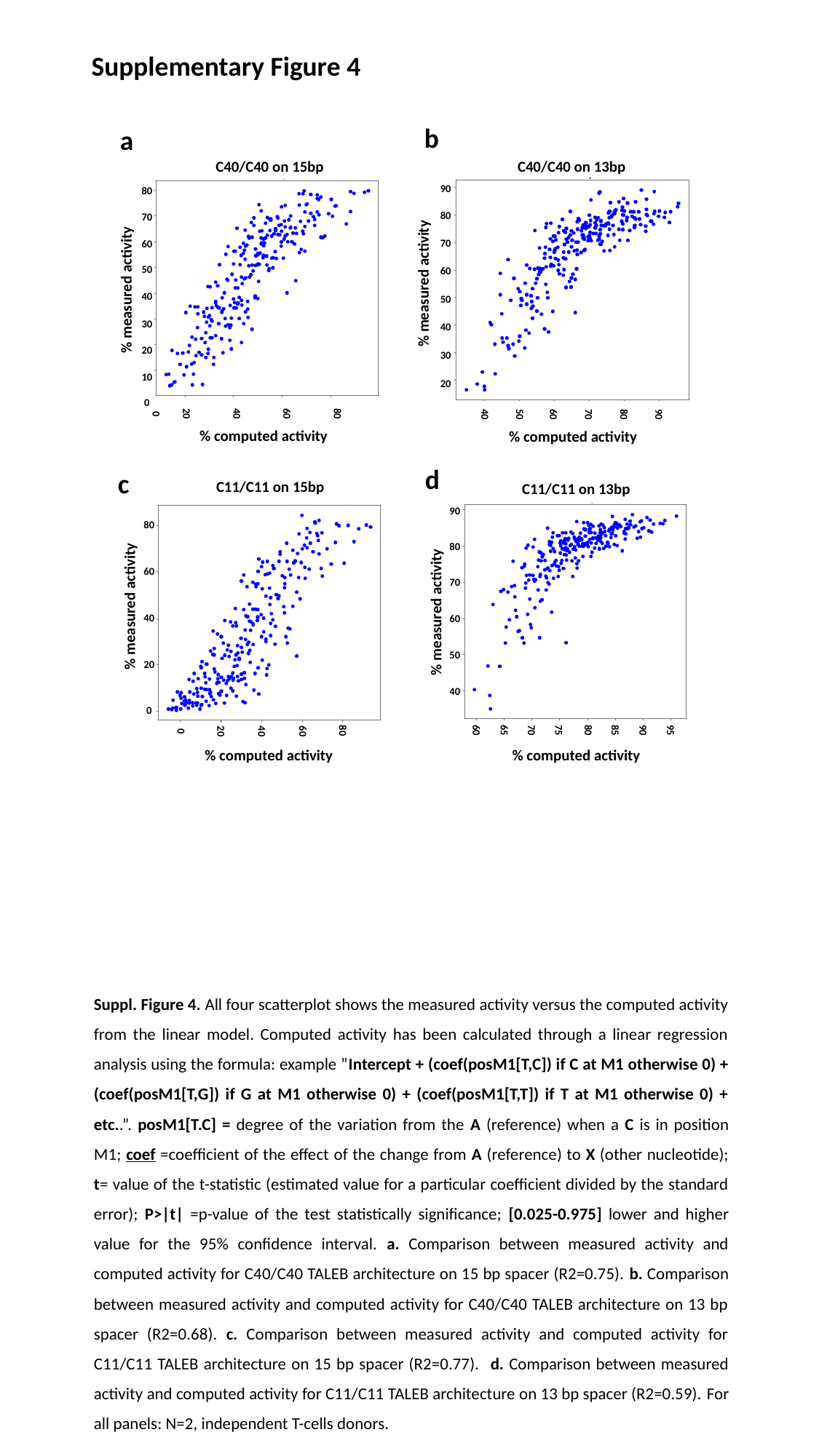

Supplementary Figure 4
b
a
C40/C40 on 15bp
80
70
60
50
40
30
20
10
0
% measured activity
80
60
40
20
0
% computed activity
C40/C40 on 13bp
90
80
70
60
50
40
30
20
% measured activity
90
80
60
50
40
70
% computed activity
d
c
C11/C11 on 15bp
80
60
40
20
0
% measured activity
60
40
20
0
80
% computed activity
C11/C11 on 13bp
90
80
70
50
40
% measured activity
60
60
65
70
75
80
85
90
95
% computed activity
Suppl. Figure 4. All four scatterplot shows the measured activity versus the computed activity from the linear model. Computed activity has been calculated through a linear regression analysis using the formula: example ”Intercept + (coef(posM1[T,C]) if C at M1 otherwise 0) + (coef(posM1[T,G]) if G at M1 otherwise 0) + (coef(posM1[T,T]) if T at M1 otherwise 0) + etc..”. posM1[T.C] = degree of the variation from the A (reference) when a C is in position M1; coef =coefficient of the effect of the change from A (reference) to X (other nucleotide); t= value of the t-statistic (estimated value for a particular coefficient divided by the standard error); P>|t| =p-value of the test statistically significance; [0.025-0.975] lower and higher value for the 95% confidence interval. a. Comparison between measured activity and computed activity for C40/C40 TALEB architecture on 15 bp spacer (R2=0.75). b. Comparison between measured activity and computed activity for C40/C40 TALEB architecture on 13 bp spacer (R2=0.68). c. Comparison between measured activity and computed activity for C11/C11 TALEB architecture on 15 bp spacer (R2=0.77). d. Comparison between measured activity and computed activity for C11/C11 TALEB architecture on 13 bp spacer (R2=0.59). For all panels: N=2, independent T-cells donors.

## Slide 8
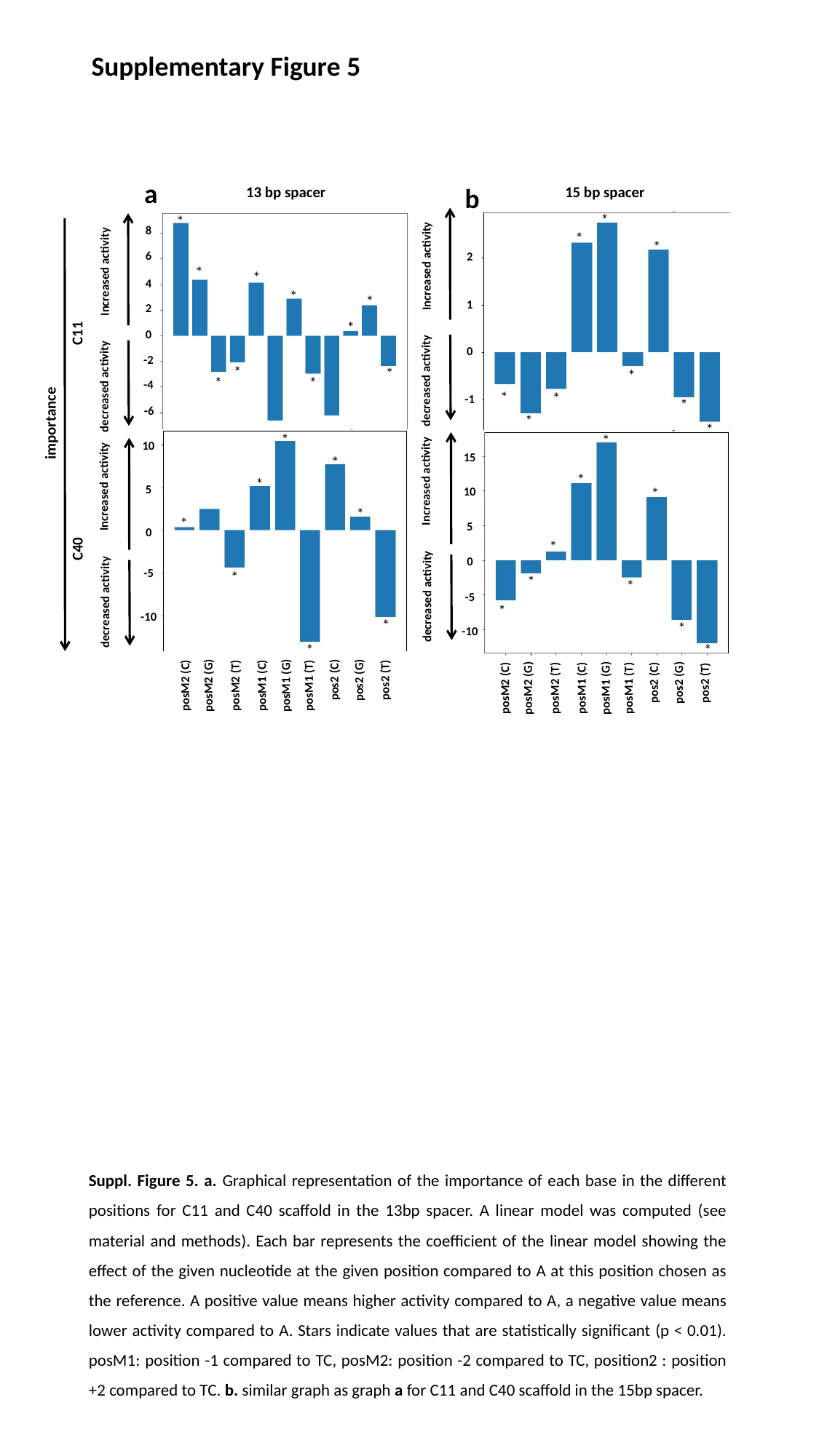

Supplementary Figure 5
a
13 bp spacer
Increased activity
decreased activity
Increased activity
decreased activity
*
8
6
4
2
0
-2
-4
-6
*
*
*
*
*
*
*
*
*
C11
importance
C40
*
*
*
10
5
0
-5
-10
*
*
*
*
*
*
*
pos2 (C)
pos2 (G)
pos2 (T)
posM2 (C)
posM2 (G)
posM2 (T)
posM1 (C)
posM1 (G)
posM1 (T)
b
15 bp spacer
Increased activity
decreased activity
Increased activity
decreased activity
*
2
1
0
-1
*
*
*
*
*
*
*
*
*
15
10
5
0
-5
-10
*
*
*
*
*
*
*
*
pos2 (C)
pos2 (G)
pos2 (T)
posM2 (C)
posM2 (G)
posM2 (T)
posM1 (C)
posM1 (G)
posM1 (T)
Suppl. Figure 5. a. Graphical representation of the importance of each base in the different positions for C11 and C40 scaffold in the 13bp spacer. A linear model was computed (see material and methods). Each bar represents the coefficient of the linear model showing the effect of the given nucleotide at the given position compared to A at this position chosen as the reference. A positive value means higher activity compared to A, a negative value means lower activity compared to A. Stars indicate values that are statistically significant (p < 0.01). posM1: position -1 compared to TC, posM2: position -2 compared to TC, position2 : position +2 compared to TC. b. similar graph as graph a for C11 and C40 scaffold in the 15bp spacer.

## Slide 9
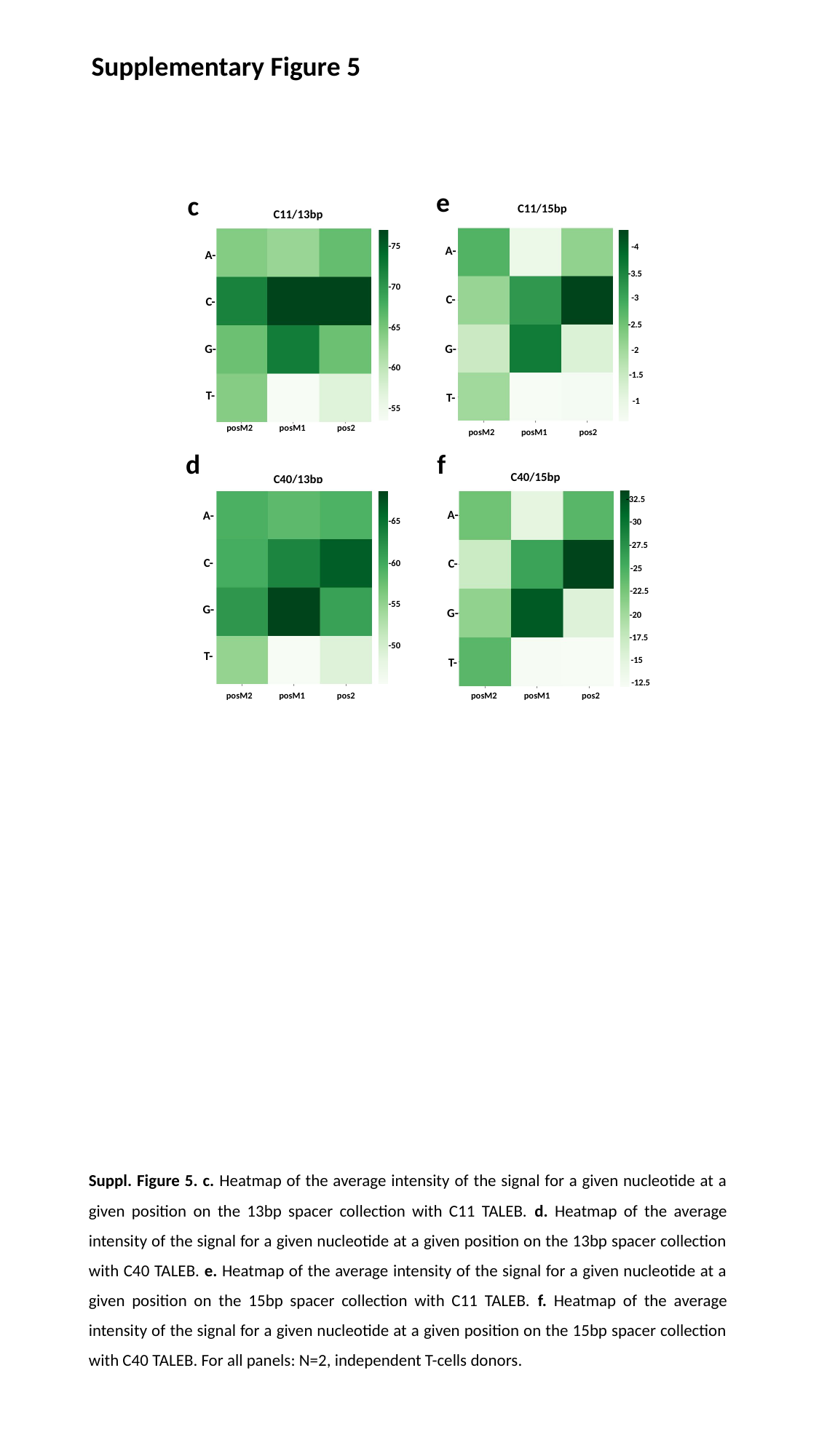

Supplementary Figure 5
e
C11/15bp
-4
-3.5
-3
-2.5
-2
-1.5
-1
A-
C-
G-
T-
posM2
posM1
pos2
c
C11/13bp
-75
-70
-65
-60
-55
A-
C-
G-
T-
posM2
posM1
pos2
d
C40/13bp
-65
-60
-55
-50
A-
C-
G-
T-
posM2
posM1
pos2
f
C40/15bp
-32.5
-30
-27.5
-25
-22.5
-20
-17.5
-15
-12.5
A-
C-
G-
T-
posM2
posM1
pos2
Suppl. Figure 5. c. Heatmap of the average intensity of the signal for a given nucleotide at a given position on the 13bp spacer collection with C11 TALEB. d. Heatmap of the average intensity of the signal for a given nucleotide at a given position on the 13bp spacer collection with C40 TALEB. e. Heatmap of the average intensity of the signal for a given nucleotide at a given position on the 15bp spacer collection with C11 TALEB. f. Heatmap of the average intensity of the signal for a given nucleotide at a given position on the 15bp spacer collection with C40 TALEB. For all panels: N=2, independent T-cells donors.
